# Genomic analysis offers insights into the evolution of the bovine TRA/TRD locus

**DOI:** 10.1186/1471-2164-15-994

**Published:** 2014-11-19

**Authors:** Timothy K Connelley, Kathryn Degnan, Cassandra W Longhi, W Ivan Morrison

**Affiliations:** The Roslin Institute and Royal (Dick) School of Veterinary Studies, The University of Edinburgh, Easter Bush, Midlothian, EH25 9RG Scotland, UK; The Moredun Group, Pentlands Science Park, Bush Loan, Penicuik, Midlothian, EH26 0PZ Scotland, UK

**Keywords:** Bovine, TRA/TRD locus, TR, Duplication, Evolution

## Abstract

**Background:**

The TRA/TRD locus contains the genes for V(D)J somatic rearrangement of TRA and TRD chains expressed by αβ and γδ T cells respectively. Previous studies have demonstrated that the bovine TRA/TRD locus contains an exceptionally large number of TRAV/TRDV genes. In this study we combine genomic and transcript analysis to provide insights into the evolutionary development of the bovine TRA/TRD locus and the remarkable TRAV/TRDV gene repertoire.

**Results:**

Annotation of the UMD3.1 assembly identified 371 TRAV/TRDV genes (distributed in 42 subgroups), 3 TRDJ, 6 TRDD, 62 TRAJ and single TRAC and TRDC genes, most of which were located within a 3.5 Mb region of chromosome 10. Most of the TRAV/TRDV subgroups have multiple members and several have undergone dramatic expansion, most notably TRDV1 (60 genes). Wide variation in the proportion of pseudogenes within individual subgroups, suggest that differential ‘birth’ and ‘death’ rates have been used to form a functional bovine TRAV/TRDV repertoire which is phylogenetically distinct from that of humans and mice. The expansion of the bovine TRAV/TRDV gene repertoire has predominantly been achieved through a complex series of homology unit (regions of DNA containing multiple gene) replications. Frequent co-localisation within homology units of genes from subgroups with low and high pseudogene proportions suggest that replication of homology units driven by evolutionary selection for the former may have led to a ‘collateral’ expansion of the latter. Transcript analysis was used to define the TRAV/TRDV subgroups available for recombination of TRA and TRD chains and demonstrated preferential usage of different subgroups by the expressed TRA and TRD repertoires, indicating that TRA and TRD selection have had distinct impacts on the evolution of the TRAV/TRDV repertoire.

**Conclusion:**

Both TRA and TRD selection have contributed to the evolution of the bovine TRAV/TRDV repertoire. However, our data suggest that due to homology unit duplication TRD selection for TRDV1 subgroup expansion may have substantially contributed to the genomic expansion of several TRAV subgroups. Such data demonstrate how integration of genomic and transcript data can provide a more nuanced appreciation of the evolutionary dynamics that have led to the dramatically expanded bovine TRAV/TRDV repertoire.

**Electronic supplementary material:**

The online version of this article (doi:10.1186/1471-2164-15-994) contains supplementary material, which is available to authorized users.

## Background

Adaptive immunity in jawed vertebrates is mediated by B cells and T cells, two lymphocyte subsets characterised by the expression of highly diverse repertoires of heterodimeric antigen-specific receptors – immunoglobulins (IG or B cell receptors) and T cell receptors (TR). Immunoglobulins are formed from heavy (IgH) and light (either Igκ or Igλ) chains and TR from either α and β chains (αβ T cells) or γ and δ TR chains (γδ T cells).

The variable domains of individual IG and TR chains are formed in lymphocyte precursors by somatic recombination of single discontiguous variable (V), diversity (D – in IgH, TRB and TRD chains only) and joining (J) genes selected from multiple genomic copies of these genes. This recombination is directed by the products of the RAG1/2 genes which introduce double-strand breaks at recombination signal (RS) sequences flanking V, D and J genes [1] that are subsequently repaired by non-homologous joining of the cleaved V(D)J elements. After recombination the V(D)J product is spliced to a relatively invariant constant (C) gene. The different permutations of recombined V(D)J genes used to form individual IG and TR chains generate diversity (combinatorial diversity) that is further amplified by the activities of exonuclease and terminal deoxynucleotide transferase which together modify the germline nucleotide sequence at the V(D)J junction (junctional diversity). Together these mechanisms enable the generation of hugely diverse IG and TR repertoires – for example, the potential repertoire of unique human αβ TR has been calculated to be 10^15^[2]. Highly diverse IG and TR repertoires are crucial to facilitating recognition of the vast array of antigens to which hosts may be exposed and are therefore considered critical to an effective adaptive immune system [3].

The constituent genes used to construct TR chains are located in 3 distinct chromosomal loci - TRB for TRB, TRG for TRG and TRA/TRD for both TRA and TRD chains. Sequencing of murine and human TR loci has enabled the full complement of the TR genes in these species to be described (e.g. [4]) and allowed the organisation and regulation of these immunologically significant loci to be analysed (reviewed in [5, 6]). Additionally, inter-species and inter-loci comparisons have provided insights into how these loci have evolved (e.g. [7]). Between mammalian species the orthologous TR loci generally demonstrate conservation of overall structure – for example the organisation of genes in the TRB locus is similar in humans [4], mice [7], cattle [8] and dog [9]. Despite this conservation there are marked inter-species differences in both the total number of V genes and the relative representation of individual V gene subgroups (genes sharing ≥75% nucleotide identity) located in orthologous loci, indicating that dynamic evolutionary development of V gene germline repertoires has occurred post-speciation. TR V genes follow a ‘birth and death’ model of evolution whereby new genes are generated by repeated duplication (and form the substrate for diversification by mutation), of which some are retained whilst others are deleted or become pseudogenes following acquisition of deleterious mutations [10–12]. Dramatic evolutionary modifications of functional V gene germline repertoires are assumed to reflect selective pressures exerted on the adaptive immune system and in turn result in beneficial changes in the expressed TR repertoires.

The TRA/TRD locus is unique in containing the TR gene elements for 2 TR chains. In humans the TRA/TRD locus spans ~1 Mb and contains 49 TRAV genes, 5 dual usage TRAV/TRDV genes, 3 TRDV, 3 TRDD, 61 TRAJ, 4 TRDJ and single TRAC and TRDC genes, whilst in mice the TRA/TRD locus occupies ~1.7 Mb and contains 88 TRAV, 10 dual usage TRAV/TRDV genes, 6 TRDV, 2 TRDD, 60 TRAJ, 2 TRDJ and single TRAC and TRDC genes [13]. Comparative genomics show that the human and murine TRA/TRD loci share a similar gene organisation with most of the V gene segments located at the 5′ end of the locus and the other TRA/TRD genes and a single TRDV gene with an inverted orientation located at the 3′ end [7]. Two studies have analysed the genomic sequence of the bovine TRA/TRD locus [14, 15]. However, these studies were largely restricted to cataloguing the repertoires of either TRD [14] or TRAV/TRDV [15] genes, and an annotation of all of the TR genes within the locus has yet to be completed. Furthermore, although a main finding in both studies was the dramatic expansion of the V gene repertoires (a total of >400 TRAV/TRDV genes were identified), analysis of the duplication events that generated this hugely expanded germline repertoire, which could inform our understanding of how the bovine TRAV/TRDV repertoire has evolved, have not been conducted.

In this study, we use the most recent assembly of the bovine genome (UMD3.1 [16]) to re-examine the genomic sequence of the TRA/TRD locus. We sought to complete a comprehensive annotation of the TRA/TRD gene repertoire and use this as a basis to fulfil our primary aim of examining the evolutionary development of the massive expansion of bovine TRAV/TRDV genes. By combining analysis of the genome assembly and TRA/TRD transcripts we present data indicating that i) the majority of the TRAV/TRDV gene expansion is attributable to a complex series of duplications of multiple homology units (i.e. regions of DNA incorporating multiple genes), ii) expansion of some TRAV subgroups may have occurred ‘collaterally’, as a consequence of selection for expanded functional repertoires of other subgroups (most notably TRDV1), and iii) the expressed TRA and TRD repertoires preferentially use different TRAV/TRDV subgroups, suggesting TRA and TRD selection have made distinct contributions to the evolutionary development of the genomic TRAV/TRDV repertoire.

## Results

### Annotation of the TRA/D gene repertoire

A prerequisite of our study was accurate, up-to-date information on the bovine genomic TRA/TRD gene repertoire. Annotation of the UMD3.1 bovine genome assembly identified a total of 371 TRAV/TRDV, 3 TRDJ, 6 TRDD, 62 TRAJ, 1 TRAC and 1 TRDC genes. All of these TR genes, with the exception of 12 TRAV/TRDV genes, were located within a 3.5 Mb region of chromosome 10 (Additional file 1).

Phylogenetic analysis indicated the existence of 42 distinct bovine TRAV/TRDV gene subgroups (Additional file 2). Of these, 35 were orthologous to human TRAV subgroups and 3 to human TRDV subgroups and therefore were provisionally assigned the corresponding numbers (see Methods). Of the remaining 4 subgroups, one contained a single gene with 73.3% nucleotide identity with murine TRDV4 and another contained 7 genes showing 72.7-75.8% nucleotide identity with murine TRAV4 – these were designated as bTRDVY and bTRAVY respectively. The other 2 subgroups lacked directly orthologous human or murine subgroups. Genes from 1 of these subgroups had been previously described as bTRDV3 genes [14, 17] – we have re-assigned this subgroup as bTRDVb3 to distinguish it from the bovine TRDV3 subgroup. Subsequent cDNA analyses (see below) demonstrated that members of the final subgroup recombined with TRAJ genes – we consequently designated this subgroup as bTRAVX. All but 8 of the TRAV/TRDV subgroups (TRAV1, 27, 35, 36, 41, and TRDV2, 3 and Y) are multi-membered and several subgroups (TRAV22, 23, 25, 26, X and TRDV1) have undergone dramatic expansion, containing more than 20 members (Table 1).

**Table 1:** **The human and bovine (UMD3.1) TRAV/TRDV repertoires**

Subgroup	Human				Bovine				
	Total	Functional	Pseudo.	Percentage functional	Total	Functional	Pseudo.	Incomplete	Percentage functional
	(Number of genes)				(Number of genes)				
AV1	2	2		100	1	1			100
AV2	1	1		100	7	5	2		71.4
AV3	1	1†	1†	100/0	7	7			100
AV4	1	1		100	3	2	1		66.7
AV5	1	1		100	2	2			100
AV6	1	1		100	4		3	1	0
AV7	1	1		100					
AV8	7	5	2	71.4	14	6	8		42.9
AV9	2	2		100	11	1	9	1	9.1
AV10	1	1		100	6	3	3		50
AV11	1		1	0	6		6		0
AV12	3	3		100	6	5		1	83.3
AV13	2	2		100	11	8	2	1	72.8
AV14	1	1		100	9	4	4	1	44.4
AV15	1		1	0					
AV16	1	1		100	2	2			100
AV17	1	1		100	3	1	2		33.3
AV18	1	1		100	8	3	5		37.5
AV19	1	1		100	7	5	2		71.4
AV20	1	1		100	5	5			100
AV21	1	1		100	4	3	1		75
AV22	1	1		100	29	14	14	1	48.3
AV23	1	1		100	24	4	20		16.7
AV24	1	1		100	10	1	8	1	10
AV25	1	1		100	22	11	10	1	50
AV26	2	2		100	39	22	13	4	56.4
AV27	1	1		100	1	1			100
AV28	1		1	0	4	3	1		75
AV29	1	1†	1†	100/0	3	2	1		66.7
AV30	1	1		100					
AV31	1		1	0					
AV32	1		1	0					
AV33	1		1	0	6	2	4		33.3
AV34	1	1		100	3		3		0
AV35	1	1		100	1	1			100
AV36	1	1		100	1	1			100
AV37	1		1	0	2		2		0
AV38	2	2		100	6	5		1	83.3
AV39	1	1		100	2	1	1		50
AV40	1	1		100					
AV41	1	1		100	1	1			100
AVX					29	23	5	1	79.3
AVY					7	7			100
DV1	1	1		100	60	48	8	4	80.0
DV2	1	1		100	1	1			100
DV3	1	1		100	1	1			100
DVb3					2	2			100
DVY					1	1			100
Total	57	46 (+2)	9 (+2)	80.7	371	215	138	18	58.0

The numbers of total, functional and pseudogenes in each TRAV/TRDV subgroup in the germline human and bovine (UMD3.1) repertoires are shown. The percentage of genes in each subgroup that are functional has been calculated (for human genes that have alternative functional and non-functional alleles – denoted by †, two percentage values are shown). For the bovine TRAV/TRDV subgroups, the number of genes for which only incomplete genomic sequence was available is also shown. Details of the human TRAV/TRDV repertoire were taken from the IMGT database (http://www.imgt.org).

Although our findings are broadly consistent with the findings from previous annotations [14, 15] there are several features that make this annotation distinct: i) the number of TRAV/TRDV genes identified is lower than in previous annotations (Additional file 3), ii) several novel subgroups have been identified, iii) the full length (L exon, V exon and RS) of all TRAV/TRDV genes has been annotated (Additional file 4 and Additional file 5), and iv) all of the TRA/TRD genes have been included. Notably only about half of the TRAV/TRDV genes identified here had 100% sequence identity to genes identified in the Btau_4.0 assembly (Additional file 3).

### The structure of the 3′ end of the bovine TRA/TRD locus shows syntenic conservation with that of humans and mice

**Figure 1:**
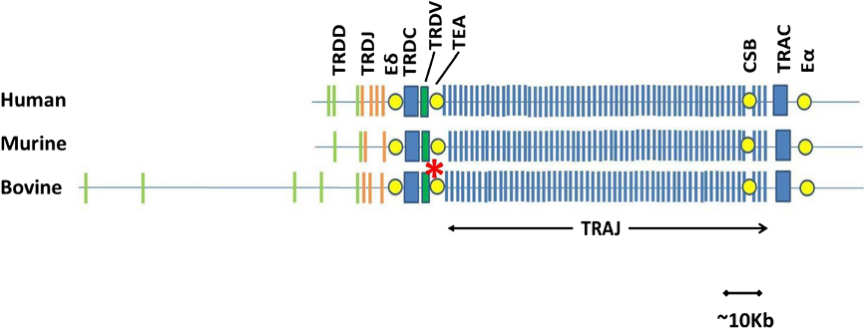
**Conserved synteny of the 3′ end of the bovine, human and murine TRA/TRD loci.** A schematic representation shows the conserved inter-species organisation of TR genes and regulatory elements at the 3′ end of the TRA/TRD locus. The approximate positions of TRDD (green lines), TRDJ (orange lines), TRAJ (blue lines), TRDC and TRAC (blue blocks), the single inverted TRDV gene (TRDV3 in bovine and human, TRDV5 in mouse – green block) and regulatory elements (yellow circles) are shown. The regulatory elements represented are – TRA enhancer (Eα), TRD enhancer (Eδ), T early alpha (TEA) and conserved sequence block (CSB). The length of the region is also conserved across the species, with the exception of the area occupied by TRDD genes, which is much greater in the bovine TRA/TRD locus (~80 Kb) than in the mouse or human (~10 Kb). The asterisk indicates the approximate location of the 1 Kb gap between contigs DAAA02028052.1 and DAAAA02028053.1.

The UMD3.1 assembly of the 3′ end of the bovine TRA/TRD locus appears to be essentially complete. All TRAC, TRAJ, TRDC, TRDJ, TRDD genes and the TRDV3 gene (in inverted orientation) are located on 2 large contigs (DAAA02028052.1 – 140 Kb and DAAAA02028053.1 – 114 Kb) separated by a gap of approximately 1 Kb. The organisation of the genes demonstrates a marked syntenic conservation between mouse, human and bovine (Figure 1). Synteny is maintained within the TRAJ repertoire with individual bovine TRAJ genes sharing a conserved order with their murine and human orthologues (Additional file 6). Additionally there is conservation of important regulatory elements at the 3′ end of the TRA/TRD locus, with bovine sequences showing identity with the human/murine TRA enhancer (Eα), TRD enhancer (Eδ), CSB and TEA located in syntenic positions (Figure 1 and Additional file 1 and Additional file 7). In addition to gene content and organisation, the size of the 3′ end of TRA/TRD locus is similar across the 3 species (approximately 100 Kb from TRDJ to TRAC), with the exception of the TRDD region which in bovine is ~80 Kb – approximately 8× the size of the equivalent regions in the mouse and human TRA/TRD loci.

### Expansion of the bovine TRAV/TRDV repertoire has predominantly occurred via duplication of homology units

The UMD3.1 assembly of the 5′ end of the locus is highly fragmented with the ~3.3 Mb of sequence distributed across >250 separate contigs. Although this fragmentation precludes a description of the overall genomic structure of the 5′ end of the locus, regions of substantial contiguous sequence (up to 198 Kb) enable examination of the organisation of sections of the TRAV/TRDV region. Within these sections small areas demonstrating syntenic conservation with human TRAV/TRDV genes were identified (indicated by juxtaposition of genes from consecutively numbered TRAV/TRDV subgroups – Additional file 1). However, a more prominent feature of the TRAV/TRDV gene organisation within these contigs was recurrent patterns of intercalation of genes from the multi-membered subgroups. For example the ordered sequence of TRAV12-11-10-9 genes recurs 6 times in the UMD3.1 assembly (Additional file 1), suggesting that replication of DNA segments containing multiple TRAV/TRDV genes has led to the formation of ‘homology units’. Detailed nucleotide identity analysis of these regions indicated the presence of 10 putative homology units incorporating between 2 and 17 TRAV/TRDV genes and extending in size from ~15 Kb to >150 Kb (Table 2 and Additional file 8).

**Table 2:** **Putative bovine TRAV/TRDV gene homology units identified in the UMD3.1 assembly**

Homology unit	TRAV/TRDV gene motif	No. of TRAV/TRDV genes	Size (Kb)	Number of replicons	Variable gene content	
					Proportion of homology unit amplified	Post-replication insertion/deletion
1	33-29-28-33-34-26-33	7	39	2		Yes
2	2-3	2	15	7		
3	38-37	2	15	2		
4	14-13-9	3	15	5		
5	22-8-21-20-19-X-X-X-18-12-11-10-9-14-13-Y-9	17 (15)	157	6	Yes	
6	25-19-X-X-X-X-18-17-16	9 (8)	80	3		
7	DV1-22-26-25-DV1-23-22-8-21-20	10	71	5		
8	DV1-23-22-26-DV1-25-23-22-26-DV1-25	11	63	8	Yes	Yes
9	DV1-22-8-DV1-26-25-24-DV1-26-DV1-26	12 (8)	86	7	Yes	Yes
10	DV1-23-22-24-DV1-23	6	43	2		

For each homology unit the TRAV/TRDV gene motif, the number of genes, the estimated minimal size and the number of replicons identified are shown. Due to the fragmentary nature of the UMD3.1 assembly many of the homology units presented here and many of the copies of the homology units are likely to be incomplete. Therefore the TRAV/TRDV gene motif, number of TRAV/TRDV genes and size of homology units should be considered a minimum. Similarly, we have rationalised the number of replicons for each homology unit to the minimum that could account for all of the partial replications observed in the UMD3.1 assembly and so this should also be considered a minimum. For homology units 5, 6 and 9 no single copy covers the entirety of the homology unit and we have estimated its size by combining data from multiple copies; the number of genes in the most extensive copy of these homology units is indicated in parentheses in the ‘No. of TRAV/TRDV genes’ column. In addition we have summarised where there is evidence in the UMD3.1 assembly of the TRAV/TRDV gene content of copies of a homology unit differing due to either varying proportions of the homology unit being duplicated in different replicative iterations or post-replication insertion/deletion (see Additional file 8).

Within the context of homology unit replication the expansion of some multi-membered subgroups appears evolutionarily simple. For example, amplification of the TRAV2 and 3 subgroups is due to 6 tandem replications of homology unit 2, resulting in a ~140 Kb region with alternating TRAV2 and TRAV3 genes at the 5′ end of the locus. In contrast, expansion of other subgroups, especially those that have undergone prolific expansion, appears to be evolutionarily complex. TRDV1 subgroup members are found on 4 different homology units (homology units 7, 8, 9 and 10), intercalated with different permutations of genes including members of the TRAV22, 23, 25 and 26 subgroups. The presence of multiple copies of genes from some subgroups (e.g. up to 3 copies of TRDV1 in homology unit 9) amplifies the expansive effect of homology unit replication on the size of these subgroups. Notably TRAVX expansion is also largely a product of members being incorporated into multiple homology units (units 5 and 6) and the presence of multiple members of this subgroup being present in the homology units. Most of the homology units have undergone multiple replicative events (Table 2) and in some cases variable proportions of the unit appear to have been duplicated (e.g. homology units 5.6 and 5.7 appear to represent a variant in which the TRAV9-14-13-Y-9 component of the homology unit has been excluded – Additional file 9) whilst others display evidence of post-replication deletion/insertion events modifying the gene content of some replicons. For example, the 2 copies of homology unit 1 show disparity in the number of TRAV28 genes present (Additional file 9).

The location of 288 out of the 371 (77.6%) TRAV/TRDV genes identified in the UMD3.1 assembly within homology units attests to replication of homology units being the principal mechanism employed in achieving the massively expanded bovine TRAV/TRDV repertoire. The multiplicity of identified homology units, the variation in replicon number and the modifications of homology unit content during and after replication suggest the evolutionary development of the bovine TRAV/TRDV repertoire has been highly complex.

### Extensive duplication of the TRDV1 subgroup has skewed the phylogenetic representation of the functional TRAV/TRDV gene repertoire of cattle

The functional competency of V, D and J genes is dependent on the maintenance of a correct open reading frame (ORF), the presence of codons encoding certain critical amino acid residues and the presence of a RS. *In silico* sequence analysis based on these parameters indicates that all TRDD and TRDJ genes and 52 out of 62 (83.9%) of TRAJ genes are functional (Additional file 10) and so the repertoire size of these genes is fairly comparable to that seen in mice and humans (Table 3).

**Table 3:** **Repertoire of functional TRA/TRD gene segments in the bovine (UMD3.1), murine and human genomes**

	Bovine (UMD3.1)	Human	Murine
TRAV/TRDV	215 (38)	48* (37)	87* (23)
TRAJ	52	50	38
TRDD	5	3	2
TRDJ	3	4	2

*For human and murine TRAV/TRDV repertoires we represent the maximum number of potential functional genes (i.e. including TRAV/TRDV gene segments for which both functional and non-functional alleles exist as functional). The numbers in parentheses show the number of TRAV/TRDV subgroups that include functional members.

Of the 371 TRAV/TRDV genes, 215 (58.0%) were predicted to encode functional products and 138 (37.2%) pseudogenes (Additional file 11), whilst the functional competency of the remaining 18 (4.8%) genes could not be assessed as the sequences were incomplete (Table 1). The predicted bovine functional TRAV/TRDV repertoire incorporates genes from 38 subgroups and is approximately 2.5 and 4 times larger than that of mice and humans respectively. Between multi-membered subgroups there is huge disparity in the proportion of genes that are functional - some having a high proportion of pseudogenes (e.g. TRAV9 and TRAV11) whilst in others (e.g. TRAV3 and TRAVY) every gene is predicted to be functional. This extends to the massively expanded subgroups, with TRAVX and TRDV1 subgroups retaining a high proportion of functional genes (~80%) and TRAV23 having a low proportion (~15%). Notably members of subgroups with high proportions of pseudogenes are generally co-localised within homology units with genes from subgroups with low pseudogene proportions: for example many TRAV23, 24 and 8 pseudogenes are found with TRDV1 genes on homology units 7–10 (Additional file 9). Such juxtaposition suggests that homology unit replication due to selection for expansion of some subgroups (e.g. TRDV1) may have co-incidentally led to the expansion of other TRAV/TRDV subgroups (e.g. TRAV23, 24 and 8) for which there has been no evolutionary selection to generate/maintain an expanded functional genomic repertoire.

**Figure 2:**
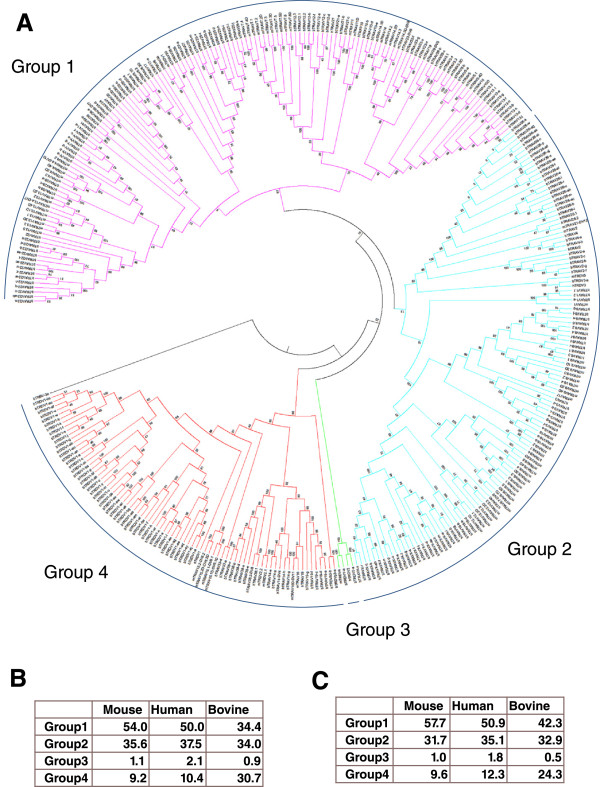
**Phylogenetic analysis of the repertoires of functional TRAV/TRDV genes in mice, humans and cattle.** **(A)** - Neighbour-joining phylogenetic tree of all functional murine, human and bovine (from the UMD3.1 assembly) TRAV/TRDV genes. Analysis of the nucleotide sequence of the V-REGION (IMGT nomenclature) following pairwise deletion to remove gaps in the alignment. The final dataset included 340 positions. The sequence of bTRBV3a was used to root the tree. Based on 1000 boot strap replicates support for Groups 1 (purple), 3 (green) and 4 (red) was high with P_B_ >97%. Support for Group 2 (blue) was low (P_B_ = 13%) but examination of the data using UPMGA and minimum evolution models generated the same phylogenic groups, suggesting it was reliable (data not shown). h = human, b = bovine and m = murine. Percentage of the functional **(B)** and total **(C)** TRAV/TRDV genes in humans, mice and cattle in the 4 phylogenetic Groups defined from neighbour-joining analysis.

Neighbour-joining analysis resolved functional murine, human and bovine TRAV/TRDV genes into 4 monophylogenic groups (Figure 2). In mice and humans there is marked inter-species similarity in the distribution between these monophylogenic groups, with Groups 1, 2, 3 and 4 containing approximately 50%, 35%, 1% and 10% of the functional TRAV/TRDV genes respectively in both species (Figure 2B). In contrast, the distribution of functional genes in bovine is different with a relative contraction of Group 1 (34.4%) and expansion of Group 4 (30.7%); the latter due to the high number of TRDV1 genes. In contrast to mice and humans, in which the relative proportions of the 4 Groups within the total gene repertoires (Figure 2C) are similar to that observed in the functional repertoires, in bovine the representation of Group 1 is higher (42.3%) and that of Group 4 is lower (24.3%) in the total than in the functional repertoires. Thus, it is apparent that massive expansion of particular TRAV/TRDV subgroups (most prominently TRDV1) combined with the preferential retention of functionality in some of these subgroups act synergistically to cause a profound phylogenetic shift in the repertoire of functional TRAV/TRDV genes available for rearrangement of TRA/TRD chains.

### Analysis of expressed TRA and TRD repertoires suggests preferential usage of particular TRAV/TRDV subgroups

Although the expressed bovine TRD chain repertoire has been examined extensively in a number of studies [17–21], the published data on the expressed bovine TRA chain repertoire is limited [22, 23]. A search of the NCBI EST archive revealed a restricted number of entries for rearranged TRA chains (80 entries including representatives from 24 different TRAV/TRDV subgroups – Table 4). To facilitate a direct examination of the TRAV/TRDV subgroups contributing to the expressed TRA and TRD chain repertoires, we designed a panel of TRAV/TRDV subgroup-specific 5′ primers that could be used in conjunction with TRAC- and TRDC-specific 3′ primers to amplify rearranged TRA and TRD chains from cDNA. Sequencing of PCR amplicons generated from γδ T cells and αβ T cells demonstrated that; i) all of the TRAV subgroups predicted to include functional members (with the exception of TRAV39) and also TRDV1, TRDV3 and TRDVb3 could rearrange to form functional TRA chains and ii) genes from all of the TRDV subgroups and TRAV33 can functionally rearrange with TRDC. Thus, the expressed TRA and TRD chain repertoires can utilise genes from a minimum of 35 and 6 TRAV/TRDV subgroups respectively.

**Table 4:** **Summary of EST and PCR data on bovine TRAV/TRDV subgroup expression in re-arranged TRA and TRD chains**

TRAV/TRDV subgroup	EST evidence of use in TRA/TRD rearrangement		PCR data (iii)					
			No. of unique TRA/TRD chain sequences (iv)	Total number of unique TRAV/TRDV genes (v)	Identical genome matches		No. of matches >97% (viii)	No. of matches <97% (ix)
					Number (vi)	Matching TRAV/TRDV gene (vii)		
	TRA (i)	TRD (ii)						
AV1	●		5	3	1	A	2	
AV2			4	3	1	G	2	
AV3	●		13	8	5	a, b, c, e, f	3	
AV4			2	2	-		2	
AV5	●		1	1	-			1
AV8	●		9	7	3	I, l, n	3	1
AV9	●		2	2	1	K	1	
AV10	●		5	3	1	B	2	
AV12	●		10	7	1	D	5	1
AV13	●		8	7	3	f, j, k	3	1
AV14			2	2	1	E	1	
AV16	●		4	4	2	a, b	2	
AV17	●		8	4	2	a, c	2	
AV18			2	2	-		2	
AV19			4	2	1	C	1	
AV20			3	1	1	E		
AV21	●		4	2	1	C	1	
AV22	●		11	9	2	aa, w	6	1
AV23	●		3	2	1	X	1	
AV24			1	1	-		1	
AV25	●		5	4	1	v	2	1
AV26	●		5	5	3	aj, s, w	2	
AV27	●		5	3	-		1	2
AV28	●		3	2	2	b, c		
AV29	●		8	4	2	a, b	2	
AV33		●	7*^/^†	6	-		1	5
AV34			-		-		-	
AV35	●		3	2	-		2	
AV36			2	1	1	a		
AV38			10	6	-		5	1
AV39			-					
AV41	●		1	1	-		1	
AVX	●		35	23	11	aa, ab, b, f, g, l, p, q, u, w, x	12	
AVY			10	7	3	d, e, g	4	
DV1	●	●	111*^/^†	45	13	af, ah, ai, as, au, b, bb, e, g, aj, bg, j, w	25	7
DV2			2†	1	1	a		
DV3	●	●	2†	1	1	a		
DVb3		●	2†	6	1	b	5	
DVY			29†	2	1	a	1	
Total			341	191	67		103	21

BLAST analysis of the NCBI *Bos taurus* EST archive (March 2013) using the bovine TRAC and TRDC sequences identified 24 subgroups participating in TRA chain rearrangement (column i) and 4 participating in TRD rearrangement (column ii). Using a combination of bovine TRAV/TRDV subgroup-specific and unbiased SMART TRAC^+^ and TRDC^+^ PCR (column iii) 341 unique full or partial TRA/TRD chain transcripts were sequenced (column iv), providing a more comprehensive description of the breadth of TRAV/TRDV subgroup usage in TRA and TRD transcripts. Amongst these sequences 191 unique TRAV/TRDV gene sequences were identified (column v), of which only 67 (35.1%) matched any of the genomic sequences identified in the UMD3.1 assembly (column vi and vii). Twenty-one of the TRAV/TRDV gene sequences (11%) were sufficiently dissimilar to genomic TRAV/TRDV genes (<97% nucleotide identity) to be considered as products of TRAV/TRDV genes absent from the UMD3.1 assembly (column ix), whilst the other 103 sequences (53.9%) showed >97% identity with genomic sequences and may either represent products of novel TRAV/TRDV genes or allelic variants of the genes in the UMD3.1 assembly (column viii). Despite several attempts no amplification could be achieved with TRAV39 subgroup-specific primers and there was no EST data supporting use of genes from this subgroup in rearranged TRA or TRD chains; consequently this is the only subgroup which was predicted to contain functional TRAV/TRDV genes for which there is no evidence of expression in the TRA/TRD repertoires.

**Figure 3:**
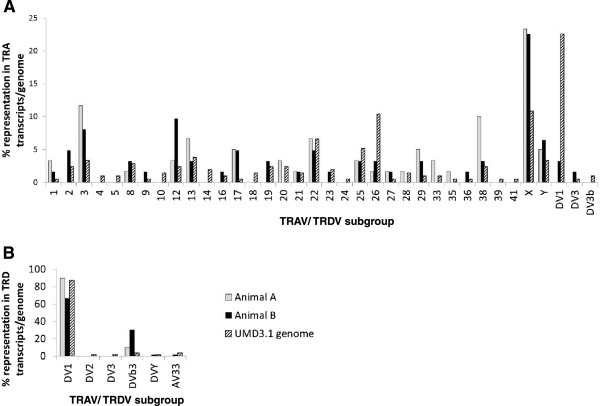
**Usage of TRAV/TRDV subgroups in the expressed TRA and TRD chain repertoires.** cDNA from CD3^+^γδ^-^ (αβ T cells) and CD3^+^γδ^+^ (γδ T cells) populations isolated from the PBMC of 2 animals were subjected to unbiased SMART TRAC^+^/ TRDC^+^ PCR amplification and representative amplicons sequenced. The subgroup of the TRAV/TRDV gene expressed by each TRA and TRD chain sequence that was predicted from *in silico* analysis to be functional (for TRA transcripts n = 60 and 61 and for TRD transcripts n = 68 and 63 for Animals A and B respectively) was identified. The percentage of the TRA transcripts **(A)** and TRD transcripts **(B)** that utilised genes from the different TRAV/TRDV subgroups is shown (grey and black bars) alongside the percentage of the genes in the genomic repertoire available for TRA and TRD rearrangement within each TRAV/TRDV subgroup (striped bars).

To determine at what frequencies the different TRAV/TRDV subgroups were utilised in the expressed TRA and TRD repertoires, we used a SMART RACE-PCR system that enables unbiased amplification of TR chain sequences [24] to analyse TRA and TRD transcripts from αβ and γδ T cells of 2 MHC-disparate animals. TRA chain analysis showed utilisation of a diverse range of TRAV/TRDV subgroups (19 and 23 subgroups) in both animals (Figure 3A). Although most subgroups were represented at low frequencies (<5%), in each animal there was a limited number of subgroups (4 or 5) that constituted a higher proportion of the repertoire. Although these highly represented subgroups generally differed between the 2 individuals, both animals showed a particularly high representation of TRAV3 (11.3% and 8.2%) and TRAVX (23% and 22.6%). Comparison with the functional genomic TRAV/TRDV gene repertoire revealed that these two TRAV/TRDV subgroups, as well as TRAV17, 27 and 29, were relatively over-represented in the expressed TRA chain repertoire in both animals, whilst TRAV26 and TRDV1 were under-represented (Figure 3A). With reference to the phylogenetic groups discussed above, Groups 1 and 2 each accounted for approximately ~45% of the expressed TRA chain sequences whilst the remaining ~10% used genes from Group 4. Thus, in comparison to the genomic repertoire there is a preference for use of Group 1 and 2 genes and bias against use of Group 4 genes.

As anticipated from the TRAV/TRDV subgroup-specific PCR, the TRD chain repertoire in both animals showed a limited use of TRAV/TRDV subgroups. The TRD chain sequences obtained from both animals were dominated by TRDV1 and TRDVb3, which accounted for 90% and 10% of the sequences respectively in one animal and 60% and 32% in the other, with only single additional TRDVY and TRAV33 sequences found in the second animal (Figure 3B). The dominance of TRDV1 observed was consistent with results from previous analysis [21]. With the exception of TRDVY, all of the subgroups detected in the sequenced TRD repertoire belong to phylogenetic Group 4 and consequently the TRD repertoire analysed here is almost completely restricted (>99%) to this Group.

During this study, 198 and 143 unique functional TRA and TRD chains, utilizing a total of 191 distinct TRAV/TRDV genes, were sequenced. Only 67 (35.1%) of the expressed TRAV/TRDV gene sequences were identical to a gene identified from the genome. All of the remainder belonged to already identified subgroups; 21 (11.0%) of them showed <97% identity to one or more genomic sequences (the level of identity conventionally used in mouse and humans to distinguish products of distinct TR V genes [25, 26]) and thus most likely represent products of genes absent from the current assembly, whilst 103 (53.9%) displayed nucleotide identities of between 97.0-99.5% to genomic sequences. Given the presence within the majority of multi-membered subgroups of genomic sequences sharing >97% identity (data not shown), it is not possible to conclude whether these 103 TRAV/TRDV sequences represent allelic variants of genes identified in the genome or products of TRAV/TRDV genes absent from the current assembly. In contrast, the expressed TRAJ and TRDJ genes all corresponded to a genomic sequence – all 3 TRDJ genes and all functional TRAJ genes with the exception of TRAJ1, 6, 8–1, 41, 59, 60 were represented in the expressed TRA/TRD chains analysed.

## Discussion

Gene duplication has been fundamental to the evolution of antigen-specific receptors, leading to the formation of the separate IG/TR loci [7, 27, 28] and the expansion of the repertoire of D, J and most notably V genes within these loci. Inter-loci and inter-species comparisons demonstrate that the extent of duplication that has occurred in different IG/TR loci during their evolution varies dramatically. The large number of bovine TRAV/TRDV genes identified in previous genomic analyses [14, 15] demonstrates that duplication within the bovine TRA/TRD locus has been prolific. In this study we have used genomic and cDNA analyses to investigate the evolutionary basis for this dramatic expansion and its relationship with the expressed TRA and TRD chain repertoires.

The availability of an improved bovine genome assembly was critical to the study. The UMD3.1 assembly is more accurate than the Btau versions with a greater degree of completion, more contiguous sequence, corrections of SNPs and removal of erroneous segmental duplications [16]. Thus, although the catalogue of genes defined in our annotation is in general agreement with previous analyses [14, 15] there are substantive differences. Most significant of these are the identification of genes belonging to 5 novel bovine TRAV/TRDV subgroups (TRAV6, 34 and 37 and TRDV2 and Y) and a reduction in the total number of TRAV/TRDV genes identified (371 vs. 402 in [15]). Comparative analyses indicated that Btau_4.0 included many more TRAV/TRDV genes showing 100% nucleotide identity, suggesting the discrepancy in TRAV/TRDV gene number is largely attributable to the annotation of erroneous duplications in Btau_4.0. However, more pertinent to our study was the improved mapping of genes to the TRA/TRD locus on chromosome 10 and the larger fragments of contiguous TRA/TRD locus sequence. Despite these improvements, the UMD3.1 assembly, like the previous Btau_4.0 and _3.0 assemblies, is i) derived from DNA taken from a father-daughter pairing, which due to the highly polymorphic nature of TRAV/TRDV genes, means that the potential for some proportion of the TRAV/TRDV expansion to be artefactual cannot be excluded, and ii) highly fragmented, with cDNA analysis suggesting that a substantial number of TRAV/TRDV genes are still missing. Consequently, definition of the total bovine TRAV/TRDV repertoire will be dependent on further work to obtain a completed assembly of the bovine TRA/TRD locus.

The high level of similarity in the organisation and gene content of the 3′ end of the bovine, murine and human TRA/TRD loci, which contains the TRAJ, TRDJ, TRAC, TRDC genes and the main regulatory elements, indicates that since the divergence of the primate, rodent and artiodactyl lineages 100 million years ago (MYA) [29] there has been strong conservative evolution of this region. In direct contrast, the huge disparity in the numbers of TRAV/TRDV genes located in the 5′ end of the human, murine and bovine TRA/TRD loci describes a divergent evolutionary development. Expansion of the human TRAV/TRDV repertoire (54 genes) has been limited with only 7 of the 44 TRAV/TRDV subgroups having multiple-members and only TRAV8 (7 genes) having >3 members. In mice a higher proportion of subgroups are multi-membered (14 out of 28) and these are in general larger (TRAV6/TRDV9 is the largest subgroup, comprising 12 members) but the repertoire of 104 TRAV/TRDV genes is still modest in comparison to the 371 genes in cattle. Duplication appears to be a general feature of bovine TRAV/TRDV subgroups, with the majority (34 out of the 42) being multi-membered; however, the more remarkable feature is the extent of duplication within some of the subgroups – TRDV1 has 60 members, and TRAV22, 23, 25, 26 and X all have >20 members. In this respect there are clear parallels with the bovine TRB locus where amplification of the TRBV repertoire is characterised by the prodigious expansion of certain subgroups (TRBV6 and 9) [8].

The location of 77.6% of bovine TRAV/TRDV genes in homology units demonstrates that homology unit replication has been the principal mechanism for expansion of the repertoire. In IG/TR loci expansion of the V gene repertoire by duplication of homology units incorporating genes from multiple subgroups is a common feature [7–9, 30, 31], although the extent to which this has occurred and its complexity differs markedly between loci. The identification in the UMD3.1 assembly of 10 putative homology units, ranging in size from 15 to 157 Kb and containing 2–17 TRAV/TRDV genes, many of which had undergone repeated replication, shows that the expansion of the bovine TRAV/TRDV repertoire has involved a particularly complex series of duplication events. In direct contrast, the majority of the murine TRAV/TRDV expansion is accounted for by a recent single duplication event in which a ~410 Kb segment containing 40 TRAV/TRDV genes was replicated, whilst in the human TRA/TRD locus homology unit duplication has not been documented. Similarly, homology unit replication in other mammalian TR loci is much simpler than that described here. Due to the fragmented nature of the TRA/TRD locus in the UMD3.1 assembly, many of the homology unit copies analysed were incomplete and, with the exception of homology unit 2 (TRAV2-TRAV3), it was not possible to define the full extent of the homology units. Therefore, although our data clearly demonstrate the key role homology unit duplication has had in evolution of the bovine TRAV/TRDV repertoire, analysis of improved, more contiguous assemblies of the bovine TRA/TRD locus will be required to fully appreciate the extent and complexity of homology unit duplication.

In addition to a prolific ‘birth’ rate, bovine TRAV/TRDV genes also exhibit a high ‘death’ rate (37.2% pseudogenes) compared to humans (19%) and mice (16.5%). There was marked variation in the percentages of non-functional genes between the multi-membered subgroups; for example amongst the 6 largest subgroups it ranged from 83.3% (TRAV23) to 20% (TRDV1). Although observed in IG V subgroups [32], the high proportion of pseudogenes seen in several of the bovine TRAV/TRDV subgroups, is rare in TR V subgroups. Based on the assumption that within the context of ‘birth-and-death’ evolution, homology unit replication is utilised to expand the functional TRAV/TRDV gene repertoire, the frequent co-localisation within homology units of genes from low (e.g. TRDV1 or TRAVX) and high (TRAV23 or TRAV18) ‘death’ rate subgroups, suggests that expansion of some high ‘death’ rate TRAV/TRDV subgroups may have occurred as a ‘collateral’ consequence of selection for juxtaposed low ‘death’ rate subgroups. Such ‘collateral’ expansion of V gene repertoires appears to be a feature of the evolution of other bovine IG/TR loci. Genomic analysis of the bovine IGLH locus suggests that the non-functional IGHV2 subgroup may have been expanded due to co-localisation of IGHV2 and functional IGHV1 genes in a duplicated homology unit [33] and similarly in the IGL locus many members of the expanded but predominantly non-functional IGLV5 subgroup are found juxtaposed to functional IGLV1 subgroup 1 genes [34]. Many bovine TRAV/TRDV pseudogenes in equivalent/analogous locations within different replicates of a homology unit share a common mutational lesion (e.g. TRAV8 genes in replicates of homology unit 9 all have an identical 4 bp deletion in the V exon) whilst conversely there are examples of analogous genes in homology unit replicates being a mixture of pseudogenes with non-identical lesions and functional genes (e.g. TRAV24 genes in homology unit 9). This indicates that ‘collaterally’ expanded genes may either be pseudogenes prior to duplication or may, due to absence of selection, lose functionality following replication. Notably, TRDV1 genes are co-located in 4 different homology units with different permutations of genes from 4 of the other massively expanded subgroups - TRAV22, 23, 25 and 26. Whilst the percentage of TRDV1 pseudogenes (20%) is much lower than the bovine TRAV/TRDV average (37.2%) that of the other subgroups is higher, ranging from 43.4-83.5%. Thus, under the model of ‘collateral’ expansion, replication of homology units 7–10 and the consequent expansion of subgroups TRAV22, 23, 25 and 26 may have largely been due to evolutionary selection for a large functional TRDV1 repertoire. As these 5 subgroups together account for nearly half of the annotated bovine TRAV/TRDV genes, TRDV1 selection may have been a critical force in the evolution of the bovine TRAV/TRDV repertoire.

A unique feature of the TRAV/TRDV locus is the co-localisation and sharing of V genes available for rearrangement of 2 distinct antigen-specific receptor chains expressed by 2 distinct lineages of lymphocytes. Consequently, to understand the evolution of the bovine TRAV/TRDV gene repertoire in the context of the TR expressed by αβ and γδ T cells we undertook an analysis of the TRA and TRD transcripts expressed by these T cell subsets. Phylogenically defined TRAV/TRDV gene orthologues don’t necessarily have the same capacity to partake in TRA and TRD rearrangements – for example mTRDV3 and hTRAV40 are phylogenetic orthologues but rearrange exclusively with TRDC and TRAC respectively. It was therefore necessary to first empirically evaluate TRAV/TRDV subgroup usage in rearranged TRA and TRD transcripts using TRAV/TRDV subgroup-specific and TRAC/TRDC specific primers. This analysis confirmed that each of the subgroups assigned as TRAV based on orthology to human TRAV subgroups were represented in rearranged TRA chain transcripts (with the exception of TRAV39) and that genes from all of the subgroups assigned as TRDV were demonstrated to be represented in TRD chain transcripts. Only genes from subgroups TRAV33, TRDV1, TRDV3 and TRDVb3 were expressed in both rearranged TRA and TRD chains, extending the previous observations of dual usage of these subgroups [17]. Thus, in contrast to humans and mice, dual usage of TRAV/TRDV genes in cattle makes a minimal contribution to diversifying the available TRDV germline repertoire but instead has been employed to further diversify the germline repertoire of V genes available for TRA rearrangement.

Commensurate with the number of TRAV/TRDV subgroups available for rearrangement, ‘unbiased’ transcript analysis demonstrated that the TRA repertoire uses genes from a diverse range of TRAV/TRDV subgroups, most of which are represented at <5%, whereas the TRD repertoire predominantly expressed TRDV1 genes, with minor contributions from a limited number of additional TRAV/TRDV subgroups. The dominance of TRDV1 in the expressed TRD repertoire suggests a functional advantage conferred by TRD chains utilising TRDV1 genes may have selected for the expansion of the TRDV1 genomic repertoire and consequently been a major determinant in the replication of homology units 7–10. Conversely the under-representation of TRAV26 (and TRDV1) genes in the expressed TRA repertoire suggests functional selection through TRA chain expression has not been a primary factor in replication of these homology units, but rather supports the concept that some TRAV subgroups have been expanded ‘collaterally’ as a consequence of being within the same homology units as TRDV1. However, over-representation of TRAV3 and TRAVX gene usage in the expressed TRA repertoire indicates that TRA selection has driven replication of other homology units (2, 5 and 6). As such the transcript data indicate that TRA and TRD selection may have driven expansion of different components of the genomic bovine TRAV/TRDV gene repertoire, with TRD selection playing an apparently disproportionately large role. The significance of TRD selection in shaping evolutionary development of the functional bovine TRAV/TRDV genomic repertoire is suggested by the marked increase in the proportion of Group 4 genes (preferential used in TRD chains) and concomitant decrease in the proportion of Group 1 genes (preferentially used by TRA chains) seen in cattle compared to mice and humans.

Although our studies have provided an insight into the potential mechanisms of the evolution of the bovine TRA/TRD locus, the biological factors that have led to the massive expansion of the TRAV/TRDV repertoire remain a matter of conjecture. Cattle, along with other artiodactyl species have proportionally higher numbers of circulating γδ T cells than humans and mice; this is assumed to reflect a greater immunological role for γδ T cells in these species [35]. A parallel but apparently evolutionarily independent expansion of the TRDV1 subgroup in pigs (*Sus scrofa*) may signify that in artiodactyls common selective forces have led to high frequencies of γδ T cells and TRDV1 gene duplication. Incomplete knowledge on the functional roles of γδ T cells and the ligands recognised by γδ TR limit our ability to construct a hypothesis of the evolutionary advantage conferred to artiodactyls by the expansion of TRDV1 genes. However, it is well recognised in mice and humans that subsets of γδ T cells defined by specific TRDV (and TRGV) gene expression have distinct functional phenotypes (reviewed in [36]). In addition to roles such as cytotoxicity and IFNγ production, human TRDV1^+^ γδ T cells have been shown to express an immune-regulatory phenotype following stimulation with a TR-independent agonist [37]. Recent evidence demonstrating that γδ T cells rather than FoxP3^+^CD4^+^T cells contain the dominant regulatory T cell subset in cattle [38] raises the intriguing possibility that the requirement for a substantial immune-regulatory T cell population may have been a factor leading to the expansion of TRDV1^+^ γδ T cells in cattle and other artiodactyls.

## Conclusion

A comprehensive understanding of the evolutionary development of complex multi-gene families, such as the V genes of IG/TR loci requires integration of a variety of data. Previous studies cataloguing the bovine TRAV/TRDV genes have demonstrated the unprecedented size of this repertoire but have not addressed the underlying evolutionary processes leading to its expansion. By combining data on gene characterisation, function, organisation and expression we have shown that the expansion of bovine TRAV/TRDV genes has been achieved primarily through a highly complex series of homology unit replications. We provide evidence indicating that as a consequence of this mode of replication a substantial proportion of the expanded TRAV gene repertoire may have occurred ‘collaterally’ as a consequence of positive selection for duplication of adjacent TRAV/TRDV segments, most notably TRDV1 genes. As TRD selection appears to be the main driver of TRDV1 expansion, this suggests that in bovine (and perhaps other artiodactyls) TRD selection may have been a dominant factor in the genomic expansion of a number of TRAV subgroups. Such data provide an important novel perspective of the evolutionary dynamics that have led to the development of the dramatically expanded bovine TRAV/TRDV repertoire and may have relevance to other IG/TR loci with enlarged V gene repertoires.

## Methods

### Genome and sequence analysis

The UMD3.1 genome assembly sequence was accessed through the ensembl website (http://www.ensembl.org). To identify bovine TRA/TRD gene segments, a series of BLASTn searches using the sequences of all human and murine TRAV/TRDV, TRDD, TRDJ, TRAJ, TRAC and TRDC genes (downloaded from the IMGT website (http://www.imgt.org)) and previously published bovine TRDV and TRDD sequences [17, 21] were completed. Coordinates given in the gene annotation (Additional file 1) include the L exon, intron, V exon and RS of TRAV/TRDV genes, the splice site, RS and coding regions of the TRAJ, TRDJ and TRDD genes and the 4 exons of the TRAC and TRDC genes (including the polyA signal sequence). Basic sequence analysis, such as CLUSTALw alignments [39] and translations were conducted using the DNAsis Max v2.7 programme (MiraiBio, Almeda, CA, USA). *In silico* analysis of gene function considered the following parameters: i) presence of splice sites appropriate for RNA editing, ii) open reading frames which included codons for the conserved cysteine, tryptophan and cysteine residues at positions 23, 41 and 104 of TRAV/TRDV genes (IMGT unique numbering system [40]) and the canonical FGxG motif in TRAJ and TRDJ genes, iii) presence of a 23- and/or 12-RS sequence compatible with effective recombination [41, 42], and iv) location either on Chromosome 10 or an unassigned contig/scaffold. Nucleotide identity analysis was performed using the Pipmaker and Multi-pipmaker programmes [43]. EST sequence data was derived from megablast searches of the NCBI *Bos taurus* EST trace archive (http://blast.ncbi.nlm.nih.gov) conducted with the nucleotide sequences of exon 1 of the bovine TRAC and TRDC genes in March 2013.

### Phylogenetic analyses

Phylogenetic analysis was performed on the nucleotide sequence of the V-REGION (IMGT numbering 1–108) of TRAV/TRDV genes and the coding sequence of TRAJ and TRDJ genes of humans, mice and bovine (as identified in UMD3.1). Neighbour-joining method, minimum evolution and UPMGA analyses were performed with the MEGA5 software package [44] using the uncorrected nucleotide differences (p-distance), which provides better results when examining a large number of sequences which contain a relatively small number of nucleotides [45].

### cDNA analysis of rearranged TRA and TRD transcripts

Blood from Holstein-Friesian animals was collected by jugular venu-puncture into EDTA and PBMC isolated by density gradient centrifugation over Ficoll-paque Plus (GE Healthcare, Little Chlafont, Bucks., UK) according to the manufacturer’s instructions. αβ and γδ T cell populations were obtained by co-staining PBMC with monoclonal antibodies against bovine γδ TR (GB21A - IgG2b isotype) and CD3 (MM1A - IgG1 isotype), both from VWRD, Pullman, WA, USA and then appropriate conjugated isotype-specific secondary antibodies (Molecular Probes, Invitrogen, Paisley, UK) prior to sorting into CD3^+^ γδ^+^ (γδ T cell) and CD3^+^ γδ^-^ (αβ T cell) populations on a FACSaria cell sorter (BD Biosciences, Oxford, UK). All animals used in this study were part of the Langhill Herd at the University of Edinburgh Farm. The work was approved by The Roslin Institute Animal Welfare and Ethical Review Body and conducted under license and in accordance with the UK government Animal (Scientific Procedures) Act 1986, UK.

For the TRAV/TRDV subgroup-specific PCR reactions total RNA was extracted using Tri-reagent (Sigma–Aldrich, Poole, Dorset, UK) and cDNA synthesised using the Reverse Transcription System (Promega, Madison, WI, USA) with priming by the Oligo (dT)_15_ primer; both kits were used according to the manufacturer’s instructions. Panels of PCRs were then conducted with each reaction using a single TRAV/TRDV subgroup-specific 5′ primer (Table 5) in combination with either a TRAC-(TRAC1 -GGGCTTCTCAGCTGGTACAC) or TRDC-(TRDC1 - CCCAGGTGAGATGGCAATAG) specific 3′ primer. Individual reactions were composed of 1 μl cDNA template, 10 pmol each of the relevant 5′ and 3′ primers, 0.5 units of Biotaq (Bioline, London, UK) and 2 μl SM-0005 10× buffer (ABgene, Epsom, Surrey, UK) per 20 μl reaction. Cycling conditions were 5 min. at 95°C, 35 cycles of (1 min. at 95°C, 1 min. at 60°C, 1 min. at 72°C), and a final extension period of 5 min. at 72°C.

**Table 5:** **Sequence of TRAV/TRDV subgroup-specific primers**

TRAV/TRDV subgroup	Subgroup-specific 5′ primer sequence
AV1	CAGGAAAAGGCGTTAAGCAG
AV2	GGTCTCTTTGGAGGGAGCTG
AV3	CAGCCAGAAGCTGAGGTC
AV4	AGTGACCGTGCTCCTGAC
AV8.1	TGATGCTTGAGATGCTCCTG
AV8.2	CGGTCACATCAACGTCTCTG
AV8.3	ATTCCAGAGGCCAGTCAGTG
AV9	CTTCTCCAGGCTTAGTGACTG
AV10	TGAGTGGCAAAAACCAAGTG
AV12	CACAGTGGAGCAGAGTCCTG
AV5 and 13	CTTGTGGCT(A/G)CAGCTGGAC
AV14	AGGTGGTCGTGGCTTCACT
AV16	CAAGAGCCCAGACAGTGACTC
AV17	GAGAAGCTTCTGGCCCTG
AV18	GTTGTTACCCT(C/T)CCCGAGAAG
AV19	G(G/C)ATGTA(A/G)CCTTGAACTGTGC
AV20	CCAGGAGGGGGACAGTCT
AV21	GCCTGCTCATCCTTTGGTTA
AV22	CTT(C/T)TGTTTGCCCAGGTTTG
AV23	AAGTGACCAA(G/C)AGCAGGTGA
AV24	CCCTTGCTGTGGGTTCAG
AV25	G(G/C)ACCAGTGTTGATCTTA(C/T)GGA
AV26	TCCATGGATT(A/G)T(G/C)CTGAAGG
AV27	TGGTCTTTTGGATTCAACTGG
AV28	ACAAAGAAGAGTCTTGCTGAGTC
AV29	GCTTCAGTCTGACTGGGTG
AV33	GGCTGACAAAGTTACTGAAGC
AV35	GACATGTGTGAGTGCCCAAC
AV36	CCCCATCTCTGATTGTCCAT
AV38	CACAGTGACCCTGGACTGTA
AV41	AGGAAGGAGACCTCGTCACA
AVX	GGCTCTC(A/C)TGACCCTGAACT
AVY	GGTGGAGCAGA(A/G)TCCTTCAG
DV1.1	(G/C/T)C(A/T)GCTCTGGGTG(C/T)TCCT
DV1.2	GTGGCCCAGA(A/G)(A/C/T)GTTACTCA
DV2	TCATCCACCTCACCCTCTTC
DVY	CGTAACTGGAGGGAACTGGA
DV3	ATGTTTCTCCCTGTGGGCTTC
DVb3	TCTGCCTTGGTTCCAACAAT

All of these primers were designed as part of this study based on sequences of functional members of the TRAV/TRDV subgroups identified in the UMD3.1 genome assembly, with the exception of DV3 which was published previously [17].

For the SMART PCR mRNA was extracted using the Dynabeads mRNA Direct Kit (Life Technologies, Paisley, UK) and cDNA synthesised with the SMARTer RACE cDNA Kit (Clontech, Paris, France) using TRAC- (TRAC2 - TGTATTGGCATCCAGCATCG) and TRDC- (TRDC2 - CGAGGTTTGTCCCATTTTTC) specific primers. PCR using the Phusion High-Fidelity PCR kit (New England BioLabs, Hitchin, UK) was then completed with the TRAC1/TRDC1 and the Long and Short UPM primers. Reactions were composed of Phusion HF amplification buffer, 3% DMSO, 0.2 mM dNTPs, 0.1 μM Long UPM, 0.5 μM Short UPM, 0.5 μM of internal TRAC or TRDC primer, Phusion Hot Start DNA polymerase (2 U/100 μl final reaction volume) and cDNA (1 μl/100 ul final reaction volume). Cycling conditions were 30 s at 98°C, 30 cycles of (98°C for 10 s, 57°C for 20 s, 72°C for 20 s), and a final extension period of 5 min. at 72°C.

PCR products were visualised in 1.5% TAE-agarose gels, purified using Qiaquick gel extraction kit (Qiagen, Crawley, UK) and cloned into pGEM-T Easy (Promega, Madison, WI, USA). Selected cloned products were sequenced (Genepool, University of Edinburgh, UK) to permit verification of the sequence of the amplified TRA/TRD chains. One hundred and twenty-six partial TRA and TRD transcripts, including representatives from each of the expressed functional TRAV/TRDV subgroups have been submitted to Genbank (accession numbers JX065635-JX065739 and JX101710-101720).

### Availability of supporting data

The nucleotide and phylogenetic datasets supporting the results of this article are available in GenBank (http://www.ncbi.nlm.nih.gov/ - accession numbers JX065635-JX065739 and JX101710-101720) and Dryad (http://doi.org/10.5061/dryad.h8j92) respectively. The primary sequence data, as derived from UMD3.1 assembly of the bovine genome and accessed through ensembl (http://www.ensembl.org/index.html) is in Additional file 5.

## Nomenclature

Without a fully assembled TRA/TRD locus it was not possible to implement the approved nomenclature system which requires knowledge of the genomic order of genes from the 5′ to the 3′ end of the locus [46]. Bovine TRAV/TRDV subgroups have been named according to the orthologous human subgroups where appropriate and an alphabetic designation where this was not possible. Members in the V gene subgroups have been given alphabetic rather than numeric designations, similar to systems employed in previous work [8, 14], although there is no correspondence between the TRDV1 nomenclature employed here and in [14]. The TRDD and TRDJ nomenclature is consistent with previously published work and TRAJ genes have been assigned numbers according to their 5′ to 3′ order in the genome.

## Electronic supplementary material

Additional file 1: **Location of TRA/TRD genes and regulatory elements in UMD3.1.** For each gene the name, type of gene segment, gene orientation (ori.), chromosomal location, start and stop co-ordinates, chromosome orientation (chrom. ori.), contig number and predicted functional competency are shown. Sequences matching TRA/TRD genes marked by an asterisk (as a suffix to the gene name) have been described in previous annotations - Reinink and Van Rhijn [15] and Herzig et al. [14], or in the IMGT database (July 2014 – based on data from Herzig et al. [14]). The coloured blocks represent the locations of homology units (see Additional file 8). (PDF 2 MB)

Additional file 2: **Neighbour-joining phylogenetic tree of all murine, human and bovine (from the UMD3.1 assembly) TRAV/TRDV genes.** Analysis of the nucleotide sequence of the V-REGION (IMGT nomenclature) following pairwise deletion to remove gaps in the alignment. The final dataset included 400 positions. The sequence of bTRBV3a was used to root the tree. Based on a 1000 boot strap replicates the phylogenetically inferred orthologous TRAV/TRDV subgroups were supported by percentage bootstrap values (P_B_) of >90% in all cases except for bTRAV38 (P_B_ = 83%), bTRAV5 (P_B_ = 59%), bTRAV13 (P_B_ = 85%) and bTRAV8 (P_B_ = 68%). Sequence identity between bovine and human genes in orthologous groups ranged from 63.1-84.9%, sufficient to assign them as inter-species orthologues [47]. Generally the phylogenetically defined bovine TRAV/TRDV subgroups adhered to the convention of members sharing >75% nucleotide identity [[25], [26]]. However, within both the bTRDV1 and bTRAV8 subgroups identity between some members was <75% (down to 68.0 and 69.7% respectively) and conversely the identity between some bTRAV5/13 and some bTRAVX/18 members was >75%. Due to difficulties in alignment the following genes were excluded from the analysis i) bovine genes for which only incomplete or partial genomic sequences were available, ii) bTRAV11a – due to the presence of a large insert and iii) mTRAV15-3, mTRAV15D-3, hTRAV8.5. h = human, b = bovine and m = murine. (PDF 912 KB)

Additional file 3: **Comparison of the annotated TRA/TRD genes with previously published annotations of the bovine TRA/TRD locus and data available in the IMGT website (July 2014).** Summary of comparisons with the TRA/TRD gene repertoires identified by Reinink and Van Rhijn [15], Herzig et al. [14] and present in the IMGT database (July 2014). Also summarised are the number of TRA/TRD genes which have been identified in previous annotation studies. (PDF 411 KB)

Additional file 4: **Annotation of the exons and RS sequences of the TRA/TRD genes.** The coordinates of the (A) L-exons, V-exons and RS of each TRAV/TRDV gene, (B and C) RS and J-gene of each TRAJ and TRDJ gene, (D) RS and D-genes of each TRDD gene and (E) exons of the TRAC and TRDC gene are detailed. (PDF 2 MB)

Additional file 5: **Sequences of (A) Chr10: 22100001–25700000, (B) Chr10:60200001–60300000, (C) Chr9:71300001–71400000 and (D) Contig DAAA0206600.** This provides the primary sequence resources from which the sequences of the TRA/TRD genes annotated can be extracted. (TXT 4 MB)

Additional file 6: **Neighbour-joining phylogenetic tree of all murine, human and bovine (from the UMD3.1 assembly) TRAJ genes.** Analysis of the nucleotide sequence of the coding domain of TRAJ genes following pairwise deletion to remove gaps in the alignment. The final dataset had a total of 85 positions. Based on a 1000 boot strap replicates the orthologous TRAJ genes from mouse, human and cattle (where all genes were functional) formed phylogenetic groups supported by percentage bootstrap values (P_B_) of >75% (with the exception of TRAJ6 (P_B_ =54%), 9 (53%) and 48 (67%)). P_B_ values supporting the phylogenetic groups of TRAJ orthologues which included non-functional members were generally high but in several cases were <50%. h = human, b = bovine and m = murine. The formation of ‘triads’ composed of single genes from each species (except TRAJ10 which lacks a murine gene and TRAJ8 which contains 2 bovine genes) that have the same relative position in the genome (as denoted by their numerical designation) demonstrates conserved synteny. The level of nucleotide identity between orthologous bovine and human TRAJ genes ranges from 63.2% to 95.2%. (PDF 14 KB)

Additional file 7: **Sequence alignment of regulatory elements in the 3′ end of the bovine (from the UMD3.1 assembly), murine and human TRA/TRD locus.** Sequences of defined transcription factor binding sites are shown in grey highlight. Nucleotide identity between orthologous sequences is shown by dashes and gaps by dots. (A) Alignment of the human, murine and putative bovine Eα sequences. Protein binding regions in the core human Eα (Tα1-4) are indicated by arrows. The 300 bp sequence of the putative bovine Eα shares 87.5% and 78.2% nucleotide identity with the human and murine Eα. Within the core Tα1-Tα2 fragment of the Eα, which constitutes the DNA scaffold for the generation of the nucleo-protein structure termed the ‘enhanceosome’ that is critical for Eα function [[48–50]], the CREB, TCF-1/LEF-1 and Ets binding sites show absolute conservation between the bovine, human and murine sequences. Simultaneous occupancy of these sites is a minimal requirement for Eα activity. Numerous other transcription factor binding sites which have been shown to be occupied in the Eα and play a role in appropriate regulation of Eα function [[51–53]] are also conserved in the putative bovine Eα sequence. (B) Alignment of the human, murine and putative bovine Eδ sequences. Protein binding regions in the core human Eδ (Sϵ3 and Sϵ4) are indicated by arrows. The 70 bp sequence spanning the essential Sϵ3 and Sϵ4 core of the enhancer [54] shows 77.1% and 72.8% nucleotide identity to the corresponding human and murine sequences. The CBF/PEPB2 and c-myb binding sites within Sϵ3 which are critical for the formation and function of the Eδ ‘enhanceosome’ [55] are conserved in the bovine sequence as are 2 GATA-3 binding sequence in Sϵ4. (PDF 165 KB)

Additional file 8: **A –TRAV/TRDV homology units identified within UMD3.1.** Details of the replicons of the 10 putative homology units are given. Each homology unit has been ascribed a colour code that can be used to examine the position of homology units within Additional file 1. Each copy of the homology unit has been designated a number (e.g. Homology Unit 1.1) to enable correspondence with the nucleotide identity analysis presented in Additional file 9. For each homology unit the TRAV/TRDV subgroup motif is shown (TRAV subgroups unless otherwise specified) and for each homology unit copy the specific genes and their functionality (F – functional, PS – pseudogene, I – incomplete) are detailed. Where present on the same contig, genes flanking the putative homology unit replicates are shown on a green background. However, if homology unit copies appear to extend beyond the limits of the contig on which they’re located this is shown as END on a red background. Three sequences show high identity with both homology unit 5 and 6 – these are designated as ambiguous (AMB in Additional file 1). Genes within homology unit 5 replicates shown on an orange background may represent a variant of the homology unit that has arisen during different replicative iterations. Dashes within homology unit replicant gene sequences represent absent genes - most likely as consequences of post-replication insertion/deletion. Non-contiguous sequences have generally not been used for this analysis, but in circumstances where joining non-contiguous sequences rationalised the number of fragments of homology units identified, exceptions have been made (genes flanking breaks in contiguous sequence are shown in red italicised script on a yellow background). Homology unit replicates identified by an asterisk were used as reference sequences for nucleotide identity analysis using the Pipmaker/Multipipmaker programmes; boxed subgroups in TRAV/TRDV subgroup homology unit motifs are absent from the reference sequences. (XLSX 18 KB)

Additional file 9: **B – Nucleotide identity analysis of putative homology units.** Nucleotide identity analysis was conducted using the Pipmaker and Multipipmaker programmes as described in Materials and Methods. For homology units with only 2 replicates (homology units 1, 3 and 10) dotplots are shown and for homology units with >2 replicates (homology units 4, 5, 6, 7, 8 and 9) a summary of the Multipip output is shown. For homology unit 2 a dotplot covering the entire region of alternating TRAV2 and TRAV3 genes against itself is shown as this best exemplifies the multiple tandem repeats that have occurred. In dotplots diagonal lines represent areas of nucleotide identity; in the Multipip output areas of high nucleotide identity are represented by red colouring. (PDF 356 KB)

Additional file 10: **A Genomic sequence of the bovine TRAJ genes identified in UMD3.1.** The table shows the (i) RS sequence, (ii) reading frame of the coding sequence, (iii) nucleotide and predicted amino acid sequences of the coding sequence and (iv) the sequence of the 3′ splice site. The canonical FGxG motif of the TRAJ sequence is shown with yellow highlighting. TRAJ genes that are predicted to be pseudogenes are shown with their name and the identified defect highlighted in red. B - Summary of non-functional TRAJ gene segments in UMD3.1. Summary of the lesions identified in TRAJ genes that are considered to render them non-functional. (PDF 288 KB)

Additional file 11: **Summary of non-functional TRAV/TRDV gene segments in UMD3.1.** The lesions identified in TRAV/TRDV genes which are considered to render them non-functional are summarised. (PDF 584 KB)

## Abbreviations

Ig: Immunolobulin
IMGT: IMGT®, the international ImMunoGeneTics information system®
RS: Recombination signal sequence
TR: T cell receptor
TRA: T cell receptor alpha
TRD: T cell receptor delta
V: Variable gene segment
J: Joining gene segment
D: Diversity gene segment
C: Constant gene segment.
