# Supplementary material for: Genomic analysis offers insights into the evolution of the bovine TRA/TRD locus
Source: BMC Genomics. 2014 Nov 19;15(1):994. doi: 10.1186/1471-2164-15-994 (PMC4289303; doi:10.1186/1471-2164-15-994)
Supplement: Supplementary file 1 — Additional file 1: Location of TRA/TRD genes and regulatory elements in UMD3.1. For each gene the name, type of gene segment, gene orientation (ori.), chromosomal location, start and stop co-ordinates, chromosome orientation (chrom. ori.), contig number and predicted functional competency are shown. Sequences matching TRA/TRD genes marked by an asterisk (as a suffix to the gene name) have been described in previous annotations - Reinink and Van Rhijn [15] and Herzig et al. [14], or in the IMGT database (July 2014 – based on data from Herzig et al. [14]). The coloured blocks represent the locations of homology units (see Additional file 8). (PDF 2 MB) [file 12864_2014_6826_MOESM1_ESM.pdf]

	Previous annotations											
Name	Reinink and Van Rhijn (2009)	Herzig et al. (2010)/IMGT	Gene group	Gene orientation	Chromosome	Start	Stop	Chromosome orientation	Contig	Functional	Homology unit	Homology Unit Overlap
E alpha			Regulatory element	-	Chr:10	22102864	22103163	+	DAAA02028052.1	-		
TRAC*		TRAC	TRAC	-	Chr:10	22106925	22111855	+	DAAA02028052.1	Functional		
bTRAJ1*		TRAJ1	TRAJ	-	Chr:10	22115247	22115341	+	DAAA02028052.1	Functional		
bTRAJ2*		TRAJ2	TRAJ	-	Chr:10	22116214	22116312	+	DAAA02028052.1	Functional		
bTRAJ3*		TRAJ3	TRAJ	-	Chr:10	22116760	22116851	+	DAAA02028052.1	Functional		
CSB			Regulatory element	-	Chr:10	22117370	22117493	+	DAAA02028052.1	-		
bTRAJ4*		TRAJ4	TRAJ	-	Chr:10	22117730	22117813	+	DAAA02028052.1	Pseudogene		
bTRAJ5*		TRAJ5	TRAJ	-	Chr:10	22120919	22121011	+	DAAA02028052.1	Functional		
bTRAJ6			TRAJ	-	Chr:10	22122107	22122201	+	DAAA02028052.1	Functional		
bTRAJ7*		TRAJ6	TRAJ	-	Chr:10	22122688	22122776	+	DAAA02028052.1	Functional		
bTRAJ8-1*		TRAJ7	TRAJ	-	Chr:10	22124233	22124325	+	DAAA02028052.1	Functional		
bTRAJ9			TRAJ	-	Chr:10	22124822	22124915	+	DAAA02028052.1	Functional		
bTRAJ8-2*		TRAJ8	TRAJ	-	Chr:10	22127021	22127113	+	DAAA02028052.1	Functional		
bTRAJ10*		TRAJ9	TRAJ	-	Chr:10	22127480	22127575	+	DAAA02028052.1	Functional		
bTRAJ11*		TRAJ10	TRAJ	-	Chr:10	22128491	22128583	+	DAAA02028052.1	Functional		
bTRAJ12*		TRAJ11	TRAJ	-	Chr:10	22129052	22129144	+	DAAA02028052.1	Functional		
bTRAJ13			TRAJ	-	Chr:10	22129908	22130003	+	DAAA02028052.1	Pseudogene		
bTRAJ14*		TRAJ12	TRAJ	-	Chr:10	22130630	22130714	+	DAAA02028052.1	ORF		
bTRAJ15			TRAJ	-	Chr:10	22131522	22131614	+	DAAA02028052.1	Functional		
bTRAJ16*		TRAJ13	TRAJ	-	Chr:10	22132617	22132710	+	DAAA02028052.1	Functional		
bTRAJ17*		TRAJ14	TRAJ	-	Chr:10	22134234	22134329	+	DAAA02028052.1	Functional		
bTRAJ18*		TRAJ15	TRAJ	-	Chr:10	22135341	22135439	+	DAAA02028052.1	Functional		
bTRAJ19			TRAJ	-	Chr:10	22135733	22135825	+	DAAA02028052.1	Pseudogene		
bTRAJ20*		TRAJ16	TRAJ	-	Chr:10	22136702	22136792	+	DAAA02028052.1	Functional		

bTRAJ21*		TRAJ17	TRAJ	-	Chr:10	22137413	22137500	+	DAAA02028052.1	Functional		
bTRAJ22*		TRAJ18	TRAJ	-	Chr:10	22138937	22139032	+	DAAA02028052.1	Functional		
bTRAJ23*		TRAJ19	TRAJ	-	Chr:10	22140056	22140151	+	DAAA02028052.1	Functional		
bTRAJ24*		TRAJ20	TRAJ	-	Chr:10	22140497	22140592	+	DAAA02028052.1	Functional		
bTRAJ25*		TRAJ21	TRAJ	-	Chr:10	22141352	22141444	+	DAAA02028052.1	Functional		
bTRAJ26			TRAJ	-	Chr:10	22141650	22141742	+	DAAA02028052.1	ORF		
bTRAJ27*		TRAJ22	TRAJ	-	Chr:10	22143818	22143909	+	DAAA02028052.1	Functional		
bTRAJ28*		TRAJ23	TRAJ	-	Chr:10	22144466	22144564	+	DAAA02028052.1	Functional		
bTRAJ29*		TRAJ24	TRAJ	-	Chr:10	22145457	22145549	+	DAAA02028052.1	Functional		
bTRAJ30*		TRAJ25	TRAJ	-	Chr:10	22146520	22146611	+	DAAA02028052.1	Functional		
bTRAJ31*		TRAJ26	TRAJ	-	Chr:10	22148614	22148704	+	DAAA02028052.1	Functional		
bTRAJ32*		TRAJ27	TRAJ	-	Chr:10	22150419	22150517	+	DAAA02028052.1	Functional		
bTRAJ33*		TRAJ28	TRAJ	-	Chr:10	22151163	22151252	+	DAAA02028052.1	ORF		
bTRAJ34*		TRAJ29	TRAJ	-	Chr:10	22151853	22151942	+	DAAA02028052.1	Functional		
bTRAJ35*		TRAJ30	TRAJ	-	Chr:10	22152883	22152971	+	DAAA02028052.1	ORF		
bTRAJ36			TRAJ	-	Chr:10	22154348	22154441	+	DAAA02028052.1	Functional		
bTRAJ37*		TRAJ31	TRAJ	-	Chr:10	22155060	22155154	+	DAAA02028052.1	Functional		
bTRAJ38*		TRAJ32	TRAJ	-	Chr:10	22156562	22156656	+	DAAA02028052.1	Functional		
bTRAJ39*		TRAJ33	TRAJ	-	Chr:10	22157201	22157296	+	DAAA02028052.1	Functional		
bTRAJ40*		TRAJ34	TRAJ	-	Chr:10	22159313	22159402	+	DAAA02028052.1	Functional		
bTRAJ41*		TRAJ35	TRAJ	-	Chr:10	22161011	22161104	+	DAAA02028052.1	Functional		
bTRAJ42*		TRAJ36	TRAJ	-	Chr:10	22161480	22161578	+	DAAA02028052.1	Functional		
bTRAJ43*		TRAJ37	TRAJ	-	Chr:10	22162268	22162357	+	DAAA02028052.1	Functional		
bTRAJ44*		TRAJ38	TRAJ	-	Chr:10	22163570	22163664	+	DAAA02028052.1	Functional		
bTRAJ45*		TRAJ39	TRAJ	-	Chr:10	22164415	22164510	+	DAAA02028052.1	Functional		
bTRAJ46*		TRAJ40	TRAJ	-	Chr:10	22164927	22165022	+	DAAA02028052.1	Functional		
bTRAJ47			TRAJ	-	Chr:10	22165671	22165760	+	DAAA02028052.1	Functional		

bTRAJ48*		TRAJ41	TRAJ	-	Chr:10	22167691	22167786	+	DAAA02028052.1	Functional		
bTRAJ49*		TRAJ42	TRAJ	-	Chr:10	22168581	22168669	+	DAAA02028052.1	Functional		
bTRAJ50*		TRAJ43	TRAJ	-	Chr:10	22169468	22169556	+	DAAA02028052.1	Functional		
bTRAJ51			TRAJ	-	Chr:10	22170836	22170925	+	DAAA02028052.1	Pseudogene		
bTRAJ52*		TRAJ44	TRAJ	-	Chr:10	22171813	22171914	+	DAAA02028052.1	Functional		
bTRAJ53*		TRAJ45	TRAJ	-	Chr:10	22175027	22175125	+	DAAA02028052.1	Functional		
bTRAJ54*		TRAJ46	TRAJ	-	Chr:10	22175681	22175777	+	DAAA02028052.1	Functional		
bTRAJ55			TRAJ	-	Chr:10	22176282	22176375	+	DAAA02028052.1	Pseudogene		
bTRAJ56*		TRAJ47	TRAJ	-	Chr:10	22178437	22178527	+	DAAA02028052.1	Functional		
bTRAJ57*		TRAJ48	TRAJ	-	Chr:10	22179066	22179161	+	DAAA02028052.1	Functional		
bTRAJ58*		TRAJ49	TRAJ	-	Chr:10	22180304	22180399	+	DAAA02028052.1	Functional		
bTRAJ59*		TRAJ50	TRAJ	-	Chr:10	22181469	22181554	+	DAAA02028052.1	Functional		
bTRAJ60*		TRAJ51	TRAJ	-	Chr:10	22181703	22181792	+	DAAA02028052.1	Functional		
bTRAJ61*		TRAJ52	TRAJ	-	Chr:10	22182669	22182762	+	DAAA02028052.1	Pseudo		
TEA			Regulatory element	-	Chr:10	22184384	22184656	+	DAAA02028052.1	-		
DV3-a			DV3	+	Chr:10	22192121	22192742	+	DAAA02028053.1	Functional		
TRDC*		TRDC	TRDC	-	Chr:10	22195089	22198861	+	DAAA02028053.1	Functional		
E delta			Regulatory element	-	Chr:10	22199996	22200065	+	DAAA02028053.1	-		
TRDJ2*		TRDJ3	TRDJ	-	Chr:10	22201461	22201552	+	DAAA02028053.1	Functional		
TRDJ3*		TRDJ2	TRDJ	-	Chr:10	22205232	22205313	+	DAAA02028053.1	Functional		
TRDJ1*		TRDJ1	TRDJ	-	Chr:10	22211580	22211666	+	DAAA02028053.1	Functional		
TRDD5*		TRDD5	TRDD	-	Chr:10	22212579	22212656	+	DAAA02028053.1	Functional		
TRDD4*		TRDD4	TRDD	-	Chr:10	22229689	22229764	+	DAAA02028053.1	Functional		
TRDD3*		TRDD3	TRDD	-	Chr:10	22238139	22238218	+	DAAA02028053.1	Functional		
TRDD2*		TRDD2	TRDD	-	Chr:10	22277223	22277304	+	DAAA02028053.1	Functional		
TRDD1*		TRDD1	TRDD	-	Chr:10	22293080	22293159	+	DAAA02028053.1	Functional		
DV2-a			DV2	-	Chr:10	22323161	22323689	+	DAAA02028054.1	Functional		
DVY-a			DVY	-	Chr:10	22349850	22350390	+	DAAA02028054.1	Functional		

DVb3-a			DVb3	-	Chr:10	22393915	22394470	+	DAAA02028056.1	Functional		
DVb3-b*	BoTRDV3.1	DV3s2-1	DVb3	-	Chr:10	22406746	22407310	+	DAAA02028055.1	Functional		
AV41-a*	Gene 23		AV41	-	Chr:10	22438607	22439182	+	DAAA02028057.1	Functional		
AV39-a*	Gene 24		AV39	-	Chr:10	22459631	22460166	+	DAAA02028059.1	Psuedogene		
AV38-a			AV38	+	Chr:10	22462003	22462640	+	DAAA02028060.1	Functional		
AV28-a			AV28	-	Chr:10	22471556	22472155	+	DAAA02028062.1	Functional		
AV27-a*	Gene 21		AV27	-	Chr:10	22481449	22482036	+	DAAA02028062.1	Functional		
AV26-a			AV26	-	Chr:10	22499142	22499768	+	DAAA02028062.1	Incomplete		
AV26-b*	Gene 250/28		AV26	-	Chr:10	22504953	22505905	+	DAAA02028063.1	Functional		
AV25-a			AV25	-	Chr:10	22512779	22513264	+	DAAA02028064.1	Incomplete		
AV24-a*	Gene 36		AV24	-	Chr:10	22523574	22524120	+	DAAA02028064.1	Psuedogene		
DV1-a*		DV1s39-1	DV1	-	Chr:10	22529611	22530111	+	DAAA02028064.1	Functional		
AV26-c			AV26	-	Chr:10	22541380	22542514	+	DAAA02028065.1	Incomplete		
AV23-a			AV23	-	Chr:10	22551389	22551927	+	DAAA02028065.1	Psuedogene		
AV22-a*	Gene 40		AV22	-	Chr:10	22552579	22553139	+	DAAA02028065.1	Psuedogene		
AV26-d*	Gene 33		AV26	-	Chr:10	22555593	22556403	+	DAAA02028065.1	Psuedogene		
DV1-b*	BoTRDV1.23	DV1s19-1	DV1	-	Chr:10	22570094	22570696	+	DAAA02028068.1	Functional		
AV25-b			AV25	-	Chr:10	22576442	22577074	+	DAAA02028067.1	Functional		
AV19-a			AV19	-	Chr:10	22585558	22586191	+	DAAA02028067.1	Functional		
AVX-a			AVX	-	Chr:10	22587737	22588486	+	DAAA02028067.1	Psuedogene		
AV25-c			AV25	-	Chr:10	22608673	22609302	+	DAAA02028068.1	Psuedogene		
DV1-c*	Gene 227	DV1s48-1	DV1	-	Chr:10	22619112	22619719	+	DAAA02028069.1	Functional		
AV22-b			AV22	-	Chr:10	22631613	22632173	+	DAAA02028069.1	Functional		
AV8-a			AV8	-	Chr:10	22632546	22633025	+	DAAA02028069.1	Psuedogene		
DV1-d*		DV1s47-2	DV1	-	Chr:10	22643560	22643998	+	DAAA02028069.1	Psuedogene		
AV22-c*	Gene 223		AV22	-	Chr:10	22647491	22648230	+	DAAA02028069.1	Psuedogene		
AV8-b*	Gene 255		AV8	-	Chr:10	22648615	22649084	+	DAAA02028069.1	Psuedogene		
DV1-e*	Gene 30	DV1s42-1	DV1	-	Chr:10	22662964	22663571	+	DAAA02028070.1	Functional		
AV22-d*	Gene 31		AV22	-	Chr:10	22674452	22675008	+	DAAA02028070.1	Functional		

AV8-c*	Gene 26		AV8	-	Chr:10	22675380	22675860	+	DAAA02028070.1	Psuedogene		
DV1-f*	Gene 32	DV1s43-1	DV1	-	Chr:10	22686069	22686679	+	DAAA02028070.1	Functional		
AV26-e*	Gene 41		AV26	-	Chr:10	22695755	22696851	+	DAAA02028070.1	Functional		
DV1-g*	Gene 348	DV1s44-1	DV1	-	Chr:10	22702952	22703546	+	DAAA02028070.1	Functional		
AVX-b			AVX	-	Chr:10	22719014	22719525	+	DAAA02028071.1	Functional	AMB	
AV18-a			AV18	-	Chr:10	22723554	22724066	+	DAAA02028071.1	Psuedogene	AMB	
AVY-a			AVY	-	Chr:10	22738801	22739369	+	DAAA02028072.1	Functional		
AV8-d			AV8	-	Chr:10	22742070	22742574	+	DAAA02028072.1	Psuedogene		
AV22-e			AV22	-	Chr:10	22754056	22754411	+	DAAA02028074.1	Psuedogene		
AV8-e			AV8	-	Chr:10	22754996	22755476	+	DAAA02028074.1	Psuedogene		
DV1-h			DV1	-	Chr:10	22766038	22766636	+	DAAA02028075.1	Functional		
AV26-f			AV26	-	Chr:10	22776924	22777730	+	DAAA02028078.1	Psuedogene		
AV25-d			AV25	-	Chr:10	22794678	22795308	+	DAAA02028082.1	Psuedogene		
AV24-b			AV24	-	Chr:10	22803195	22803523	+	DAAA02028082.1	Incomplete		
DV1-i*	Gene 1		DV1	-	Chr:10	22808623	22809057	+	DAAA02028083.1	Incomplete		
AV26-g			AV26	-	Chr:10	22813972	22815090	+	DAAA02028083.1	Psuedogene		
AV23-b			AV23	-	Chr:10	22820134	22820677	+	DAAA02028083.1	Psuedogene		
AV22-f			AV22	-	Chr:10	22821328	22821865	+	DAAA02028083.1	Psuedogene		
AV26-h*	Gene 91		AV26	-	Chr:10	22827854	22828670	+	DAAA02028084.1	Psuedogene		
DV1-j			DV1	-	Chr:10	22836189	22836789	+	DAAA02028085.1	Functional		
AV25-e			AV25	-	Chr:10	22842847	22843474	+	DAAA02028085.1	Psuedogene		
AV23-c			AV23	-	Chr:10	22844892	22845433	+	DAAA02028085.1	Psuedogene		
AV22-g			AV22	-	Chr:10	22846084	22846644	+	DAAA02028085.1	Psuedogene		
DV1-k*	Gene 3	DV1s33-1	DV1	-	Chr:10	22856348	22856944	+	DAAA02028085.1	Functional		
AV25-f*	Gene 4		AV25	-	Chr:10	22863248	22863869	+	DAAA02028085.1	Psuedogene		
AV26-i			AV26	-	Chr:10	22877059	22878204	+	DAAA02028087.1	Functional		
DV1-l*	BoTRDV1.35	DV1s34-1	DV1	-	Chr:10	22888343	22888919	+	DAAA02028088.1	Functional		
AV23-d*	Gene 6		AV23	-	Chr:10	22893540	22894082	+	DAAA02028088.1	Psuedogene		
AV22-h*	Gene 7		AV22	-	Chr:10	22900141	22900718	+	DAAA02028089.1	Functional		

AV24-c			AV24	-	Chr:10	22905622	22905952	+	DAAA02028089.1	Psuedogene		
DV1-m*	Gene 8	DV1s35-1	DV1	-	Chr:10	22915690	22916297	+	DAAA02028089.1	Functional		
AV23-e			AV23	-	Chr:10	22920423	22921000	+	DAAA02028089.1	Psuedogene		
AV39-b*	Gene 9		AV39	-	Chr:10	22930996	22931528	+	DAAA02028089.1	Functional		
AV38-b*	Gene 11		AV38	-	Chr:10	22943549	22944189	+	DAAA02028089.1	Functional		
AV38-c*	Gene 12		AV38	-	Chr:10	22951620	22952260	+	DAAA02028089.1	Functional		
AV38-d			AV38	-	Chr:10	22954498	22955119	+	DAAA02028089.1	Functional		
AV37-a			AV37	-	Chr:10	22959482	22960033	+	DAAA02028089.1	Psuedogene		
AV38-e			AV38	-	Chr:10	22988184	22988805	+	DAAA02028090.1	Functional		
AV37-b			AV37	-	Chr:10	22993493	22994042	+	DAAA02028090.1	Psuedogene		
AV36-a*	Gene 384		AV36	-	Chr:10	23027669	23028262	+	DAAA02028090.1	Functional		
AV35-a			AV35	-	Chr:10	23035476	23036105	+	DAAA02028090.1	Functional		
AV38-f			AV38	-	Chr:10	23050097	23050686	+	DAAA02028100.1	Incomplete		
AV33-a			AV33	-	Chr:10	23055926	23056526	+	DAAA02028101.1	Functional		
AV29-a			AV29	-	Chr:10	23059464	23060028	+	DAAA02028101.1	Psuedogene		
AV28-b			AV28	-	Chr:10	23065458	23066050	+	DAAA02028101.1	Functional		
AV33-b			AV33	-	Chr:10	23069171	23069520	+	DAAA02028101.1	Psuedogene		
AV34-a			AV34	-	Chr:10	23076623	23077258	+	DAAA02028101.1	Psuedogene		
AV26-j			AV26	-	Chr:10	23079664	23079809	+	DAAA02028101.1	Psuedogene		
AV33-c			AV33	-	Chr:10	23091059	23091650	+	DAAA02028101.1	Psuedogene		
AV29-b			AV29	-	Chr:10	23099357	23099928	+	DAAA02028101.1	Functional		
AV34-b			AV34	-	Chr:10	23109878	23110543	+	DAAA02028102.1	Psuedogene		
AV8-f			AV8	-	Chr:10	23130649	23131158	+	DAAA02028106.1	Functional		
AV26-k*	Gene 363		AV26	-	Chr:10	23139422	23140233	+	DAAA02028107.1	Functional		
AV25-g			AV25	-	Chr:10	23150417	23151048	+	DAAA02028107.1	Psuedogene		
AV24-d*	Gene 262		AV24	-	Chr:10	23157065	23157612	+	DAAA02028107.1	Functional		
AV26-l			AV26	-	Chr:10	23164563	23165700	+	DAAA02028108.1	Functional		
AV26-m			AV26	-	Chr:10	23172155	23173295	+	DAAA02028108.1	Functional		
DV1-n*		DV1s22-1	DV1	-	Chr:10	23181743	23182317	+	DAAA02028109.1	Functional		

DV1-o			DV1	-	Chr:10	23196256	23196863	+	DAAA02028110.1	Functional		
AV23-f*	Gene 248		AV23	-	Chr:10	23202966	23203491	+	DAAA02028110.1	Functional		
AV22-i			AV22	-	Chr:10	23207866	23208280	+	DAAA02028112.1	Incomplete		
DV1-p*	Gene 247		DV1	-	Chr:10	23212886	23213516	+	DAAA02028112.1	Functional		
AV23-g*	Gene 249		AV23	-	Chr:10	23221405	23221949	+	DAAA02028113.1	Functional		
DV1-q			DV1	-	Chr:10	23231387	23231665	+	DAAA02028113.1	Incomplete		
DV1-r*	Gene 89	DV1s9-1	DV1	-	Chr:10	23240851	23241465	+	DAAA02028114.1	Psuedogene		
AV23-h			AV23	-	Chr:10	23250003	23250548	+	DAAA02028114.1	Psuedogene		
AV22-j			AV22	-	Chr:10	23251198	23251733	+	DAAA02028114.1	Psuedogene		
AV26-n			AV26	-	Chr:10	23257689	23258501	+	DAAA02028114.1	Functional		
DV1-s*	Gene 42	DV1s10-1	DV1	-	Chr:10	23267117	23267713	+	DAAA02028114.1	Functional		
AV25-h*	Gene 182		AV25	-	Chr:10	23273756	23274388	+	DAAA02028114.1	Functional		
AV23-i*	Gene 183		AV23	-	Chr:10	23276986	23277526	+	DAAA02028114.1	Psuedogene		
AV22-k			AV22	-	Chr:10	23278175	23278499	+	DAAA02028114.1	Psuedogene		
AV26-o*	Gene 185		AV26	-	Chr:10	23281633	23282438	+	DAAA02028114.1	Psuedogene		
DV1-t*	Gene 186	DV1s24-1	DV1	-	Chr:10	23291265	23291858	+	DAAA02028114.1	Functional		
AV25-i			AV25	-	Chr:10	23297598	23298216	+	DAAA02028114.1	Functional		
AV21-a*	Gene 188		AV21	-	Chr:10	23303762	23304334	+	DAAA02028114.1	Functional		
AV20-a*	Gene 189		AV20	-	Chr:10	23312082	23312623	+	DAAA02028114.1	Functional		
AV19-b*	Gene 190		AV19	-	Chr:10	23320406	23321109	+	DAAA02028114.1	Psuedogene		
AVX-c*	Gene 191		AVX	-	Chr:10	23323954	23324458	+	DAAA02028114.1	Functional		
AVX-d*	Gene 192		AVX	-	Chr:10	23330884	23331395	+	DAAA02028114.1	Functional		
AVX-e*	Gene 193		AVX	-	Chr:10	23342744	23343256	+	DAAA02028114.1	Functional		
AV18-b*	Gene 194		AV18	-	Chr:10	23344761	23345281	+	DAAA02028114.1	Functional		
AV12-a*	Gene 195		AV12	-	Chr:10	23365683	23366271	+	DAAA02028114.1	Functional		
AV11-a			AV11	-	Chr:10	23378100	23379317	+	DAAA02028114.1	Psuedogene		
AV10-a			AV10	-	Chr:10	23380262	23380869	+	DAAA02028114.1	Psuedogene		
AV9-a*	Gene 198		AV9	-	Chr:10	23385299	23385795	+	DAAA02028114.1	Psuedogene		
AV14-a			AV14	-	Chr:10	23393563	23394110	+	DAAA02028114.1	Functional		

AV13-a*	Gene 199		AV13	-	Chr:10	23397807	23398356	+	DAAA02028114.1	Psuedogene		
AVY-b*	Gene 200		AVY	-	Chr:10	23403929	23404497	+	DAAA02028114.1	Functional		
AV9-b*	Gene 201		AV9	-	Chr:10	23407913	23408414	+	DAAA02028114.1	Psuedogene		
AV14-b			AV14	-	Chr:10	23427454	23428573	+	DAAA02028114.1	Psuedogene		
AV23-j*	Gene 373		AV23	-	Chr:10	23447009	23447537	+	DAAA02028116.1	Psuedogene		
AV22-l			AV22	-	Chr:10	23448188	23448748	+	DAAA02028116.1	Psuedogene		
DV1-u			DV1	-	Chr:10	23459145	23459737	+	DAAA02028116.1	Functional		
AV25-j*	Gene 376		AV25	-	Chr:10	23465670	23466300	+	DAAA02028116.1	Functional		
AV23-k*	Gene 378		AV23	-	Chr:10	23468888	23469434	+	DAAA02028116.1	Psuedogene		
AV22-m*	Gene 379		AV22	-	Chr:10	23470079	23470639	+	DAAA02028116.1	Psuedogene		
AV26-p*	Gene 380		AV26	-	Chr:10	23476595	23477404	+	DAAA02028116.1	Functional		
DV1-v			DV1	-	Chr:10	23486840	23487437	+	DAAA02028116.1	Psuedogene		
AV25-k			AV25	-	Chr:10	23493599	23494233	+	DAAA02028116.1	Functional		
AV23-l			AV23	-	Chr:10	23496909	23497450	+	DAAA02028116.1	Psuedogene		
AV22-n			AV22	-	Chr:10	23498102	23498662	+	DAAA02028116.1	Functional		
AV26-q*	Gene 377/336		AV26	-	Chr:10	23501110	23501920	+	DAAA02028116.1	Psuedogene		
DV1-w*		DV1s34-2	DV1	-	Chr:10	23511326	23511929	+	DAAA02028116.1	Functional		
AV25-l*	Gene 326		AV25	-	Chr:10	23517595	23518218	+	DAAA02028116.1	Functional		
AV26-r*	Gene 331		AV26	-	Chr:10	23524272	23525404	+	DAAA02028116.1	Functional		
DV1-x*	BoTRDV1.31	DV1s25-2	DV1	-	Chr:10	23535596	23536211	+	DAAA02028116.1	Functional		
AV23-m*	Gene 202/329		AV23	-	Chr:10	23540770	23541303	+	DAAA02028116.1	Psuedogene		
AV22-o			AV22	-	Chr:10	23549339	23549910	+	DAAA02028116.1	Psuedogene		
AV24-e			AV24	-	Chr:10	23554690	23555231	+	DAAA02028116.1	Psuedogene		
DV1-y			DV1	-	Chr:10	23567044	23567649	+	DAAA02028116.1	Functional		
AV23-n			AV23	-	Chr:10	23571788	23572347	+	DAAA02028116.1	Psuedogene		
AV22-p*	Gene 206/232		AV22	-	Chr:10	23577090	23577668	+	DAAA02028116.1	Psuedogene		
AV24-f			AV24	-	Chr:10	23581698	23582251	+	DAAA02028116.1	Psuedogene		
DV1-z*	Gene 321	DV1s31-1	DV1	-	Chr:10	23586938	23587548	+	DAAA02028116.1	Functional		

AV22-q*	Gene 320		AV22	-	Chr:10	23592483	23593043	+	DAAA02028116.1	Functional		
AV26-s			AV26	-	Chr:10	23595541	23596350	+	DAAA02028116.1	Functional		
AV25-m*	Gene 156/318		AV25	-	Chr:10	23605890	23606523	+	DAAA02028116.1	Functional		
DV1-aa			DV1	-	Chr:10	23618035	23618641	+	DAAA02028116.1	Functional		
AV23-o*	Gene 316		AV23	-	Chr:10	23624547	23625092	+	DAAA02028116.1	Functional		
AV22-r*	Gene 315		AV22	-	Chr:10	23631953	23632510	+	DAAA02028116.1	Psuedogene		
AV8-g*	Gene 314/160		AV8	-	Chr:10	23636381	23636869	+	DAAA02028116.1	Functional		
AV21-b*	Gene 313		AV21	-	Chr:10	23639854	23640425	+	DAAA02028116.1	Functional		
AV20-b*	Gene 311		AV20	-	Chr:10	23647889	23648424	+	DAAA02028116.1	Functional		
AV6-a			AV6	-	Chr:10	23683754	23684284	+	DAAA02028124.1	Incomplete		
DV1-ab*	BoTRDV1.32	DV1s27-2	DV1	-	Chr:10	23720626	23721233	+	DAAA02028129.1	Functional		
AV22-s*	Gene 208		AV22	-	Chr:10	23727226	23727786	+	DAAA02028129.1	Functional		
AV26-t			AV26	-	Chr:10	23730243	23731051	+	DAAA02028129.1	Functional		
AV25-n			AV25	-	Chr:10	23737275	23737909	+	DAAA02028129.1	Psuedogene		
DV1-ac			DV1	-	Chr:10	23744316	23744921	+	DAAA02028129.1	Functional		
AV19-c*	Gene 310		AV19	-	Chr:10	23748128	23748758	+	DAAA02028130.1	Functional		
AVX-f*	Gene 309		AVX	-	Chr:10	23750612	23751371	+	DAAA02028130.1	Functional		
AVX-g*	Gene 308		AVX	-	Chr:10	23767027	23767538	+	DAAA02028130.1	Functional		
AVX-h*	Gene 307		AVX	-	Chr:10	23773137	23773646	+	DAAA02028131.1	Functional		
AVX-i*	Gene 306		AVX	-	Chr:10	23778826	23779335	+	DAAA02028131.1	Functional		
AV18-c			AV18	-	Chr:10	23783361	23783874	+	DAAA02028131.1	Psuedogene		
AV17-a*	Gene 72		AV17	-	Chr:10	23794652	23795442	+	DAAA02028131.1	Functional		
AV16-a*	Gene 73		AV16	-	Chr:10	23801677	23802159	+	DAAA02028132.1	Functional		
AV14-c			AV14	-	Chr:10	23823839	23824965	+	DAAA02028134.1	Psuedogene		
AV13-b*	Gene 296		AV13	-	Chr:10	23829114	23829653	+	DAAA02028134.1	Functional		
AV12-b			AV12	-	Chr:10	23842486	23843073	+	DAAA02028136.1	Functional		
AV11-b			AV11	-	Chr:10	23852978	23853549	+	DAAA02028136.1	Psuedogene		
AV10-b*	Gene 78		AV10	-	Chr:10	23854487	23855099	+	DAAA02028136.1	Functional		

AV9-c*	Gene 302		AV9	-	Chr:10	23862145	23862643	+	DAAA02028136.1	Psuedogene		
AV14-d			AV14	-	Chr:10	23871677	23872220	+	DAAA02028136.1	Psuedogene		
AV13-c*	Gene 300		AV13	-	Chr:10	23875405	23875955	+	DAAA02028136.1	Pseudogene		
AVY-c*	Gene 299		AVY	-	Chr:10	23879813	23880370	+	DAAA02028136.1	Functional		
AV20-c*	Gene 48		AV20	+	Chr:10	23888939	23889462	+	DAAA02028138.1	Functional		
AV26-u			AV26	+	Chr:10	23900317	23901458	+	DAAA02028139.1	Functional		
AV23-p			AV23	+	Chr:10	23905670	23905948	+	DAAA02028139.1	Psuedogene		
DV1-ad*	Gene 46		DV1	+	Chr:10	23910774	23911289	+	DAAA02028139.1	Pseudogene		
DV1-ae*	Gene 45	DV1s37-1	DV1	+	Chr:10	23917231	23917831	+	DAAA02028139.1	Functional		
AV24-g*	Gene 44		AV24	+	Chr:10	23923492	23924039	+	DAAA02028139.1	Psuedogene		
AV25-o			AV25	+	Chr:10	23927453	23928089	+	DAAA02028139.1	Psuedogene		
DV1-af*	Gene 42	DV1s38-1	DV1	+	Chr:10	23933924	23934520	+	DAAA02028139.1	Functional		
AV26-v			AV26	+	Chr:10	23952004	23953132	+	DAAA02028142.1	Psuedogene		
DV1-ag*		DV1s4-2	DV1	+	Chr:10	23959743	23960346	+	DAAA02028143.1	Functional		
AV24-h			AV24	+	Chr:10	23965646	23966191	+	DAAA02028143.1	Psuedogene		
AV25-p			AV25	+	Chr:10	23971979	23972611	+	DAAA02028143.1	Functional		
DV1-ah*	Gene 265	DV1s5-1	DV1	+	Chr:10	23978150	23978745	+	DAAA02028143.1	Functional		
AV26-w*	Gene 266		AV26	+	Chr:10	23984689	23985499	+	DAAA02028143.1	Functional		
DV1-ai			DV1	+	Chr:10	23995847	23996448	+	DAAA02028144.1	Functional		
AV8-h*	Gene 268/287		AV8	+	Chr:10	24007152	24007631	+	DAAA02028144.1	Psuedogene		
AV22-t*	Gene 286/269		AV22	+	Chr:10	24008004	24008570	+	DAAA02028144.1	Functional		
AV26-x			AV26	-	Chr:10	24026000	24027044	+	DAAA02028148.1	Incomplete		
DV1-aj*	Gene 244	DV1s49-1	DV1	-	Chr:10	24028336	24028836	+	DAAA02028149.1	Functional		
AV26-y			AV26	-	Chr:10	24040176	24041310	+	DAAA02028149.1	Psuedogene		
AVY-d			AVY	+	Chr:10	24049503	24050060	+	DAAA02028151.1	Functional		
AV9-d			AV9	-	Chr:10	24054652	24054988	+	DAAA02028153.1	Incomplete		
AV8-i*	Gene 64		AV8	-	Chr:10	24062432	24062915	+	DAAA02028153.1	Functional		
AV26-z*	Gene 65		AV26	-	Chr:10	24080740	24081553	+	DAAA02028154.1	Psuedogene		

AV25-q			AV25	-	Chr:10	24091660	24092289	+	DAAA02028154.1	Psuedogene		
DV1-ak			DV1	-	Chr:10	24099007	24099612	+	DAAA02028154.1	Functional		
AV23-q*	Gene 67		AV23	-	Chr:10	24105088	24105633	+	DAAA02028154.1	Psuedogene		
AV19-d*	Gene 49		AV19	-	Chr:10	24109596	24110232	+	DAAA02028155.1	Functional	AMB	
AVX-j			AVX	-	Chr:10	24111782	24112287	+	DAAA02028155.1	Functional	AMB	
AV22-u			AV22	-	Chr:10	24115721	24116278	+	DAAA02028156.1	Functional		
AV8-j*	Gene 69		AV8	-	Chr:10	24120142	24120630	+	DAAA02028156.1	Functional		
AV21-c*	Gene 70/312		AV21	-	Chr:10	24128548	24129119	+	DAAA02028156.1	Functional		
AV20-d			AV20	-	Chr:10	24132045	24132580	+	DAAA02028156.1	Functional		
AV19-e			AV19	-	Chr:10	24140329	24140985	+	DAAA02028156.1	Psuedogene		
AVX-k			AVX	-	Chr:10	24142546	24143051	+	DAAA02028156.1	Functional		
AVX-l			AVX	-	Chr:10	24149270	24149779	+	DAAA02028156.1	Functional		
AVX-m			AVX	-	Chr:10	24156559	24157069	+	DAAA02028156.1	Functional		
AVX-n*	Gene 51		AVX	-	Chr:10	24175745	24176256	+	DAAA02028158.1	Functional		
AVX-o*	Gene 52		AVX	-	Chr:10	24197766	24198277	+	DAAA02028158.1	Functional		
AV18-d*	Gene 53		AV18	-	Chr:10	24201940	24202460	+	DAAA02028158.1	Functional		
AV12-c*	Gene 55		AV12	-	Chr:10	24218758	24219345	+	DAAA02028158.1	Functional		
AV11-c*	Gene 56		AV11	-	Chr:10	24231371	24231941	+	DAAA02028158.1	Psuedogene		
AV10-c*	Gene 57		AV10	-	Chr:10	24232705	24233186	+	DAAA02028158.1	Psuedogene		
AV13-d*	Gene 61/58		AV13	-	Chr:10	24238205	24238751	+	DAAA02028158.1	Functional		
AV9-e			AV9	-	Chr:10	24242881	24243387	+	DAAA02028158.1	Psuedogene		
AVX-p*	Gene 74		AVX	-	Chr:10	24262149	24262659	+	DAAA02028161.1	Functional		
AV18-e*	Gene 75		AV18	-	Chr:10	24266301	24266821	+	DAAA02028161.1	Psuedogene		
AV12-d*	Gene 76		AV12	-	Chr:10	24283143	24283728	+	DAAA02028161.1	Functional		
AV11-e			AV11	-	Chr:10	24292968	24293540	+	DAAA02028262.1	Psuedogene		
AV10-d			AV10	-	Chr:10	24294490	24295098	+	DAAA02028161.1	Functional		
AV9-f			AV9	-	Chr:10	24299528	24300026	+	DAAA02028161.1	Psuedogene		
AV14-e			AV14	-	Chr:10	24309115	24309661	+	DAAA02028161.1	Functional		
AV13-e			AV13	-	Chr:10	24312820	24313369	+	DAAA02028161.1	Functional		

AVY-e			AVY	-	Chr:10	24317208	24317765	+	DAAA02028161.1	Functional		
AV6-b			AV6	-	Chr:10	24336941	24337171	+	DAAA02028163.1	Psuedogene		
AV5-a			AV5	-	Chr:10	24346160	24346711	+	DAAA02028163.1	Functional		
AV4-a			AV4	-	Chr:10	24357279	24358153	+	DAAA02028163.1	Functional		
AV14-f			AV14	-	Chr:10	24448557	24449660	+	DAAA02028239.1	Psuedogene		
AV13-f			AV13	-	Chr:10	24453902	24454441	+	DAAA02028239.1	Functional		
AV9-g			AV9	-	Chr:10	24457553	24458060	+	DAAA02028239.1	Psuedogene		
DV1-al*	Gene 291	DV1s29-1	DV1	-	Chr:10	24465666	24466273	+	DAAA02028240.1	Functional		
AVY-f*	Gene 299		AVY	-	Chr:10	24485081	24485638	+	DAAA02028242.1	Functional		
AV9-h*	Gene 298		AV9	-	Chr:10	24490204	24490695	+	DAAA02028242.1	Psuedogene		
DV1-am			DV1	-	Chr:10	24494855	24495456	+	DAAA02028243.1	Functional		
AV8-k			AV8	+	Chr:10	24502277	24502774	+	DAAA02028244.1	Psuedogene		
DV1-an*	BoTRDV1.15	DV1s40-1	DV1	-	Chr:10	24511917	24512517	+	DAAA02028247.1	Functional		
AV8-l*	Gene 364		AV8	-	Chr:10	24519435	24519944	+	DAAA02028244.1	Functional		
AV26-aa			AV26	-	Chr:10	24532911	24533716	+	DAAA02028251.1	Functional		
DV1-ao			DV1	-	Chr:10	24541572	24542167	+	DAAA02028251.1	Functional		
AV25-r			AV25	-	Chr:10	24547576	24548208	+	DAAA02028251.1	Functional		
AV24-i			AV24	-	Chr:10	24554392	24554937	+	DAAA02028251.1	Psuedogene		
DV1-ap			DV1	-	Chr:10	24560197	24560798	+	DAAA02028251.1	Psuedogene		
AV26-ab			AV26	-	Chr:10	24565384	24566520	+	DAAA02028251.1	Psuedogene		
DV1-aq			DV1	-	Chr:10	24574066	24574566	+	DAAA02028251.1	Functional		
AV26-ac			AV26	-	Chr:10	24585426	24586565	+	DAAA02028251.1	Functional		
DV1-ar*	Gene 258	DV1s2-2	DV1	-	Chr:10	24600541	24601116	+	DAAA02028253.1	Functional		
DV1-as*	Gene 138	DV1s1-1	DV1	-	Chr:10	24617556	24618165	+	DAAA02028253.1	Functional		
AV23-r			AV23	-	Chr:10	24623322	24623700	+	DAAA02028253.1	Psuedogene		
AV22-v*	Gene 139		AV22	-	Chr:10	24628717	24629272	+	DAAA02028253.1	Psuedogene		
DV1-at*	Gene 140		DV1	-	Chr:10	24634116	24634726	+	DAAA02028253.1	Psuedogene		
AV23-s*	Gene 141		AV23	-	Chr:10	24643687	24644235	+	DAAA02028253.1	Psuedogene		
AV22-w*	Gene 335		AV22	-	Chr:10	24646142	24646699	+	DAAA02028253.1	Functional		

AV26-ad*	Gene 334		AV26	-	Chr:10	24649143	24649954	+	DAAA02028253.1	Functional		
DV1-au			DV1	-	Chr:10	24657755	24658363	+	DAAA02028253.1	Functional		
AV25-s*	Gene 123		AV25	-	Chr:10	24664103	24664725	+	DAAA02028253.1	Psuedogene		
DV1-av*	Gene 137		DV1	-	Chr:10	24674694	24675301	+	DAAA02028254.1	Psuedogene		
AV22-x*	Gene 136/121		AV22	-	Chr:10	24680216	24680776	+	DAAA02028254.1	Functional		
AV26-ae*	Gene 120		AV26	-	Chr:10	24683272	24684086	+	DAAA02028254.1	Functional		
AV25-t*	Gene 237		AV25	-	Chr:10	24696506	24697488	+	DAAA02028254.1	Functional		
DV1-aw*	Gene 118		DV1	-	Chr:10	24703626	24704231	+	DAAA02028254.1	Functional		
AV23-t*	Gene 117/235		AV23	-	Chr:10	24709718	24710263	+	DAAA02028254.1	Psuedogene		
AVX-q*	Gene 116		AVX	-	Chr:10	24734925	24735436	+	DAAA02028256.1	Functional		
AVX-r			AVX	-	Chr:10	24759310	24759821	+	DAAA02028259.1	Functional		
AVX-s*	Gene 114		AVX	-	Chr:10	24764887	24765398	+	DAAA02028259.1	Functional		
AV18-f*	Gene 113		AV18	-	Chr:10	24770367	24770888	+	DAAA02028259.1	Psuedogene		
AV17-b			AV17	+	Chr:10	24781716	24782504	+	DAAA02028259.1	Psuedogene		
AV14-g			AV14	-	Chr:10	24801831	24802961	+	DAAA02028260.1	Functional		
AV12-e			AV12	-	Chr:10	24807295	24807750	+	DAAA02028261.1	Incomplete		
AV11-d			AV11	-	Chr:10	24817354	24817927	+	DAAA02028161.1	Psuedogene		
AV10-e*	Gene 303		AV10	-	Chr:10	24818880	24819489	+	DAAA02028262.1	Functional		
AV9-i			AV9	-	Chr:10	24823985	24824480	+	DAAA02028262.1	Psuedogene		
AV14-h			AV14	-	Chr:10	24832945	24833492	+	DAAA02028262.1	Functional		
AV13-g			AV13	-	Chr:10	24836681	24837226	+	DAAA02028262.1	Functional		
AVY-g*	Gene 82		AVY	-	Chr:10	24838384	24838941	+	DAAA02028262.1	Functional		
AV24-j*	Gene 132/98		AV24	-	Chr:10	24847585	24848131	+	DAAA02028265.1	Psuedogene		
DV1-ax*	Gene 97	DV1s14-1	DV1	-	Chr:10	24852524	24853126	+	DAAA02028265.1	Functional		
AV26-af			AV26	-	Chr:10	24857714	24858850	+	DAAA02028265.1	Psuedogene		
DV1-ay*		DV1s12-1	DV1	-	Chr:10	24865173	24865673	+	DAAA02028265.1	Functional		
AV26-ag*	Gene 95		AV26	-	Chr:10	24875657	24876793	+	DAAA02028265.1	Functional		
AV6-c			AV6	+	Chr:10	24885032	24885584	+	DAAA02028267.1	Psuedogene		

AV6-d			AV6	+	Chr:10	24885884	24886436	+	DAAA02028268.1	Psuedogene		
AV5-b*	Gene 87		AV5	-	Chr:10	24902611	24903163	+	DAAA02028277.1	Functional		
AV13-h			AV13	+	Chr:10	24909812	24910351	+	DAAA02028278.1	Functional		
DV1-az*		DV1s15-2	DV1	-	Chr:10	24920012	24920168	+	DAAA02028280.1	Incomplete		
DV1-ba*	Gene 94	DV1s11-1	DV1	-	Chr:10	24932109	24932747	+	DAAA02028282.1	Functional		
AV4-b			AV4	-	Chr:10	24948745	24949619	+	DAAA02028283.1	Functional		
AV23-u*	Gene 93		AV23	-	Chr:10	24959516	24960058	+	DAAA02028284.1	Psuedogene		
AV26-ah*	Gene 129		AV26	-	Chr:10	24967750	24968891	+	DAAA02028285.1	Functional		
DV1-bb*	Gene 276	DV1s21-2	DV1	-	Chr:10	24992843	24993451	+	DAAA02028285.1	Functional		
AV23-v*	Gene 249		AV23	-	Chr:10	24997916	24998457	+	DAAA02028285.1	Psuedogene		
AV22-y			AV22	-	Chr:10	25003151	25003592	+	DAAA02028285.1	Psuedogene		
AVX-t			AVX	-	Chr:10	25018494	25019002	+	DAAA02028286.1	Psuedogene		
AV16-b*	Gene 217/371		AV16	+	Chr:10	25034456	25034937	+	DAAA02028287.1	Functional		
AV17-c*	Gene 216/370/283		AV17	+	Chr:10	25040218	25041005	+	DAAA02028287.1	Psuedogene		
AV18-g*	Gene 215/369		AV18	+	Chr:10	25053543	25054057	+	DAAA02028287.1	Psuedogene		
AVX-u*	Gene 367		AVX	+	Chr:10	25058084	25058382	+	DAAA02028287.1	Incomplete		
AV18-h			AV18	+	Chr:10	25059685	25060202	+	DAAA02028288.1	Functional		
AVX-v			AVX	+	Chr:10	25063816	25064329	+	DAAA02028288.1	Psuedogene		
AVX-w			AVX	+	Chr:10	25069836	25070347	+	DAAA02028289.1	Functional		
AV26-ai			AV26	+	Chr:10	25080384	25081196	+	DAAA02028291.1	Functional		
AVX-x			AVX	+	Chr:10	25090080	25090592	+	DAAA02028292.1	Functional		
AVX-y*	Gene 348		AVX	+	Chr:10	25109417	25110166	+	DAAA02028294.1	Psuedogene		
AV19-f			AV19	+	Chr:10	25111710	25112343	+	DAAA02028294.1	Functional		
AV25-u			AV25	+	Chr:10	25120865	25121495	+	DAAA02028294.1	Psuedogene		
DV1-bc*	Gene 351	DV1s20-1	DV1	+	Chr:10	25127239	25127837	+	DAAA02028294.1	Functional		
AV26-aj*	Gene 352		AV26	+	Chr:10	25136250	25137062	+	DAAA02028294.1	Functional		
AV22-z*	Gene 353		AV22	+	Chr:10	25139502	25140062	+	DAAA02028294.1	Functional		
AV23-w			AV23	+	Chr:10	25140700	25141240	+	DAAA02028294.1	Psuedogene		

AVX-z*	Gene 366		AVX	-	Chr:10	25161604	25162115	+	DAAA02028296.1	Functional	AMB	
AVX-aa*	Gene 367		AVX	-	Chr:10	25168344	25168853	+	DAAA02028296.1	Functional	AMB	
DV1-bd			DV1	+	Chr:10	25173058	25173277	+	DAAA02028297.1	Incomplete		
AV12-f			AV12	-	Chr:10	25193590	25194177	+	DAAA02028300.1	Functional		
AV11-f			AV11	-	Chr:10	25206225	25206795	+	DAAA02028300.1	Psuedogene		
AV10-f			AV10	-	Chr:10	25207560	25208042	+	DAAA02028300.1	Psuedogene		
AV13-i			AV13	-	Chr:10	25213065	25213263	+	DAAA02028300.1	Incomplete		
AV13-j*	Gene 60/342		AV13	-	Chr:10	25216585	25217131	+	DAAA02028302.1	Functional		
AV9-j*	Gene 108		AV9	-	Chr:10	25221236	25221738	+	DAAA02028302.1	Psuedogene		
AV4-c			AV4	-	Chr:10	25230662	25231525	+	DAAA02028302.1	Psuedogene		
AV22-aa			AV22	-	Chr:10	25244820	25245386	+	DAAA02028303.1	Functional		
AV8-m			AV8	-	Chr:10	25245760	25246234	+	DAAA02028303.1	Psuedogene		
DV1-be*	Gene 150	DV1s6-2	DV1	-	Chr:10	25255950	25256544	+	DAAA02028304.1	Functional		
AV26-ak			AV26	-	Chr:10	25265502	25266354	+	DAAA02028305.1	Incomplete		
DV1-bf*	Gene 280/153	DV1s7-2	DV1	-	Chr:10	25296972	25297580	+	DAAA02028306.1	Functional		
AV22-ab*	Gene 154		AV22	-	Chr:10	25303580	25304137	+	DAAA02028306.1	Functional		
AV26-al			AV26	-	Chr:10	25306592	25307400	+	DAAA02028306.1	Functional		
AV25-v			AV25	-	Chr:10	25320268	25320901	+	DAAA02028306.1	Functional		
DV1-bg*	Gene 157	DV1s8-1	DV1	-	Chr:10	25341876	25342481	+	DAAA02028307.1	Functional		
AV23-x*	Gene 158		AV23	-	Chr:10	25348338	25348883	+	DAAA02028307.1	Functional		
AV22-ac*	Gene 68/159		AV22	-	Chr:10	25357331	25357888	+	DAAA02028307.1	Functional		
AV8-n			AV8	-	Chr:10	25361725	25362213	+	DAAA02028307.1	Functional		
AV21-d*	Gene 161		AV21	-	Chr:10	25370239	25370812	+	DAAA02028307.1	Psuedogene		
AV20-e*	Gene 162		AV20	-	Chr:10	25378267	25378800	+	DAAA02028307.1	Functional		
AV19-g*	Gene 163		AV19	-	Chr:10	25386522	25387169	+	DAAA02028307.1	Functional		
AVX-ab*	Gene 164		AVX	-	Chr:10	25388718	25389222	+	DAAA02028307.1	Functional		
AVX-ac*	Gene 165		AVX	-	Chr:10	25397210	25397717	+	DAAA02028307.1	Psuedogene		
AV3-a*	Gene 166		AV3	-	Chr:10	25416553	25417024	+	DAAA02028307.1	Functional		

AV2-a*	Gene167		AV2	-	Chr:10	25421891	25422379	+	DAAA02028308.1	Functional		
AV3-b*	Gene168		AV3	-	Chr:10	25440313	25440784	+	DAAA02028307.1	Functional		
AV2-b*	Gene169		AV2	-	Chr:10	25449384	25449872	+	DAAA02028308.1	Functional		
AV3-c*	Gene170		AV3	-	Chr:10	25467843	25468314	+	DAAA02028307.1	Functional		
AV2-c*	Gene171		AV2	-	Chr:10	25474836	25475326	+	DAAA02028307.1	Functional		
AV3-d*	Gene172		AV3	-	Chr:10	25489825	25490291	+	DAAA02028307.1	Functional		
AV2-d*	Gene173		AV2	-	Chr:10	25496856	25497344	+	DAAA02028307.1	Psuedogene		
AV3-e			AV3	-	Chr:10	25508599	25509070	+	DAAA02028308.1	Functional		
AV2-e*	Gene175		AV2	-	Chr:10	25515891	25516385	+	DAAA02028307.1	Psuedogene		
AV3-f*	Gene176		AV3	-	Chr:10	25530576	25531042	+	DAAA02028308.1	Functional		
AV2-f*	Gene177		AV2	-	Chr:10	25537400	25537883	+	DAAA02028308.1	Functional		
AV3-g*	Gene178		AV3	-	Chr:10	25547747	25548213	+	DAAA02028308.1	Functional		
AV2-g*	Gene179		AV2	-	Chr:10	25554768	25555258	+	DAAA02028308.1	Functional		
AV1-a*	Gene 180		AV1	-	Chr:10	25628625	25629292	+	DAAA02028308.1	Functional		
AV9-k*	Gene 295		AV9	+	Chr:10	60202862	60203365	+	DAAA02029076.1	Functional		
AV13-k*	Gene 296		AV13	+	Chr:10	60207211	60207750	+	DAAA02029076.1	Functional		
AV14-i			AV14	+	Chr:10	60211894	60212611	+	DAAA02029076.1	Incomplete		
DV1-bh			DV1	-	Chr:9	71355801	71356402	+	DAAA02026655.1	Orphon		
AV33-d*	BoTRDV2.2		AV33	-	DAAA02066600.1	1506	2062	+	DAAA02066600.1	Functional		
AV29-c*	Gene 220		AV29	-	DAAA02066600.1	4996	5567	+	DAAA02066600.1	Functional		
AV28-c			AV28	-	DAAA02066600.1	11292	11883	+	DAAA02066600.1	Functional		
AV28-d			AV28	-	DAAA02066600.1	12370	12491	+	DAAA02066600.1	Psuedogene		
AV33-e			AV33	-	DAAA02066600.1	14997	15344	+	DAAA02066600.1	Psuedogene		
AV34-c			AV34	-	DAAA02066600.1	25454	26115	+	DAAA02066600.1	Psuedogene		
AV26-am			AV26	-	DAAA02066600.1	29241	30055	+	DAAA02066600.1	Psuedogene		
AV33-f			AV33	-	DAAA02066600.1	48475	49067	+	DAAA02066600.1	Psuedogene		
