# Supplementary figures and images for: Genomic analysis offers insights into the evolution of the bovine TRA/TRD locus
Source: BMC Genomics. 2014 Nov 19;15(1):994. doi: 10.1186/1471-2164-15-994 (PMC4289303; doi:10.1186/1471-2164-15-994)

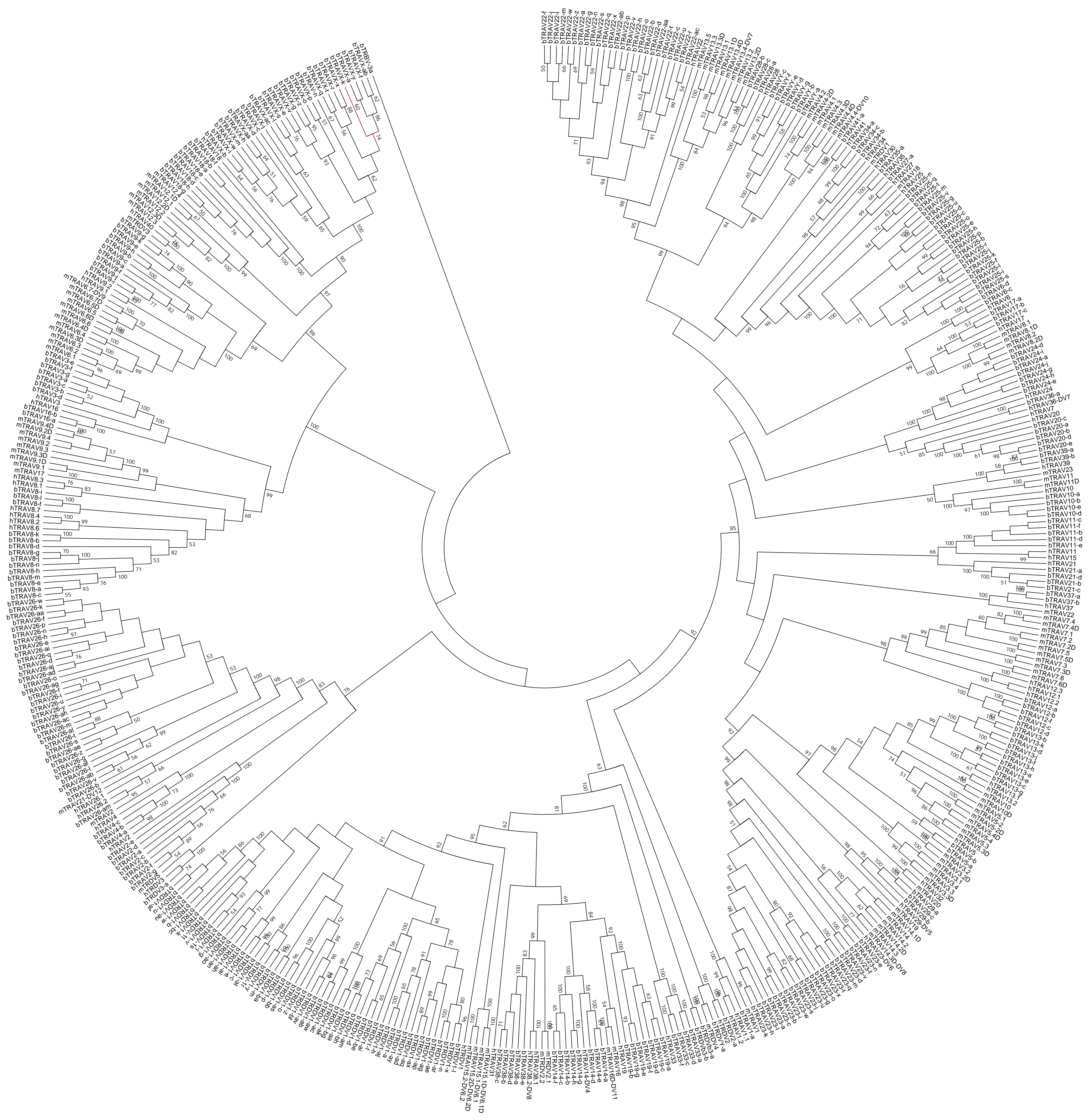

Supplement: Supplementary file 2 — Additional file 2: Neighbour-joining phylogenetic tree of all murine, human and bovine (from the UMD3.1 assembly) TRAV/TRDV genes. Analysis of the nucleotide sequence of the V-REGION (IMGT nomenclature) following pairwise deletion to remove gaps in the alignment. The final dataset included 400 positions. The sequence of bTRBV3a was used to root the tree. Based on a 1000 boot strap replicates the phylogenetically inferred orthologous TRAV/TRDV subgroups were supported by percentage bootstrap values (PB) of >90% in all cases except for bTRAV38 (PB = 83%), bTRAV5 (PB = 59%), bTRAV13 (PB = 85%) and bTRAV8 (PB = 68%). Sequence identity between bovine and human genes in orthologous groups ranged from 63.1-84.9%, sufficient to assign them as inter-species orthologues [47]. Generally the phylogenetically defined bovine TRAV/TRDV subgroups adhered to the convention of members sharing >75% nucleotide identity [[25], [26]]. However, within both the bTRDV1 and bTRAV8 subgroups identity between some members was <75% (down to 68.0 and 69.7% respectively) and conversely the identity between some bTRAV5/13 and some bTRAVX/18 members was >75%. Due to difficulties in alignment the following genes were excluded from the analysis i) bovine genes for which only incomplete or partial genomic sequences were available, ii) bTRAV11a – due to the presence of a large insert and iii) mTRAV15-3, mTRAV15D-3, hTRAV8.5. h = human, b = bovine and m = murine. (PDF 912 KB) [file 12864_2014_6826_MOESM2_ESM.pdf]

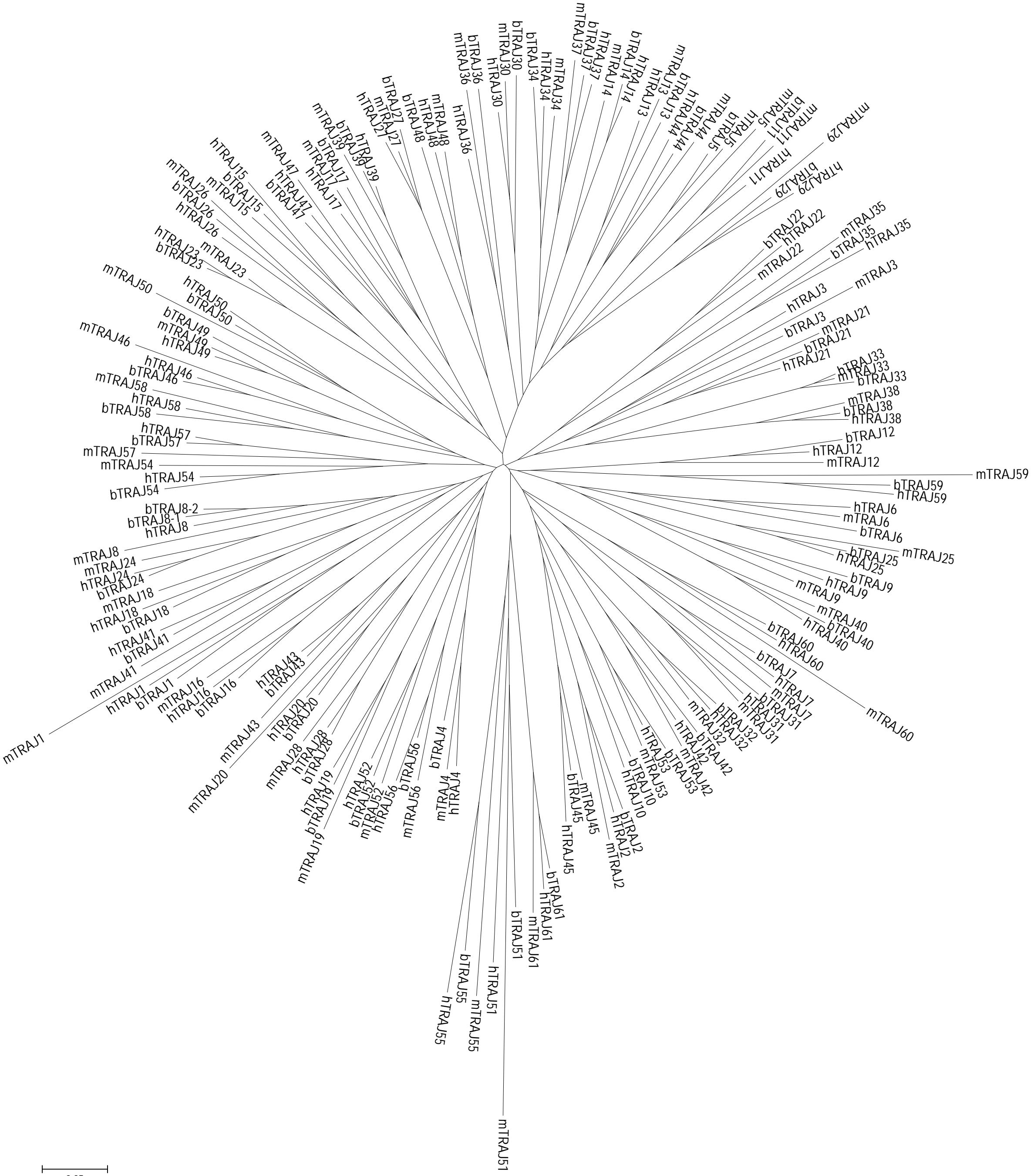

0.05

Supplement: Supplementary file 6 — Additional file 6: Neighbour-joining phylogenetic tree of all murine, human and bovine (from the UMD3.1 assembly) TRAJ genes. Analysis of the nucleotide sequence of the coding domain of TRAJ genes following pairwise deletion to remove gaps in the alignment. The final dataset had a total of 85 positions. Based on a 1000 boot strap replicates the orthologous TRAJ genes from mouse, human and cattle (where all genes were functional) formed phylogenetic groups supported by percentage bootstrap values (PB) of >75% (with the exception of TRAJ6 (PB =54%), 9 (53%) and 48 (67%)). PB values supporting the phylogenetic groups of TRAJ orthologues which included non-functional members were generally high but in several cases were <50%. h = human, b = bovine and m = murine. The formation of ‘triads’ composed of single genes from each species (except TRAJ10 which lacks a murine gene and TRAJ8 which contains 2 bovine genes) that have the same relative position in the genome (as denoted by their numerical designation) demonstrates conserved synteny. The level of nucleotide identity between orthologous bovine and human TRAJ genes ranges from 63.2% to 95.2%. (PDF 14 KB) [file 12864_2014_6826_MOESM6_ESM.pdf]

**A**

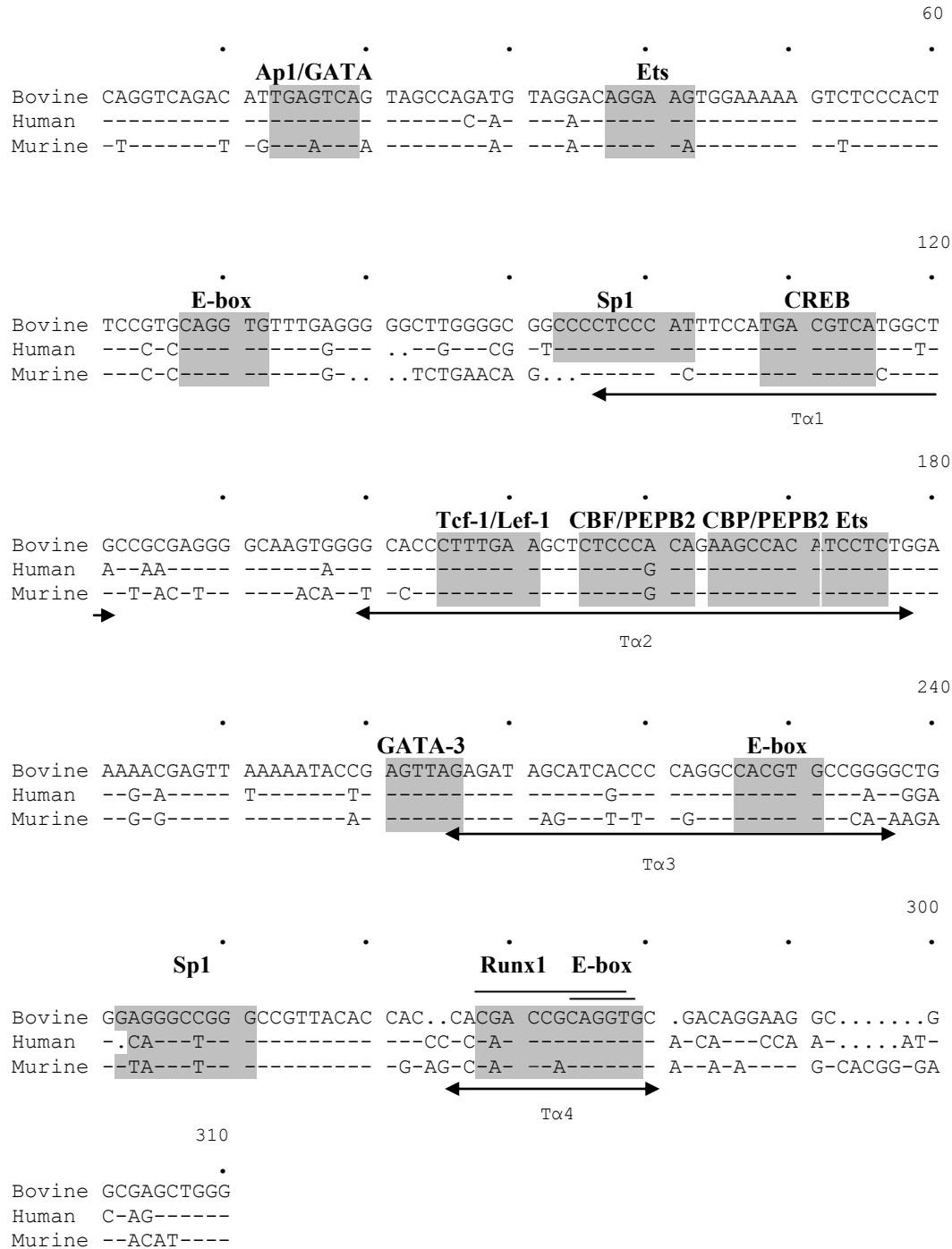

# B

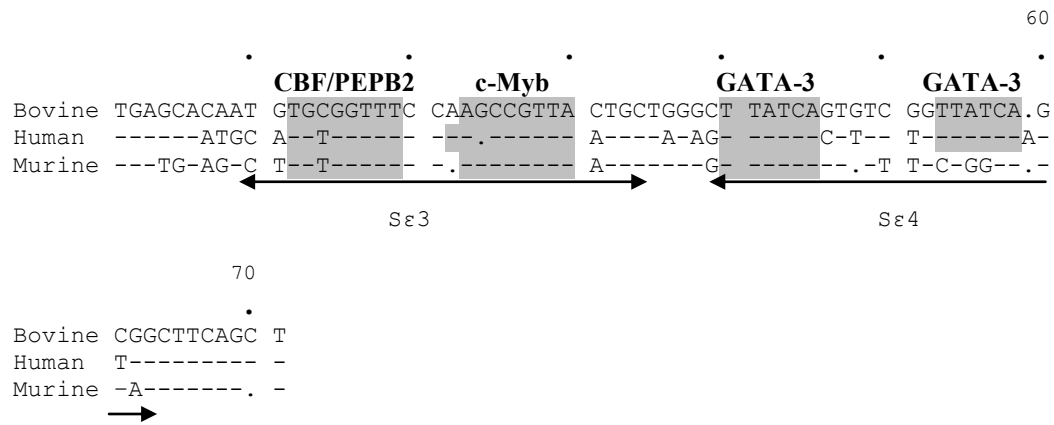

Supplement: Supplementary file 7 — Additional file 7: Sequence alignment of regulatory elements in the 3′ end of the bovine (from the UMD3.1 assembly), murine and human TRA/TRD locus. Sequences of defined transcription factor binding sites are shown in grey highlight. Nucleotide identity between orthologous sequences is shown by dashes and gaps by dots. (A) Alignment of the human, murine and putative bovine Eα sequences. Protein binding regions in the core human Eα (Tα1-4) are indicated by arrows. The 300 bp sequence of the putative bovine Eα shares 87.5% and 78.2% nucleotide identity with the human and murine Eα. Within the core Tα1-Tα2 fragment of the Eα, which constitutes the DNA scaffold for the generation of the nucleo-protein structure termed the ‘enhanceosome’ that is critical for Eα function [[48–50]], the CREB, TCF-1/LEF-1 and Ets binding sites show absolute conservation between the bovine, human and murine sequences. Simultaneous occupancy of these sites is a minimal requirement for Eα activity. Numerous other transcription factor binding sites which have been shown to be occupied in the Eα and play a role in appropriate regulation of Eα function [[51–53]] are also conserved in the putative bovine Eα sequence. (B) Alignment of the human, murine and putative bovine Eδ sequences. Protein binding regions in the core human Eδ (Sϵ3 and Sϵ4) are indicated by arrows. The 70 bp sequence spanning the essential Sϵ3 and Sϵ4 core of the enhancer [54] shows 77.1% and 72.8% nucleotide identity to the corresponding human and murine sequences. The CBF/PEPB2 and c-myb binding sites within Sϵ3 which are critical for the formation and function of the Eδ ‘enhanceosome’ [55] are conserved in the bovine sequence as are 2 GATA-3 binding sequence in Sϵ4. (PDF 165 KB) [file 12864_2014_6826_MOESM7_ESM.pdf]
