# Supplementary material for: Genomic analysis offers insights into the evolution of the bovine TRA/TRD locus
Source: BMC Genomics. 2014 Nov 19;15(1):994. doi: 10.1186/1471-2164-15-994 (PMC4289303; doi:10.1186/1471-2164-15-994)
Supplement: Supplementary file 3 — Additional file 3: Comparison of the annotated TRA/TRD genes with previously published annotations of the bovine TRA/TRD locus and data available in the IMGT website (July 2014). Summary of comparisons with the TRA/TRD gene repertoires identified by Reinink and Van Rhijn [15], Herzig et al. [14] and present in the IMGT database (July 2014). Also summarised are the number of TRA/TRD genes which have been identified in previous annotation studies. (PDF 411 KB) [file 12864_2014_6826_MOESM3_ESM.pdf]

Comparison of the UMD3.1 annotation with previous annotations of the bovine TRA/TRD locus and data available in the IMGT website (July 2014).

TRAV/TRDV genes

The repertoires of the TRAV/TRDV genes identified by Reinink and Van Rhijn (2009), Herzig et al. (2010) and in our annotation of the UMD3.1 assembly are summarised below. The Reinink and Van Rhijn (2009) annotation was for both TRAV and TRDV genes and was based on the Btau4.0 assembly, whilst the Herzig et al. (2010) annotation was for TRDV genes and based on the Btau3.0 assembly.

Subgroup	Reinink and Van Rhijn (2009)					Herzig et al. (2010)††	UMD3.1 assembly annotation					Percentage of genes identified in previous annotations‡
	Total	Functional	Pseudo.	Incomplete	Percentage functional	Total	Total	Functional	Pseudo.	Incomplete	Percentage functional	
	(Number of genes)					(Number of genes)	(Number of genes)					
AV1	1	1			100.0		1	1			100	100.0
AV2	7	6	1		85.7		7	5	2		71.4	100.0
AV3	7	6	1		85.7		7	7			100	85.7
AV4	5	3	2		60.0		3	2	1		66.7	0.0
AV5	6	6			100.0		2	2			100	50.0
AV6							4		3	1	0	0.0
AV7												
AV8	14	7	7		50.0		14	6	8		42.9	50.0
AV9	18	13	5		72.2		11	1	9	1	9.1	54.5
AV10 (Including Gene 57 subgroup†)	6	4	2		66.7		6	3	3		50	50.0
AV11	3	3			100.0		6		6		0	16.7
AV12 (Gene 54 subgroup†)	10	9	1		90.0		6	5		1	83.3	50.0
AV13	15	10	5		66.7		11	8	2	1	72.8	54.5
AV14	11	8	3		72.7		9	4	4	1	44.4	0.0
AV15												
AV16	3	3			100.0		2	2			100	100.0
AV17	5	2	3		40.0		3	1	2		33.3	66.7
AV18	8	7	1		87.5		8	3	5		37.5	62.5

Subgroup	Reinink and Van Rhijn (2009)					Herzig et al. (2010)††	UMD3.1 assembly annotation					Percentage of genes identified in previous annotations‡
	Total	Functional	Pseudo.	Incomplete	Percentage functional	Total	Total	Functional	Pseudo.	Incomplete	Percentage functional	
	(Number of genes)					(Number of genes)	(Number of genes)					
AV19	5	4	1		80.0		7	5	2		71.4	57.1
AV20	4	4			100.0		5	5			100	80.0
AV21	5	5			100.0		4	3	1		75	100.0
AV22	33	28	4	1	84.8		29	14	14	1	48.3	55.2
AV23	25	8	17		32.0		24	4	20		16.7	58.3
AV24	11	5	6		45.5		10	1	8	1	10	40.0
AV25	23	17	5	1	73.9		22	11	10	1	50	31.8
AV26	36	29	7		80.6		39	22	13	4	56.4	41.0
AV27	1	1			100.0		1	1			100	100.0
AV28	4	4			100.0		4	3	1		75	0.0
AV29	4	4			100.0		3	2	1		66.7	33.3
AV30												
AV31												
AV32												
AV33 (DV2)*	3	3			100.0	2	6	2	4		33.3	16.7
AV34							3		3		0	0.0
AV35	1	1			100.0		1	1			100	0.0
AV36	3	1	2		33.3		1	1			100	100.0
AV37							2		2		0	0.0
AV38	6	6			100.0		6	5		1	83.3	33.3
AV39	2	2			100.0		2	1	1		50	100.0
AV40												
AV41							1	1			100	0.0
AVX (Gene 50 subgroup†)	24	23	1		95.8		29	23	5	1	79.3	58.6
AVY (Gene 82 subgroup†)	6	6			100.0		7	7			100	57.1

Subgroup	Reinink and Van Rhijn (2009)					Herzig et al. (2010)††	UMD3.1 assembly annotation					Percentage of genes identified in previous annotations‡
	Total	Functional	Pseudo.	Incomplete	Percentage functional	Total	Total	Functional	Pseudo.	Incomplete	Percentage functional	
	(Number of genes)					(Number of genes)	(Number of genes)					
DV1	104	93	9	2	89.4	52	60	48	8	4	80	68.3
DV2							1	1			100	0.0
DV3 (DV4)**	1	1			100.0	1	1	1			100	100.0
DVb3 (DV3)**	3	2	1		66.7	1	2	2			100	50.0
DVY							1	1			100	0.0
Gene 196 subgroup	1		1		0.0							
Gene 259 subgroup	1			1	0.0							
Gene 284 subgroup	1			1	0.0							
Gene 327 subgroup	1	1			100.0							
Gene 356 subgroup	1		1		0.0							
Gene 385 subgroup	1		1		0.0							
Total	429***	336	87	6	78.3	56	371	215	138	18	58	51.8

*TRDV2 subgroup in Reinink and Van Rhijn (2009) and Herzig et al. (2010) was re-designated as TRAV33 based on orthology with the human TRAV33 subgroup

**TRDV4 subgroup in Reinink and van Rhijn (2009) and Herzig et al. (2010) was re-designated as TRDV3 based on orthology with the human TRDV4 subgroup - consequently the TRDV3 subgroup in Reinink and Van Rhijn (2009) and Herzig et al. (2010) was re-designated as TRDVb3

*** The total is 1 less than reported in Reinink and Van Rhijn (2009) as the bovine gene in bTRDV4 and huTRDV3 subgroup was the same and therefore double counted.

† Gene subgroups 50, 54, 57 and 82 identified in Reinink and Van Rhijn (2009) correspond to the TRAV subgroups X, 12, 10 and Y respectively.

†† In Herzig et al. (2010) the functionality of the annotated TRDV genes were not defined.

‡ Percentage of genes in each TRAV/TRDV subgroup annotated that had 100% nucleotide identity (from the L exon to the RS) to TRAV/TRDV gene sequences identified in Reinink and Van Rhijn (2009) and/or Herzig et al. (2010). To facilitate comparison with the genes identified by Reinink and Van Rhijn (2009) it was necessary to extend the annotations of the TRAV/TRDV genes to incorporate the entire sequence from the L-exon to the RS. The individual genes from Reinink and Van Rhijn (2009) and Herzig et al. (2010) that correspond to the annotated genes in the UMD3.1 are detailed in Additional File 1. The IMGT database incorporates the Herzig et al. (2010) TRDV1 gene annotations with some cDNA data. Only 1 TRDV1 gene derived from cDNA evidence matched an annotated TRDV1 gene (TRDV1-w was identical to TRDV1s34-2) –

therefore, with this exception the Herzig et al. (2010) and IMGT database were effectively synonymous from the perspective of identifying genes that were identical to the TRDV sequences annotated herein.

TRAJ genes

Ten of the TRAJ genes annotated herein are not included in the IMGT database (TRAJ6, 9, 13, 15, 19, 26, 36, 47, 51 and 55). The other TRAJ genes annotated here exhibit 100% nucleotide identity to the 52 TRAJ genes in the IMGT database. However, as the nomenclature of the TRAJ genes is dependent on their order there is a discrepancy between the names that individual TRAJ genes have been given in this annotation and in the IMGT database (see Additional File 1).

TRDJ and TRDD genes

The nucleotide sequences of the 5TRDD and 3TRDJ genes annotated in the UMD3.1 assembly and in the IMGT database are 100% identical.

TRAC and TRDC genes

The nucleotide sequences of the single TRAC and TRDC genes annotated in the UMD3.1 assembly and in the IMGT database are identical. However, the UMD3.1 annotation includes the non-coding 4th exon.
