# Supplementary material for: Genomic analysis offers insights into the evolution of the bovine TRA/TRD locus
Source: BMC Genomics. 2014 Nov 19;15(1):994. doi: 10.1186/1471-2164-15-994 (PMC4289303; doi:10.1186/1471-2164-15-994)
Supplement: Supplementary file 4 — Additional file 4: Annotation of the exons and RS sequences of the TRA/TRD genes. The coordinates of the (A) L-exons, V-exons and RS of each TRAV/TRDV gene, (B and C) RS and J-gene of each TRAJ and TRDJ gene, (D) RS and D-genes of each TRDD gene and (E) exons of the TRAC and TRDC gene are detailed. (PDF 2 MB) [file 12864_2014_6826_MOESM4_ESM.pdf]

A – TRAV/TRDV genes

Name	Gene group	Gene orientation	Chromosome	Start	Stop	Chromosome orientation	Functionality	Coordinates					
								L-exon		V-exon		RS	
								Start	Stop	Start	Stop	Start	Stop
DV3-a	DV3	+	Chr:10	22192121	22192742	+	Functional	22192121	22192157	22192395	22192703	22192704	22192742
DV2-a	DV2	-	Chr:10	22323161	22323689	+	Functional	22323689	22323644	22323501	22323200	22323199	22323161
DVY-a	DVY	-	Chr:10	22349850	22350390	+	Functional	22350390	22350345	22350187	22349889	22349888	22349850
DVb3-a	DVb3	-	Chr:10	22393915	22394470	+	Functional	22394470	22394416	22394246	22393954	22393953	22393915
DVb3-b	DVb3	-	Chr:10	22406746	22407310	+	Functional	22407310	22407256	22407077	22406785	22406784	22406746
AV41-a	AV41	-	Chr:10	22438607	22439182	+	Functional	22439182	22439131	22438927	22438646	22438645	22438607
AV39-a	AV39	-	Chr:10	22459631	22460166	+	Psuedogene	22460166	22460124	22459957	22459670	22459669	22459631
AV38-a	AV38	+	Chr:10	22462003	22462640	+	Functional	22462003	22462051	22462305	22462601	22462602	22462640
AV28-a	AV28	-	Chr:10	22471556	22472155	+	Functional	22472155	22472104	22471876	22471595	22471594	22471556
AV27-a	AV27	-	Chr:10	22481449	22482036	+	Functional	22482036	22481991	22481769	22481488	22481487	22481449
AV26-a	AV26	-	Chr:10	22499142	22499768	+	Incomplete	-	-	22499468	22499181	22499180	22499142
AV26-b	AV26	-	Chr:10	22504953	22505905	+	Functional	22505905	22505866	22505279	22504992	22504991	22504953
AV25-a	AV25	-	Chr:10	22512779	22513264	+	Incomplete	22513264	22513219	22512955	22512779	-	-
AV24-a	AV24	-	Chr:10	22523574	22524120	+	Psuedogene	22524120	22524066	22523900	22523613	22523612	22523574
DV1-a	DV1	-	Chr:10	22529611	22530111	+	Functional	22530111	22530063	22529947	22529650	22529649	22529611
AV26-c	AV26	-	Chr:10	22541380	22542514	+	Incomplete	22542514	22542475	22541701	22541414	22541413	22541375
AV23-a	AV23	-	Chr:10	22551389	22551927	+	Psuedogene	22551927	22551876	22551732	22551421	22551420	22551389
AV22-a	AV22	-	Chr:10	22552579	22553139	+	Psuedogene	22553139	22553088	22552895	22552618	22552617	22552579
AV26-d	AV26	-	Chr:10	22555593	22556403	+	Psuedogene	22556403	22556364	22555919	22555632	22555631	22555593
DV1-b	DV1	-	Chr:10	22570094	22570696	+	Functional	22570696	22570648	22570429	22570133	22570132	22570094
AV25-b	AV25	-	Chr:10	22576442	22577074	+	Functional	22577074	22577029	22576761	22576481	22576480	22576442
AV19-a	AV19	-	Chr:10	22585558	22586191	+	Functional	22586191	22586143	22585895	22585597	22585596	22585558
AVX-a	AVX	-	Chr:10	22587737	22588486	+	Psuedogene	22588486	22588441	22588060	22587776	22587775	22587737
AV25-c	AV25	-	Chr:10	22608673	22609302	+	Psuedogene	22609302	22609257	22608991	22608712	22608711	22608673
DV1-c	DV1	-	Chr:10	22619112	22619719	+	Functional	22619719	22619671	22619454	22619151	22619150	22619112

AV22-b	AV22	-	Chr:10	22631613	22632173	+	Functional	22632173	22632122	22631923	22631652	22631651	22631613
AV8-a	AV8	-	Chr:10	22632546	22633025	+	Psuedogene	22633025	22632980	22632866	22632585	22632584	22632546
DV1-d	DV1	-	Chr:10	22643560	22643998	+	Partial	-	-	22643890	22643599	22643598	22643560
AV22-c	AV22	-	Chr:10	22647491	22648230	+	Psuedogene	22648230	22648179	22647807	22647530	22647529	22647491
AV8-b	AV8	-	Chr:10	22648615	22649084	+	Psuedogene	22649084	22649046	22648930	22648654	22648653	22648615
DV1-e	DV1	-	Chr:10	22662964	22663571	+	Functional	22663571	22663523	22663306	22663003	22663002	22662964
AV22-d	AV22	-	Chr:10	22674452	22675008	+	Functional	22675008	22674957	22674762	22674491	22674490	22674452
AV8-c	AV8	-	Chr:10	22675380	22675860	+	Psuedogene	22675860	22675815	22675704	22675419	22675418	22675380
DV1-f	DV1	-	Chr:10	22686069	22686679	+	Functional	22686679	22686631	22686414	22686108	22686107	22686069
AV26-e	AV26	-	Chr:10	22695755	22696851	+	Functional	22696851	22696812	22696079	22695792	22695791	22695755
DV1-g	DV1	-	Chr:10	22702952	22703546	+	Functional	22703546	22703498	22703288	22702991	22702990	22702952
AVX-b	AVX	-	Chr:10	22719014	22719525	+	Functional	22719525	22719480	22719347	22719053	22719052	22719014
AV18-a	AV18	-	Chr:10	22723554	22724066	+	Psuedogene	22724066	22724021	22723880	22723593	22723592	22723554
AVY-a	AVY	-	Chr:10	22738801	22739369	+	Functional	22739369	22739318	22739118	22738840	22738839	22738801
AV8-d	AV8	-	Chr:10	22742070	22742574	+	Psuedogene	22742574	22742529	22742399	22742109	22742108	22742070
AV22-e	AV22	-	Chr:10	22754056	22754411	+	Partial	-	-	22754372	22754095	22754094	22754056
AV8-e	AV8	-	Chr:10	22754996	22755476	+	Psuedogene	22755476	22755431	22755317	22755035	22755034	22754996
DV1-h	DV1	-	Chr:10	22766038	22766636	+	Functional	22766636	22766588	22766371	22766077	22766076	22766038
AV26-f	AV26	-	Chr:10	22776924	22777730	+	Psuedogene	22777730	22777691	22777249	22776963	22776962	22776924
AV25-d	AV25	-	Chr:10	22794678	22795308	+	Psuedogene	22795308	22795263	22794998	22794717	22794716	22794678
AV24-b	AV24	-	Chr:10	22803195	22803523	+	Incomplete	-		22803521	22803234	22803233	22803195
DV1-i	DV1	-	Chr:10	22808623	22809057	+	Incomplete	22809057	22809009	22808793	22808623	-	-
AV26-g	AV26	-	Chr:10	22813972	22815090	+	Psuedogene	22815090	22815051	22814289	22814011	22814010	22813972
AV23-b	AV23	-	Chr:10	22820134	22820677	+	Psuedogene	22820677	22820626	22820480	22820172	22820171	22820134
AV22-f	AV22	-	Chr:10	22821328	22821865	+	Psuedogene	22821865	22821814	22821620	22821367	22821366	22821328
AV26-h	AV26	-	Chr:10	22827854	22828670	+	Psuedogene	22828670	22828631	22828184	22827893	22827892	22827854
DV1-j	DV1	-	Chr:10	22836189	22836789	+	Functional	22836789	22836741	22836525	22836228	22836227	22836189
AV25-e	AV25	-	Chr:10	22842847	22843474	+	Psuedogene	22843474	22843429	22843165	22842886	22842885	22842847
AV23-c	AV23	-	Chr:10	22844892	22845433	+	Psuedogene	22845433	22845381	22845235	22844924	22844923	22844892

AV22-g	AV22	-	Chr:10	22846084	22846644	+	Psuedogene	22846644	22846593	22846399	22846123	22846122	22846084
DV1-k	DV1	-	Chr:10	22856348	22856944	+	Functional	22856944	22856896	22856683	22856387	22856386	22856348
AV25-f	AV25	-	Chr:10	22863248	22863869	+	Psuedogene	22863869	22863824	22863567	22863287	22863286	22863248
AV26-i	AV26	-	Chr:10	22877059	22878204	+	Functional	22878204	22878165	22877385	22877098	22877097	22877059
DV1-l	DV1	-	Chr:10	22888343	22888919	+	Functional	22888919	22888871	22888652	22888382	22888381	22888343
AV23-d	AV23	-	Chr:10	22893540	22894082	+	Psuedogene	22894082	22894031	22893886	22893579	22893578	22893540
AV22-h	AV22	-	Chr:10	22900141	22900718	+	Functional	22900718	22900667	22900457	22900180	22900179	22900141
AV24-c	AV24	-	Chr:10	22905622	22905952	+	Partial	22905952	22905903	22905733	22905622	-	-
DV1-m	DV1	-	Chr:10	22915690	22916297	+	Functional	22916297	22916249	22916032	22915729	22915728	22915690
AV23-e	AV23	-	Chr:10	22920423	22921000	+	Psuedogene	22921000	22920949	22920804	22920461	22920460	22920423
AV39-b	AV39	-	Chr:10	22930996	22931528	+	Functional	22931528	22931486	22931319	22931035	22931034	22930996
AV38-b	AV38	-	Chr:10	22943549	22944189	+	Functional	22944189	22944141	22943887	22943588	22943587	22943549
AV38-c	AV38	-	Chr:10	22951620	22952260	+	Functional	22952260	22952212	22951958	22951659	22951658	22951620
AV38-d	AV38	-	Chr:10	22954498	22955119	+	Functional	22955119	22955071	22954839	22954537	22954536	22954498
AV37-a	AV37	-	Chr:10	22959482	22960033	+	Psuedogene	22960033	22959983	22959794	22959521	22959520	22959482
AV38-e	AV38	-	Chr:10	22988184	22988805	+	Functional	22988805	22988757	22988522	22988223	22988222	22988184
AV37-b	AV37	-	Chr:10	22993493	22994042	+	Psuedogene	22994042	22993991	22993805	22993532	22993531	22993493
AV36-a	AV36	-	Chr:10	23027669	23028262	+	Functional	23028262	23028211	23027995	23027708	23027707	23027669
AV35-a	AV35	-	Chr:10	23035476	23036105	+	Functional	23036105	23036060	23035797	23035515	23035514	23035476
AV38-f	AV38	-	Chr:10	23050097	23050686	+	Incomplete	23050686	23050638	23050384	23050097	-	-
AV33-a	AV33	-	Chr:10	23055926	23056526	+	Functional	23056526	23056478	23056263	23055965	23055964	23055926
AV29-a	AV29	-	Chr:10	23059464	23060028	+	Psuedogene	23060028	23059977	23059811	23059503	23059502	23059464
AV28-b	AV28	-	Chr:10	23065458	23066050	+	Functional	23066050	23065999	23065778	23065497	23065496	23065458
AV33-b	AV33	-	Chr:10	23069171	23069520	+	Partial	-	-	23069504	23069209	23069208	23069170
AV34-a	AV34	-	Chr:10	23076623	23077258	+	Psuedogene	23077258	23077207	23076942	23076663	23076662	23076623
AV26-j	AV26	-	Chr:10	23079664	23079809	+	Partial	-	-	23079809	23079705	23079704	23079664
AV33-c	AV33	-	Chr:10	23091059	23091650	+	Psuedogene	23091650	23091602	23091386	23091099	23091098	23091059
AV29-b	AV29	-	Chr:10	23099357	23099928	+	Functional	23099928	23099877	23099704	23099396	23099395	23099357
AV34-b	AV34	-	Chr:10	23109878	23110543	+	Psuedogene	23110543	23110492	23110199	23109917	23109916	23109878

AV8-f	AV8	-	Chr:10	23130649	23131158	+	Functional	23131158	23131113	23130982	23130688	23130687	23130649
AV26-k	AV26	-	Chr:10	23139422	23140233	+	Functional	23140233	23140194	23139748	23139461	23139460	23139422
AV25-g	AV25	-	Chr:10	23150417	23151048	+	Psuedogene	23151048	23151003	23150736	23150456	23150455	23150417
AV24-d	AV24	-	Chr:10	23157065	23157612	+	Functional	23157612	23157558	23157391	23157104	23157103	23157065
AV26-l	AV26	-	Chr:10	23164563	23165700	+	Functional	23165700	23165661	23164892	23164602	23164601	23164563
AV26-m	AV26	-	Chr:10	23172155	23173295	+	Functional	23173295	23173256	23172481	23172194	23172193	23172155
DV1-n	DV1	-	Chr:10	23181743	23182317	+	Functional	23182317	23182269	23182052	23181782	23181781	23181743
DV1-o	DV1	-	Chr:10	23196256	23196863	+	Functional	23196863	23196815	23196597	23196295	23196294	23196256
AV23-f	AV23	-	Chr:10	23202966	23203491	+	Functional	23203491	23203440	23203301	23203005	23203004	23202966
AV22-i	AV22	-	Chr:10	23207866	23208280	+	Incomplete	23208280	23208229	23208032	23207866	-	-
DV1-p	DV1	-	Chr:10	23212886	23213516	+	Functional	23213516	23213468	23213250	23212925	23212924	23212886
AV23-g	AV23	-	Chr:10	23221405	23221949	+	Functional	23221949	23221898	23221755	23221444	23221443	23221404
DV1-q	DV1	-	Chr:10	23231387	23231665	+	Incomplete	-	-	23231665	23231426	23231425	23231387
DV1-r	DV1	-	Chr:10	23240851	23241465	+	Psuedogene	23241465	23241417	23241193	23240890	23240889	23240851
AV23-h	AV23	-	Chr:10	23250003	23250548	+	Psuedogene	23250548	23250498	23250352	23250042	23250041	23250008
AV22-j	AV22	-	Chr:10	23251198	23251733	+	Psuedogene	23251733	23251682	23251488	23251237	23251236	23251198
AV26-n	AV26	-	Chr:10	23257689	23258501	+	Functional	23258501	23258462	23258015	23257728	23257727	23257689
DV1-s	DV1	-	Chr:10	23267117	23267713	+	Functional	23267713	23267665	23267453	23267156	23267155	23267117
AV25-h	AV25	-	Chr:10	23273756	23274388	+	Functional	23274388	23274343	23274075	23273795	23273794	23273756
AV23-i	AV23	-	Chr:10	23276986	23277526	+	Psuedogene	23277526	23277475	23277329	23277018	23277017	23276980
AV22-k	AV22	-	Chr:10	23278175	23278499	+	Partial	-	-	23278491	23278214	23278213	23278175
AV26-o	AV26	-	Chr:10	23281633	23282438	+	Psuedogene	23282438	23282399	23281957	23281672	23281671	23281633
DV1-t	DV1	-	Chr:10	23291265	23291858	+	Functional	23291858	23291810	23291601	23291304	23291303	23291265
AV25-i	AV25	-	Chr:10	23297598	23298216	+	Functional	23298216	23298171	23297917	23297637	23297636	23297598
AV21-a	AV21	-	Chr:10	23303762	23304334	+	Functional	23304334	23304289	23304090	23303801	23303800	23303762
AV20-a	AV20	-	Chr:10	23312082	23312623	+	Functional	23312623	23312572	23312405	23312121	23312120	23312082
AV19-b	AV19	-	Chr:10	23320406	23321109	+	Psuedogene	23321109	23321061	23320746	23320447	23320446	23320406
AVX-c	AVX	-	Chr:10	23323954	23324458	+	Functional	23324458	23324413	23324285	23323994	23323993	23323954
AVX-d	AVX	-	Chr:10	23330884	23331395	+	Functional	23331395	23331350	23331217	23330923	23330922	23330884

AVX-e	AVX	-	Chr:10	23342744	23343256	+	Functional	23343256	23343211	23343078	23342784	23342783	23342744
AV18-b	AV18	-	Chr:10	23344761	23345281	+	Functional	23345281	23345236	23345091	23344800	23344799	23344761
AV12-a	AV12	-	Chr:10	23365683	23366271	+	Functional	23366271	23366223	23366013	23365722	23365721	23365683
AV11-a	AV11	-	Chr:10	23378100	23379317	+	Psuedogene	23379317	23379266	23379073	23378139	23378138	23378100
AV10-a	AV10	-	Chr:10	23380262	23380869	+	Psuedogene	23380869	23380820	23380588	23380301	23380300	23380262
AV9-a	AV9	-	Chr:10	23385299	23385795	+	Psuedogene	23385795	23385750	23385626	23385336	23385335	23385299
AV14-a	AV14	-	Chr:10	23393563	23394110	+	Functional	23394110	23394062	23393902	23393602	23393601	23393563
AV13-a	AV13	-	Chr:10	23397807	23398356	+	Psuedogene	23398356	23398305	23398137	23397846	23397845	23397807
AVY-b	AVY	-	Chr:10	23403929	23404497	+	Functional	23404497	23404446	23404246	23403968	23403967	23403929
AV9-b	AV9	-	Chr:10	23407913	23408414	+	Psuedogene	23408414	23408369	23408246	23407955	23407954	23407913
AV14-b	AV14	-	Chr:10	23427454	23428573	+	Psuedogene	23428573	23428525	23427792	23427493	23427492	23427454
AV23-j	AV23	-	Chr:10	23447009	23447537	+	Psuedogene	23447537	23447486	23447340	23447029	23447028	23446996
AV22-l	AV22	-	Chr:10	23448188	23448748	+	Psuedogene	23448748	23448697	23448504	23448227	23448226	23448188
DV1-u	DV1	-	Chr:10	23459145	23459737	+	Functional	23459737	23459689	23459480	23459184	23459183	23459145
AV25-j	AV25	-	Chr:10	23465670	23466300	+	Functional	23466300	23466255	23465990	23465709	23465708	23465670
AV23-k	AV23	-	Chr:10	23468888	23469434	+	Psuedogene	23469434	23469383	23469237	23468927	23468926	23468887
AV22-m	AV22	-	Chr:10	23470079	23470639	+	Psuedogene	23470639	23470588	23470395	23470118	23470117	23470079
AV26-p	AV26	-	Chr:10	23476595	23477404	+	Functional	23477404	23477365	23476918	23476634	23476633	23476595
DV1-v	DV1	-	Chr:10	23486840	23487437	+	Psuedogene	23487437	23487389	23487177	23486879	23486878	23486840
AV25-k	AV25	-	Chr:10	23493599	23494233	+	Functional	23494233	23494188	23493919	23493638	23493637	23493599
AV23-l	AV23	-	Chr:10	23496909	23497450	+	Psuedogene	23497450	23497399	23497253	23496941	23496940	23496909
AV22-n	AV22	-	Chr:10	23498102	23498662	+	Functional	23498662	23498611	23498418	23498141	23498140	23498102
AV26-q	AV26	-	Chr:10	23501110	23501920	+	Psuedogene	23501920	23501881	23501436	23501149	23501148	23501110
DV1-w	DV1	-	Chr:10	23511326	23511929	+	Functional	23511929	23511881	23511661	23511365	23511364	23511326
AV25-l	AV25	-	Chr:10	23517595	23518218	+	Functional	23518218	23518173	23517915	23517634	23517633	23517595
AV26-r	AV26	-	Chr:10	23524272	23525404	+	Functional	23525404	23525365	23524598	23524311	23524310	23524272
DV1-x	DV1	-	Chr:10	23535596	23536211	+	Functional	23536211	23536163	23535938	23535635	23535634	23535596
AV23-m	AV23	-	Chr:10	23540770	23541303	+	Psuedogene	23541303	23541252	23541108	23540809	23540808	23540770
AV22-o	AV22	-	Chr:10	23549339	23549910	+	Psuedogene	23549910	23549859	23549654	23549378	23549377	23549339

AV24-e	AV24	-	Chr:10	23554690	23555231	+	Psuedogene	23555231	23555177	23555007	23554729	23554728	23554690
DV1-y	DV1	-	Chr:10	23567044	23567649	+	Functional	23567649	23567601	23567386	23567083	23567082	23567044
AV23-n	AV23	-	Chr:10	23571788	23572347	+	Psuedogene	23572347	23572296	23572151	23571826	23571825	23571788
AV22-p	AV22	-	Chr:10	23577090	23577668	+	Psuedogene	23577668	23577617	23577406	23577129	23577128	23577090
AV24-f	AV24	-	Chr:10	23581698	23582251	+	Partial	-	-	23582024	23581737	23581736	23581698
DV1-z	DV1	-	Chr:10	23586938	23587548	+	Functional	23587548	23587500	23587279	23586977	23586976	23586938
AV22-q	AV22	-	Chr:10	23592483	23593043	+	Functional	23593043	23592992	23592799	23592522	23592521	23592483
AV26-s	AV26	-	Chr:10	23595541	23596350	+	Functional	23596350	23596311	23595867	23595580	23595579	23595541
AV25-m	AV25	-	Chr:10	23605890	23606523	+	Functional	23606523	23606478	23606209	23605929	23605928	23605890
DV1-aa	DV1	-	Chr:10	23618035	23618641	+	Functional	23618641	23618593	23618377	23618074	23618073	23618035
AV23-o	AV23	-	Chr:10	23624547	23625092	+	Functional	23625092	23625041	23624897	23624586	23624585	23624547
AV22-r	AV22	-	Chr:10	23631953	23632510	+	Psuedogene	23632510	23632459	23632269	23631992	23631991	23631953
AV8-g	AV8	-	Chr:10	23636381	23636869	+	Functional	23636869	23636824	23636714	23636420	23636419	23636381
AV21-b	AV21	-	Chr:10	23639854	23640425	+	Functional	23640425	23640380	23640182	23639893	23639892	23639854
AV20-b	AV20	-	Chr:10	23647889	23648424	+	Functional	23648424	23648373	23648206	23647928	23647927	23647889
AV6-a	AV6	-	Chr:10	23683754	23684284	+	Incomplete	23684284	23684235	23684055	23683771	23683770	23683754
DV1-ab	DV1	-	Chr:10	23720626	23721233	+	Functional	23721233	23721185	23720968	23720665	23720664	23720626
AV22-s	AV22	-	Chr:10	23727226	23727786	+	Functional	23727786	23727735	23727542	23727265	23727264	23727226
AV26-t	AV26	-	Chr:10	23730243	23731051	+	Functional	23731051	23731012	23730569	23730282	23730281	23730243
AV25-n	AV25	-	Chr:10	23737275	23737909	+	Psuedogene	23737909	23737864	23737595	23737314	23737313	23737275
DV1-ac	DV1	-	Chr:10	23744316	23744921	+	Functional	23744921	23744873	23744658	23744355	23744354	23744316
AV19-c	AV19	-	Chr:10	23748128	23748758	+	Functional	23748758	23748710	23748465	23748167	23748166	23748128
AVX-f	AVX	-	Chr:10	23750612	23751371	+	Functional	23751371	23751326	23750945	23750651	23750650	23750612
AVX-g	AVX	-	Chr:10	23767027	23767538	+	Functional	23767538	23767493	23767360	23767066	23767065	23767027
AVX-h	AVX	-	Chr:10	23773137	23773646	+	Functional	23773646	23773601	23773470	23773176	23773175	23773137
AVX-i	AVX	-	Chr:10	23778826	23779335	+	Functional	23779335	23779290	23779159	23778865	23778864	23778826
AV18-c	AV18	-	Chr:10	23783361	23783874	+	Psuedogene	23783874	23783829	23783688	23783400	23783399	23783361
AV17-a	AV17	-	Chr:10	23794652	23795442	+	Functional	23795442	23795391	23794978	23794691	23794690	23794652
AV16-a	AV16	-	Chr:10	23801677	23802159	+	Functional	23802159	23802114	23801998	23801716	23801715	23801677

AV14-c	AV14	-	Chr:10	23823839	23824965	+	Psuedogene	23824965	23824917	23824174	23823878	23823877	23823839
AV13-b	AV13	-	Chr:10	23829114	23829653	+	Functional	23829653	23829602	23829440	23829153	23829152	23829114
AV12-b	AV12	-	Chr:10	23842486	23843073	+	Functional	23843073	23843025	23842815	23842525	23842524	23842486
AV11-b	AV11	-	Chr:10	23852978	23853549	+	Psuedogene	23853549	23853498	23853306	23853017	23853016	23852978
AV10-b	AV10	-	Chr:10	23854487	23855099	+	Functional	23855099	23855048	23854816	23854526	23854525	23854487
AV9-c	AV9	-	Chr:10	23862145	23862643	+	Psuedogene	23862643	23862597	23862473	23862182	23862181	23862145
AV14-d	AV14	-	Chr:10	23871677	23872220	+	Psuedogene	23872220	23872172	23872014	23871715	23871714	23871677
AV13-c	AV13	-	Chr:10	23875405	23875955	+	Pseudogene	23875955	23875904	23875736	23875444	23875443	23875405
AVY-c	AVY	-	Chr:10	23879813	23880370	+	Functional	23880370	23880319	23880130	23879852	23879851	23879813
AV20-c	AV20	+	Chr:10	23888939	23889462	+	Functional	23888939	23888990	23889145	23889423	23889424	23889462
AV26-u	AV26	+	Chr:10	23900317	23901458	+	Functional	23900317	23900356	23901132	23901419	23901420	23901458
AV23-p	AV23	+	Chr:10	23905670	23905948	+	Partial	-	-	23905948	23905688	-	-
DV1-ad	DV1	+	Chr:10	23910774	23911289	+	Pseudogene	23910774	23910822	23910938	23911252	23911253	23911289
DV1-ae	DV1	+	Chr:10	23917231	23917831	+	Functional	23917231	23917279	23917495	23917792	23917793	23917831
AV24-g	AV24	+	Chr:10	23923492	23924039	+	Psuedogene	23923492	23923546	23923713	23924000	23924001	23924039
AV25-o	AV25	+	Chr:10	23927453	23928089	+	Psuedogene	23927453	23927498	23927770	23928050	23928051	23928089
DV1-af	DV1	+	Chr:10	23933924	23934520	+	Functional	23933924	23933972	23934185	23934481	23934482	23934520
AV26-v	AV26	+	Chr:10	23952004	23953132	+	Psuedogene	23952004	23952043	23952808	23953093	23953094	23953132
DV1-ag	DV1	+	Chr:10	23959743	23960346	+	Functional	23959743	23959791	23960010	23960307	23960308	23960346
AV24-h	AV24	+	Chr:10	23965646	23966191	+	Psuedogene	23965646	23965700	23965865	23966152	23966153	23966191
AV25-p	AV25	+	Chr:10	23971979	23972611	+	Functional	23971979	23972024	23972292	23972572	23972573	23972611
DV1-ah	DV1	+	Chr:10	23978150	23978745	+	Functional	23978150	23978198	23978409	23978706	23978707	23978745
AV26-w	AV26	+	Chr:10	23984689	23985499	+	Functional	23984689	23984728	23985173	23985460	23985461	23985499
DV1-ai	DV1	+	Chr:10	23995847	23996448	+	Functional	23995847	23995895	23996112	23996409	23996410	23996448
AV8-h	AV8	+	Chr:10	24007152	24007631	+	Psuedogene	24007152	24007197	24007308	24007592	24007593	24007631
AV22-t	AV22	+	Chr:10	24008004	24008570	+	Functional	24008004	24008055	24008254	24008531	24008532	24008570
AV26-x	AV26	-	Chr:10	24026000	24027044	+	Incomplete	-		24026326	24026039	24026038	24026000
DV1-aj	DV1	-	Chr:10	24028336	24028836	+	Functional	24028836	24028788	24028672	24028375	24028374	24028336
AV26-y	AV26	-	Chr:10	24040176	24041310	+	Psuedogene	24041310	24041271	24040497	24040215	24040214	24040176

AVY-d	AVY	+	Chr:10	24049503	24050060	+	Functional	24049503	24049554	24049743	24050021	24050022	24050060
AV9-d	AV9	-	Chr:10	24054652	24054988	+	Incomplete	24054988	24054943	24054815	24054652	-	-
AV8-i	AV8	-	Chr:10	24062432	24062915	+	Functional	24062915	24062870	24062765	24062471	24062470	24062432
AV26-z	AV26	-	Chr:10	24080740	24081553	+	Psuedogene	24081553	24081514	24081065	24080779	24080778	24080740
AV25-q	AV25	-	Chr:10	24091660	24092289	+	Psuedogene	24092289	24092244	24091979	24091699	24091698	24091660
DV1-ak	DV1	-	Chr:10	24099007	24099612	+	Functional	24099612	24099564	24099349	24099046	24099045	24099007
AV23-q	AV23	-	Chr:10	24105088	24105633	+	Psuedogene	24105633	24105582	24105438	24105127	24105126	24105088
AV19-d	AV19	-	Chr:10	24109596	24110232	+	Functional	24110232	24110184	24109935	24109637	24109636	24109596
AVX-j	AVX	-	Chr:10	24111782	24112287	+	Functional	24112287	24112242	24112116	24111822	24111821	24111782
AV22-u	AV22	-	Chr:10	24115721	24116278	+	Functional	24116278	24116227	24116037	24115760	24115759	24115721
AV8-j	AV8	-	Chr:10	24120142	24120630	+	Functional	24120630	24120585	24120475	24120181	24120180	24120142
AV21-c	AV21	-	Chr:10	24128548	24129119	+	Functional	24129119	24129074	24128876	24128587	24128586	24128548
AV20-d	AV20	-	Chr:10	24132045	24132580	+	Functional	24132580	24132529	24132362	24132084	24132083	24132045
AV19-e	AV19	-	Chr:10	24140329	24140985	+	Psuedogene	24140985	24140937	24140665	24140368	24140367	24140329
AVX-k	AVX	-	Chr:10	24142546	24143051	+	Functional	24143051	24143006	24142880	24142586	24142585	24142546
AVX-l	AVX	-	Chr:10	24149270	24149779	+	Functional	24149779	24149734	24149603	24149309	24149308	24149270
AVX-m	AVX	-	Chr:10	24156559	24157069	+	Functional	24157069	24157024	24156891	24156597	24156596	24156559
AVX-n	AVX	-	Chr:10	24175745	24176256	+	Functional	24176256	24176211	24176078	24175784	24175783	24175745
AVX-o	AVX	-	Chr:10	24197766	24198277	+	Functional	24198277	24198232	24198099	24197805	24197804	24197766
AV18-d	AV18	-	Chr:10	24201940	24202460	+	Functional	24202460	24202415	24202267	24201979	24201978	24201940
AV12-c	AV12	-	Chr:10	24218758	24219345	+	Functional	24219345	24219297	24219087	24218797	24218796	24218758
AV11-c	AV11	-	Chr:10	24231371	24231941	+	Psuedogene	24231941	24231892	24231699	24231410	24231409	24231371
AV10-c	AV10	-	Chr:10	24232705	24233186	+	Partial	24233186	24233135	24232905	24232705	-	-
AV13-d	AV13	-	Chr:10	24238205	24238751	+	Functional	24238751	24238700	24238531	24238244	24238243	24238205
AV9-e	AV9	-	Chr:10	24242881	24243387	+	Psuedogene	24243387	24243341	24243213	24242922	24242921	24242881
AVX-p	AVX	-	Chr:10	24262149	24262659	+	Functional	24262659	24262614	24262482	24262188	24262187	24262149
AV18-e	AV18	-	Chr:10	24266301	24266821	+	Psuedogene	24266821	24266776	24266630	24266339	24266338	24266301
AV12-d	AV12	-	Chr:10	24283143	24283728	+	Functional	24283728	24283680	24283473	24283182	24283181	24283143
AV11-e	AV11	-	Chr:10	24292968	24293540	+	Psuedogene	24293540	24293489	24293294	24293005	24293004	24292968

AV10-d	AV10	-	Chr:10	24294490	24295098	+	Functional	24295098	24295047	24294816	24294529	24294528	24294490
AV9-f	AV9	-	Chr:10	24299528	24300026	+	Psuedogene	24300026	24299981	24299857	24299567	24299566	24299528
AV14-e	AV14	-	Chr:10	24309115	24309661	+	Functional	24309661	24309613	24309453	24309154	24309153	24309115
AV13-e	AV13	-	Chr:10	24312820	24313369	+	Functional	24313369	24313318	24313149	24312859	24312858	24312820
AVY-e	AVY	-	Chr:10	24317208	24317765	+	Functional	24317765	24317714	24317525	24317247	24317246	24317208
AV6-b	AV6	-	Chr:10	24336941	24337171	+	Partial	-	-	24337171	24336980	24336979	24336941
AV5-a	AV5	-	Chr:10	24346160	24346711	+	Functional	24346711	24346660	24346489	24346199	24346198	24346160
AV4-a	AV4	-	Chr:10	24357279	24358153	+	Functional	24358153	24358114	24357605	24357318	24357317	24357279
AV14-f	AV14	-	Chr:10	24448557	24449660	+	Psuedogene	24449660	24449612	24448896	24448596	24448595	24448557
AV13-f	AV13	-	Chr:10	24453902	24454441	+	Functional	24454441	24454390	24454228	24453941	24453940	24453902
AV9-g	AV9	-	Chr:10	24457553	24458060	+	Psuedogene	24458060	24458015	24457886	24457594	24457593	24457553
DV1-al	DV1	-	Chr:10	24465666	24466273	+	Functional	24466273	24466225	24466008	24465705	24465704	24465666
AVY-f	AVY	-	Chr:10	24485081	24485638	+	Functional	24485638	24485587	24485398	24485120	24485119	24485081
AV9-h	AV9	-	Chr:10	24490204	24490695	+	Psuedogene	24490695	24490658	24490534	24490243	24490242	24490204
DV1-am	DV1	-	Chr:10	24494855	24495456	+	Functional	24495456	24495408	24495191	24494894	24494893	24494855
AV8-k	AV8	+	Chr:10	24502277	24502774	+	Psuedogene	24502277	24502322	24502445	24502735	24502736	24502774
DV1-an	DV1	-	Chr:10	24511917	24512517	+	Functional	24512517	24512469	24512253	24511956	24511955	24511917
AV8-l	AV8	-	Chr:10	24519435	24519944	+	Functional	24519944	24519899	24519768	24519474	24519473	24519435
AV26-aa	AV26	-	Chr:10	24532911	24533716	+	Functional	24533716	24533677	24533237	24532950	24532949	24532911
DV1-ao	DV1	-	Chr:10	24541572	24542167	+	Functional	24542167	24542119	24541908	24541611	24541610	24541572
AV25-r	AV25	-	Chr:10	24547576	24548208	+	Functional	24548208	24548163	24547895	24547615	24547614	24547576
AV24-i	AV24	-	Chr:10	24554392	24554937	+	Psuedogene	24554937	24554883	24554718	24554431	24554430	24554392
DV1-ap	DV1	-	Chr:10	24560197	24560798	+	Psuedogene	24560798	24560750	24560532	24560236	24560235	24560197
AV26-ab	AV26	-	Chr:10	24565384	24566520	+	Psuedogene	24566520	24566481	24565710	24565423	24565422	24565384
DV1-aq	DV1	-	Chr:10	24574066	24574566	+	Functional	24574566	24574518	24574402	24574105	24574104	24574066
AV26-ac	AV26	-	Chr:10	24585426	24586565	+	Functional	24586565	24586526	24585752	24585465	24585464	24585426
DV1-ar	DV1	-	Chr:10	24600541	24601116	+	Functional	24601116	24601068	24600850	24600580	24600579	24600541
DV1-as	DV1	-	Chr:10	24617556	24618165	+	Functional	24618165	24618117	24617897	24617595	24617594	24617556
AV23-r	AV23	-	Chr:10	24623322	24623700	+	Partial	24623700	24623649	24623509	24623322	-	-

AV22-v	AV22	-	Chr:10	24628717	24629272	+	Psuedogene	24629272	24629222	24629029	24628756	24628755	24628717
DV1-at	DV1	-	Chr:10	24634116	24634726	+	Psuedogene	24634726	24634678	24634458	24634155	24634154	24634116
AV23-s	AV23	-	Chr:10	24643687	24644235	+	Psuedogene	24644235	24644183	24644037	24643726	24643725	24643694
AV22-w	AV22	-	Chr:10	24646142	24646699	+	Functional	24646699	24646648	24646458	24646181	24646180	24646142
AV26-ad	AV26	-	Chr:10	24649143	24649954	+	Functional	24649954	24649915	24649469	24649182	24649181	24649143
DV1-au	DV1	-	Chr:10	24657755	24658363	+	Functional	24658363	24658315	24658090	24657794	24657793	24657755
AV25-s	AV25	-	Chr:10	24664103	24664725	+	Psuedogene	24664725	24664680	24664423	24664142	24664141	24664103
DV1-av	DV1	-	Chr:10	24674694	24675301	+	Psuedogene	24675301	24675253	24675035	24674733	24674732	24674694
AV22-x	AV22	-	Chr:10	24680216	24680776	+	Functional	24680776	24680725	24680532	24680255	24680254	24680216
AV26-ae	AV26	-	Chr:10	24683272	24684086	+	Functional	24684086	24684047	24683598	24683311	24683310	24683272
AV25-t	AV25	-	Chr:10	24696506	24697488	+	Functional	24697488	24697443	24696825	24696545	24696544	24696506
DV1-aw	DV1	-	Chr:10	24703626	24704231	+	Functional	24704231	24704183	24703968	24703665	24703664	24703626
AV23-t	AV23	-	Chr:10	24709718	24710263	+	Psuedogene	24710263	24710212	24710068	24709757	24709756	24709718
AVX-q	AVX	-	Chr:10	24734925	24735436	+	Functional	24735436	24735391	24735258	24734964	24734963	24734925
AVX-r	AVX	-	Chr:10	24759310	24759821	+	Functional	24759821	24759776	24759643	24759349	24759348	24759310
AVX-s	AVX	-	Chr:10	24764887	24765398	+	Functional	24765398	24765353	24765220	24764926	24764925	24764887
AV18-f	AV18	-	Chr:10	24770367	24770888	+	Psuedogene	24770888	24770843	24770697	24770406	24770405	24770367
AV17-b	AV17	+	Chr:10	24781716	24782504	+	Psuedogene	24781716	24781767	24782180	24782464	24782465	24782503
AV14-g	AV14	-	Chr:10	24801831	24802961	+	Functional	24802961	24802913	24802170	24801870	24801869	24801831
AV12-e	AV12	-	Chr:10	24807295	24807750	+	Incomplete	24807750	24807702	24807491	24807295	-	-
AV11-d	AV11	-	Chr:10	24817354	24817927	+	Psuedogene	24817927	24817876	24817682	24817393	24817392	24817354
AV10-e	AV10	-	Chr:10	24818880	24819489	+	Functional	24819489	24819438	24819209	24818919	24818918	24818880
AV9-i	AV9	-	Chr:10	24823985	24824480	+	Psuedogene	24824480	24824428	24824304	24824024	24824023	24823985
AV14-h	AV14	-	Chr:10	24832945	24833492	+	Functional	24833492	24833444	24833284	24832984	24832983	24832945
AV13-g	AV13	-	Chr:10	24836681	24837226	+	Functional	24837226	24837175	24837010	24836720	24836719	24836681
AVY-g	AVY	-	Chr:10	24838384	24838941	+	Functional	24838941	24838890	24838701	24838423	24838422	24838384
AV24-j	AV24	-	Chr:10	24847585	24848131	+	Psuedogene	24848131	24848077	24847911	24847624	24847623	24847585
DV1-ax	DV1	-	Chr:10	24852524	24853126	+	Functional	24853126	24853078	24852860	24852563	24852562	24852524
AV26-af	AV26	-	Chr:10	24857714	24858850	+	Psuedogene	24858850	24858811	24858039	24857753	24857752	24857714

DV1-ay	DV1	-	Chr:10	24865173	24865673	+	Functional	24865673	24865625	24865509	24865212	24865211	24865173
AV26-ag	AV26	-	Chr:10	24875657	24876793	+	Functional	24876793	24876754	24875983	24875696	24875695	24875657
AV6-c	AV6	+	Chr:10	24885032	24885584	+	Psuedogene	24885032	24885081	24885261	24885545	24885546	24885584
AV6-d	AV6	+	Chr:10	24885884	24886436	+	Psuedogene	24885884	24885933	24886113	24886397	24886398	24886436
AV5-b	AV5	-	Chr:10	24902611	24903163	+	Functional	24903163	24903112	24902940	24902650	24902649	24902611
AV13-h	AV13	+	Chr:10	24909812	24910351	+	Functional	24909812	24909863	24910025	24910312	24910313	24910351
DV1-az	DV1	-	Chr:10	24920012	24920168	+	Incomplete	-	-	24920168	24920051	24920050	24920012
DV1-ba	DV1	-	Chr:10	24932109	24932747	+	Functional	24932747	24932699	24932482	24932148	24932147	24932109
AV4-b	AV4	-	Chr:10	24948745	24949619	+	Functional	24949619	24949580	24949071	24948784	24948783	24948745
AV23-u	AV23	-	Chr:10	24959516	24960058	+	Psuedogene	24960058	24960007	24959864	24959555	24959554	24959516
AV26-ah	AV26	-	Chr:10	24967750	24968891	+	Functional	24968891	24968852	24968076	24967789	24967788	24967750
DV1-bb	DV1	-	Chr:10	24992843	24993451	+	Functional	24993451	24993403	24993184	24992882	24992881	24992843
AV23-v	AV23	-	Chr:10	24997916	24998457	+	Psuedogene	24998457	24998406	24998266	24997955	24997954	24997916
AV22-y	AV22	-	Chr:10	25003151	25003592	+	Partial	25003592	25003542	25003348	25003151	-	-
AVX-t	AVX	-	Chr:10	25018494	25019002	+	Psuedogene	25019002	25018961	25018828	25018534	25018533	25018494
AV16-b	AV16	+	Chr:10	25034456	25034937	+	Functional	25034456	25034501	25034616	25034898	25034899	25034937
AV17-c	AV17	+	Chr:10	25040218	25041005	+	Psuedogene	25040218	25040269	25040682	25040966	25040967	25041005
AV18-g	AV18	+	Chr:10	25053543	25054057	+	Psuedogene	25053543	25053588	25053730	25054018	25054019	25054057
AVX-u	AVX	+	Chr:10	25058084	25058382	+	Psuedogene	25058084	25058129	25058263	25058557	25058558	25058597
AVX-u	AVX	+	Chr:10	25058084	25058382	+	Incomplete	25058084	25058129	25058262	25058382	-	-
AV18-h	AV18	+	Chr:10	25059685	25060202	+	Functional	25059685	25059730	25059872	25060163	25060164	25060202
AVX-w	AVX	+	Chr:10	25069836	25070347	+	Functional	25069836	25069881	25070014	25070308	25070309	25070347
AV26-ai	AV26	+	Chr:10	25080384	25081196	+	Functional	25080384	25080423	25080870	25081157	25081158	25081196
AVX-x	AVX	+	Chr:10	25090080	25090592	+	Functional	25090080	25090125	25090258	25090552	25090553	25090592
AVX-y	AVX	+	Chr:10	25109417	25110166	+	Psuedogene	25109417	25109462	25109843	25110127	25110128	25110166
AV19-f	AV19	+	Chr:10	25111710	25112343	+	Functional	25111710	25111758	25112006	25112304	25112305	25112343
AV25-u	AV25	+	Chr:10	25120865	25121495	+	Psuedogene	25120865	25120910	25121176	25121456	25121457	25121495
DV1-bc	DV1	+	Chr:10	25127239	25127837	+	Functional	25127239	25127287	25127502	25127798	25127799	25127837
AV26-aj	AV26	+	Chr:10	25136250	25137062	+	Functional	25136250	25136289	25136736	25137023	25137024	25137062

AV22-z	AV22	+	Chr:10	25139502	25140062	+	Functional	25139502	25139553	25139746	25140023	25140024	25140062
AV23-w	AV23	+	Chr:10	25140700	25141240	+	Psuedogene	25140700	25140751	25140897	25141208	25141209	25141240
AVX-z	AVX	-	Chr:10	25161604	25162115	+	Functional	25162115	25162070	25161937	25161643	25161642	25161604
AVX-aa	AVX	-	Chr:10	25168344	25168853	+	Functional	25168853	25168808	25168677	25168383	25168382	25168344
DV1-bd	DV1	+	Chr:10	25173058	25173277	+	Incomplete	-	-	25173058	25173238	25173239	25173277
AV12-f	AV12	-	Chr:10	25193590	25194177	+	Functional	25194177	25194129	25193919	25193629	25193628	25193590
AV11-f	AV11	-	Chr:10	25206225	25206795	+	Psuedogene	25206795	25206746	25206553	25206264	25206263	25206225
AV10-f	AV10	-	Chr:10	25207560	25208042	+	Partial	25208042	25207991	25207760	25207560	-	-
AV13-i	AV13	-	Chr:10	25213065	25213263	+	Incomplete	-		25213263	25213044	25213043	25213005
AV13-j	AV13	-	Chr:10	25216585	25217131	+	Functional	25217131	25217080	25216911	25216624	25216623	25216585
AV9-j	AV9	-	Chr:10	25221236	25221738	+	Psuedogene	25221738	25221686	25221562	25221282	25221281	25221245
AV4-c	AV4	-	Chr:10	25230662	25231525	+	Psuedogene	25231525	25231486	25230988	25230701	25230700	25230662
AV22-aa	AV22	-	Chr:10	25244820	25245386	+	Functional	25245386	25245335	25245136	25244859	25244858	25244820
AV8-m	AV8	-	Chr:10	25245760	25246234	+	Psuedogene	25246234	25246189	25246079	25245798	25245797	25245759
DV1-be	DV1	-	Chr:10	25255950	25256544	+	Functional	25256544	25256496	25256280	25255989	25255988	25255950
AV26-ak	AV26	-	Chr:10	25265502	25266354	+	Incomplete	25266354	25266315	25265828	25265541	25265540	25265502
DV1-bf	DV1	-	Chr:10	25296972	25297580	+	Functional	25297580	25297532	25297314	25297011	25297010	25296972
AV22-ab	AV22	-	Chr:10	25303580	25304137	+	Functional	25304137	25304086	25303896	25303619	25303618	25303580
AV26-al	AV26	-	Chr:10	25306592	25307400	+	Functional	25307400	25307361	25306918	25306631	25306630	25306592
AV25-v	AV25	-	Chr:10	25320268	25320901	+	Functional	25320901	25320856	25320588	25320307	25320306	25320268
DV1-bg	DV1	-	Chr:10	25341876	25342481	+	Functional	25342481	25342433	25342218	25341915	25341914	25341876
AV23-x	AV23	-	Chr:10	25348338	25348883	+	Functional	25348883	25348832	25348688	25348377	25348376	25348338
AV22-ac	AV22	-	Chr:10	25357331	25357888	+	Functional	25357888	25357837	25357647	25357370	25357369	25357331
AV8-n	AV8	-	Chr:10	25361725	25362213	+	Functional	25362213	25362168	25362058	25361764	25361763	25361725
AV21-d	AV21	-	Chr:10	25370239	25370812	+	Psuedogene	25370812	25370767	25370567	25370278	25370277	25370239
AV20-e	AV20	-	Chr:10	25378267	25378800	+	Functional	25378800	25378749	25378584	25378306	25378305	25378267
AV19-g	AV19	-	Chr:10	25386522	25387169	+	Functional	25387169	25387121	25386859	25386561	25386560	25386522
AVX-ab	AVX	-	Chr:10	25388718	25389222	+	Functional	25389222	25389177	25389051	25388757	25388756	25388718
AVX-ac	AVX	-	Chr:10	25397210	25397717	+	Psuedogene	25397717	25397672	25397543	25397249	25397248	25397210

AV3-a	AV3	-	Chr:10	25416553	25417024	+	Functional	25417024	25416979	25416886	25416592	25416591	25416553
AV2-a	AV2	-	Chr:10	25421891	25422379	+	Functional	25422379	25422316	25422203	25421930	25421929	25421891
AV3-b	AV3	-	Chr:10	25440313	25440784	+	Functional	25440784	25440739	25440646	25440352	25440351	25440313
AV2-b	AV2	-	Chr:10	25449384	25449872	+	Functional	25449872	25449809	25449696	25449423	25449422	25449384
AV3-c	AV3	-	Chr:10	25467843	25468314	+	Functional	25468314	25468269	25468176	25467882	25467881	25467843
AV2-c	AV2	-	Chr:10	25474836	25475326	+	Functional	25475326	25475263	25475148	25474875	25474874	25474836
AV3-d	AV3	-	Chr:10	25489825	25490291	+	Functional	25490291	25490246	25490158	25489864	25489863	25489825
AV2-d	AV2	-	Chr:10	25496856	25497344	+	Psuedogene	25497344	25497281	25497166	25496895	25496894	25496856
AV3-e	AV3	-	Chr:10	25508599	25509070	+	Functional	25509070	25509025	25508932	25508638	25508637	25508599
AV2-e	AV2	-	Chr:10	25515891	25516385	+	Psuedogene	25516385	25516322	25516202	25515930	25515929	25515891
AV3-f	AV3	-	Chr:10	25530576	25531042	+	Functional	25531042	25530997	25530909	25530615	25530614	25530576
AV2-f	AV2	-	Chr:10	25537400	25537883	+	Functional	25537883	25537820	25537712	25537439	25537438	25537400
AV3-g	AV3	-	Chr:10	25547747	25548213	+	Functional	25548213	25548168	25548080	25547786	25547785	25547747
AV2-g	AV2	-	Chr:10	25554768	25555258	+	Functional	25555258	25555195	25555080	25554807	25554806	25554768
AV1-a	AV1	-	Chr:10	25628625	25629292	+	Functional	25629292	25629250	25628946	25628664	25628663	25628625
AV9-k	AV9	+	Chr:10	60202862	60203365	+	Functional	60202862	60202907	60203036	60203327	60203328	60203365
AV13-k	AV13	+	Chr:10	60207211	60207750	+	Functional	60207211	60207262	60207424	60207711	60207712	60207750
AV14-i	AV14	+	Chr:10	60211894	60212611	+	Incomplete	60211894	60211942	-	-	-	-
DV1-bh	DV1	-	Chr:9	71355801	71356402	+	Orphon	71356402	71356354	71356137	71355840	71355839	71355801
AV26-am	AV26	-	DAAA02066600.1	29241	30055	+	Psuedogene	30055	30016	29564	29280	29279	29241
AV28-c	AV28	-	DAAA02066600.1	11292	11883	+	Functional	11883	11832	11612	11331	11330	11292
AV33-d	AV33	-	DAAA02066600.1	1506	2062	+	Functional	2062	2014	1843	1545	1544	1506
AV29-c	AV29	-	DAAA02066600.1	4996	5567	+	Functional	5567	5516	5344	5035	5034	4996
AV28-d	AV28	-	DAAA02066600.1	12370	12491	+	Partial	-	-	12491	12370	-	-
AV33-e	AV33	-	DAAA02066600.1	14997	15344	+	Partial	-	-	15328	15036	15035	14997
AV34-c	AV34	-	DAAA02066600.1	25454	26115	+	Psuedogene	26115	26064	25773	25494	25493	25454
AV33-f	AV33	-	DAAA02066600.1	48475	49067	+	Psuedogene	49067	49019	48803	48515	48514	48475

B - TRAJ genes

Name	Gene group	Gene ori.	Chromosome	Start	Stop	Chrom. ori.	Functionality	Coordinates						Reading frame
								RS		J exon		Splice site		
								Start	Stop	Start	Stop	Start	Stop	
bTRAJ1	TRAJ	-	Chr:10	22115247	22115341	+	Functional	22115341	22115314	22115313	22115252	22115251	22115247	2
bTRAJ2	TRAJ	-	Chr:10	22116214	22116312	+	Functional	22116312	22116285	22116284	22116219	22116218	22116214	3
bTRAJ3	TRAJ	-	Chr:10	22116760	22116851	+	Functional	22116851	22116824	22116823	22116765	22116764	22116760	2
bTRAJ4	TRAJ	-	Chr:10	22117730	22117813	+	Pseudogene	22117813	22117786	22117785	22117735	22117734	22117730	3
bTRAJ5	TRAJ	-	Chr:10	22120919	22121011	+	Functional	22121011	22120984	22120983	22120924	22120923	22120919	3
bTRAJ6	TRAJ	-	Chr:10	22122107	22122201	+	Functional	22122201	22122174	22122173	22122112	22122111	22122107	2
bTRAJ7	TRAJ	-	Chr:10	22122688	22122776	+	Functional	22122776	22122749	22122748	22122693	22122692	22122688	2
bTRAJ8-1	TRAJ	-	Chr:10	22124233	22124325	+	Functional	22124325	22124298	22124297	22124238	22124237	22124233	3
bTRAJ9	TRAJ	-	Chr:10	22124822	22124915	+	Functional	22124915	22124888	22124887	22124827	22124826	22124822	3
bTRAJ8-2	TRAJ	-	Chr:10	22127021	22127113	+	Functional	22127113	22127086	22127085	22127026	22127025	22127021	3
bTRAJ10	TRAJ	-	Chr:10	22127480	22127575	+	Functional	22127575	22127548	22127547	22127485	22127484	22127480	1
bTRAJ11	TRAJ	-	Chr:10	22128491	22128583	+	Functional	22128583	22128556	22128555	22128496	22128495	22128491	3
bTRAJ12	TRAJ	-	Chr:10	22129052	22129144	+	Functional	22129144	22129117	22129116	22129057	22129056	22129052	3
bTRAJ13	TRAJ	-	Chr:10	22129908	22130003	+	Pseudogene	22130003	22129976	22129975	22129913	22129912	22129908	3
bTRAJ14	TRAJ	-	Chr:10	22130630	22130714	+	ORF	22130714	22130687	22130686	22130635	22130634	22130630	1
bTRAJ15	TRAJ	-	Chr:10	22131522	22131614	+	Functional	22131614	22131587	22131586	22131527	22131526	22131522	3
bTRAJ16	TRAJ	-	Chr:10	22132617	22132710	+	Functional	22132710	22132683	22132682	22132622	22132621	22132617	1
bTRAJ17	TRAJ	-	Chr:10	22134234	22134329	+	Functional	22134329	22134302	22134301	22134239	22134238	22134234	3
bTRAJ18	TRAJ	-	Chr:10	22135341	22135439	+	Functional	22135439	22135412	22135411	22135346	22135345	22135341	3
bTRAJ19	TRAJ	-	Chr:10	22135733	22135825	+	Pseudogene	22135825	22135798	22135797	22135738	22135737	22135733	3
bTRAJ20	TRAJ	-	Chr:10	22136702	22136792	+	Functional	22136792	22136765	22136764	22136707	22136706	22136702	1
bTRAJ21	TRAJ	-	Chr:10	22137413	22137500	+	Functional	22137500	22137473	22137472	22137418	22137417	22137413	1
bTRAJ22	TRAJ	-	Chr:10	22138937	22139032	+	Functional	22139032	22139005	22139004	22138942	22138941	22138937	3
bTRAJ23	TRAJ	-	Chr:10	22140056	22140151	+	Functional	22140151	22140124	22140123	22140061	22140060	22140056	3
bTRAJ24	TRAJ	-	Chr:10	22140497	22140592	+	Functional	22140592	22140565	22140564	22140502	22140501	22140497	3
bTRAJ25	TRAJ	-	Chr:10	22141352	22141444	+	Functional	22141444	22141417	22141416	22141357	22141356	22141352	3
bTRAJ26	TRAJ	-	Chr:10	22141650	22141742	+	ORF	22141742	22141715	22141714	22141655	22141654	22141650	3
bTRAJ27	TRAJ	-	Chr:10	22143818	22143909	+	Functional	22143909	22143882	22143881	22143823	22143822	22143818	2

bTRAJ28	TRAJ	-	Chr:10	22144466	22144564	+	Functional	22144564	22144537	22144536	22144471	22144470	22144466	3
bTRAJ29	TRAJ	-	Chr:10	22145457	22145549	+	Functional	22145549	22145522	22145521	22145462	22145461	22145457	3
bTRAJ30	TRAJ	-	Chr:10	22146520	22146611	+	Functional	22146611	22146584	22146583	22146525	22146524	22146520	2
bTRAJ31	TRAJ	-	Chr:10	22148614	22148704	+	Functional	22148704	22148677	22148676	22148619	22148618	22148614	1
bTRAJ32	TRAJ	-	Chr:10	22150419	22150517	+	Functional	22150517	22150490	22150489	22150424	22150423	22150419	3
bTRAJ33	TRAJ	-	Chr:10	22151163	22151252	+	ORF	22151252	22151225	22151224	22151168	22151167	22151163	3
bTRAJ34	TRAJ	-	Chr:10	22151853	22151942	+	Functional	22151942	22151915	22151914	22151858	22151857	22151853	3
bTRAJ35	TRAJ	-	Chr:10	22152883	22152971	+	ORF	22152971	22152944	22152943	22152888	22152887	22152883	2
bTRAJ36	TRAJ	-	Chr:10	22154348	22154441	+	Functional	22154441	22154414	22154413	22154353	22154352	22154348	1
bTRAJ37	TRAJ	-	Chr:10	22155060	22155154	+	Functional	22155154	22155127	22155126	22155065	22155064	22155060	2
bTRAJ38	TRAJ	-	Chr:10	22156562	22156656	+	Functional	22156656	22156629	22156628	22156567	22156566	22156562	2
bTRAJ39	TRAJ	-	Chr:10	22157201	22157296	+	Functional	22157296	22157269	22157268	22157206	22157205	22157201	3
bTRAJ40	TRAJ	-	Chr:10	22159313	22159402	+	Functional	22159402	22159375	22159374	22159318	22159317	22159313	3
bTRAJ41	TRAJ	-	Chr:10	22161011	22161104	+	Functional	22161104	22161077	22161076	22161016	22161015	22161011	1
bTRAJ42	TRAJ	-	Chr:10	22161480	22161578	+	Functional	22161578	22161551	22161550	22161485	22161484	22161480	3
bTRAJ43	TRAJ	-	Chr:10	22162268	22162357	+	Functional	22162357	22162330	22162329	22162273	22162272	22162268	3
bTRAJ44	TRAJ	-	Chr:10	22163570	22163664	+	Functional	22163664	22163637	22163636	22163575	22163574	22163570	2
bTRAJ45	TRAJ	-	Chr:10	22164415	22164510	+	Functional	22164510	22164483	22164482	22164420	22164419	22164415	3
bTRAJ46	TRAJ	-	Chr:10	22164927	22165022	+	Functional	22165022	22164995	22164994	22164932	22164931	22164927	3
bTRAJ47	TRAJ	-	Chr:10	22165671	22165760	+	Functional	22165760	22165733	22165732	22165676	22165675	22165671	3
bTRAJ48	TRAJ	-	Chr:10	22167691	22167786	+	Functional	22167786	22167759	22167758	22167696	22167695	22167691	3
bTRAJ49	TRAJ	-	Chr:10	22168581	22168669	+	Functional	22168669	22168642	22168641	22168586	22168585	22168581	2
bTRAJ50	TRAJ	-	Chr:10	22169468	22169556	+	Functional	22169556	22169529	22169528	22169473	22169472	22169468	2
bTRAJ51	TRAJ	-	Chr:10	22170836	22170925	+	Pseudogene	22170925	22170898	22170897	22170841	22170840	22170836	3
bTRAJ52	TRAJ	-	Chr:10	22171813	22171914	+	Functional	22171914	22171887	22171886	22171818	22171817	22171813	3
bTRAJ53	TRAJ	-	Chr:10	22175027	22175125	+	Functional	22175125	22175098	22175097	22175032	22175031	22175027	3
bTRAJ54	TRAJ	-	Chr:10	22175681	22175777	+	Functional	22175777	22175750	22175749	22175686	22175685	22175681	1
bTRAJ55	TRAJ	-	Chr:10	22176282	22176375	+	Pseudogene	22176375	22176348	22176347	22176287	22176286	22176282	1
bTRAJ56	TRAJ	-	Chr:10	22178437	22178527	+	Functional	22178527	22178500	22178499	22178442	22178441	22178437	1

bTRAJ57	TRAJ	-	Chr:10	22179066	22179161	+	Functional	22179161	22179134	22179133	22179071	22179070	22179066	3
bTRAJ58	TRAJ	-	Chr:10	22180304	22180399	+	Functional	22180399	22180372	22180371	22180309	22180308	22180304	3
bTRAJ59	TRAJ	-	Chr:10	22181469	22181554	+	Functional	22181554	22181527	22181526	22181474	22181473	22181469	2
bTRAJ60	TRAJ	-	Chr:10	22181703	22181792	+	Functional	22181792	22181765	22181764	22181708	22181707	22181703	3
bTRAJ61	TRAJ	-	Chr:10	22182669	22182762	+	Pseudogene	22182762	22182735	22182734	22182674	22182673	22182669	1

C - TRDJ genes

Name	Gene group	Gene ori.	Chromosome	Start	Stop	Chrom Ori.	Functionality	Coordinates						Reading frame
								RS		J exon		Splice site		
								Start	Stop	Start	Stop	Start	Stop	
TRDJ2	TRDJ	-	Chr:10	22201461	22201552	+	Functional	22201552	22201525	22201524	22201471	22201470	22201466	3
TRDJ3	TRDJ	-	Chr:10	22205232	22205313	+	Functional	22205313	22205286	22205285	22205227	22205226	22205222	2
TRDJ1	TRDJ	-	Chr:10	22211580	22211666	+	Functional	22211666	22211639	22211638	22211590	22211589	22211585	1

D - TRDD genes

Name	Gene group	Gene ori.	Chromosome	Start	Stop	Chrom. Ori.	Functionality	Coordinates					
								RS		D gene		RS	
								Start	Stop	Start	Stop	Start	Stop
TRDD5	TRDD	-	Chr:10	22212579	22212656	+	Functional	22212656	22212629	22212628	22212618	22212617	22212579
TRDD4	TRDD	-	Chr:10	22229689	22229764	+	Functional	22229764	22229737	22229736	22229728	22229727	22229689
TRDD3	TRDD	-	Chr:10	22238139	22238218	+	Functional	22238218	22238191	22238190	22238178	22238177	22238139
TRDD2	TRDD	-	Chr:10	22277223	22277304	+	Functional	22277304	22277277	22277276	22277262	22277261	22277223
TRDD1	TRDD	-	Chr:10	22293080	22293159	+	Functional	22293159	22293132	22293131	22293119	22293118	22293080

E - Constant genes

Name	Gene group	Gene ori.	Chromosome	Start	Stop	Chrom. Ori.	Functionality	Coordinates							
								Exon 1		Exon 2		Exon 3		Exon 4	
								Start	Stop	Start	Stop	Start	Stop	Start	Stop
TRAC	TRAC	-	Chr:10	22106925	22111855	+	Functional	22111855	22111583	22109563	22109519	22108201	22108094	22107436	22106925
TRDC	TRDC	-	Chr:10	22195089	22198861	+	Functional	22198861	22198583	22197993	22197919	22197511	22197398	22195987	22195089

Coding domains: TRAC join (22111855-22111583, 22109563-22109519, 22108201-22108095)

TRDC join (22198861-22198583, 22197993-22197919, 22197511-22197402)
