# Supplementary material for: Genomic analysis offers insights into the evolution of the bovine TRA/TRD locus
Source: BMC Genomics. 2014 Nov 19;15(1):994. doi: 10.1186/1471-2164-15-994 (PMC4289303; doi:10.1186/1471-2164-15-994)

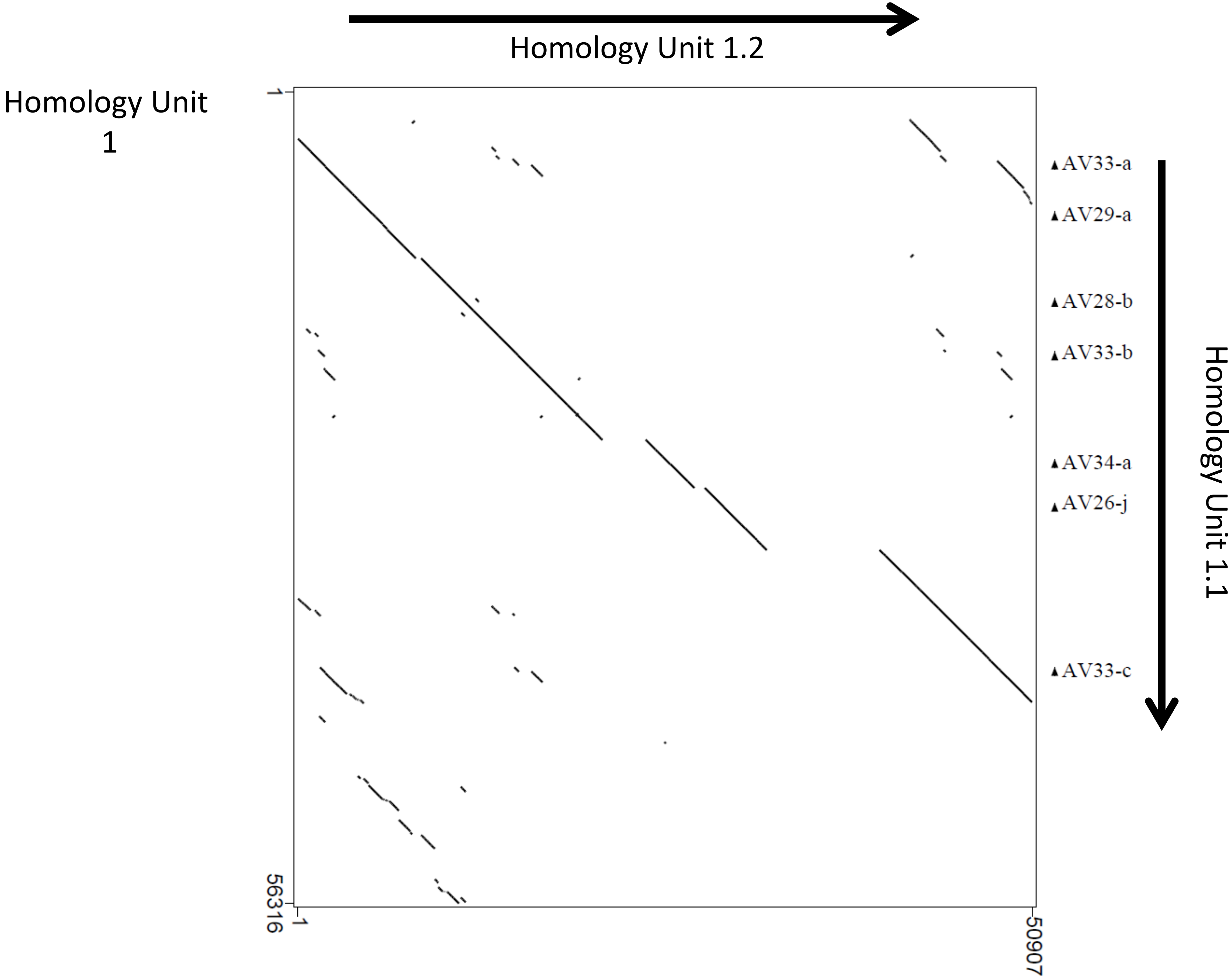

Homology Unit 1.2
Homology Unit 1
Homology Unit 1.1
1
56316
1
50907
AV33-a
AV29-a
AV28-b
AV33-b
AV34-a
AV26-j
AV33-c

Homology Unit
2

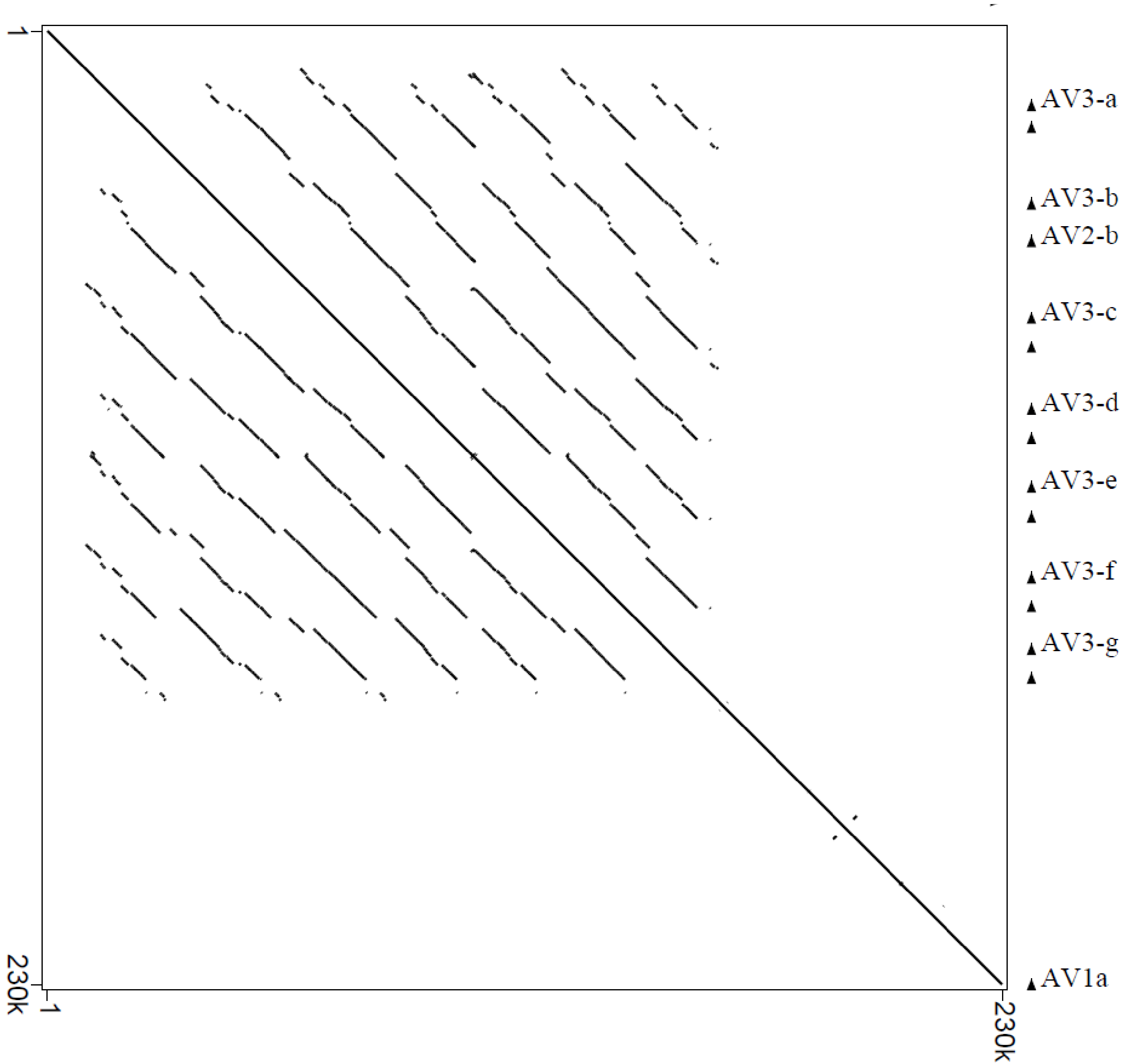

Homology Unit 3

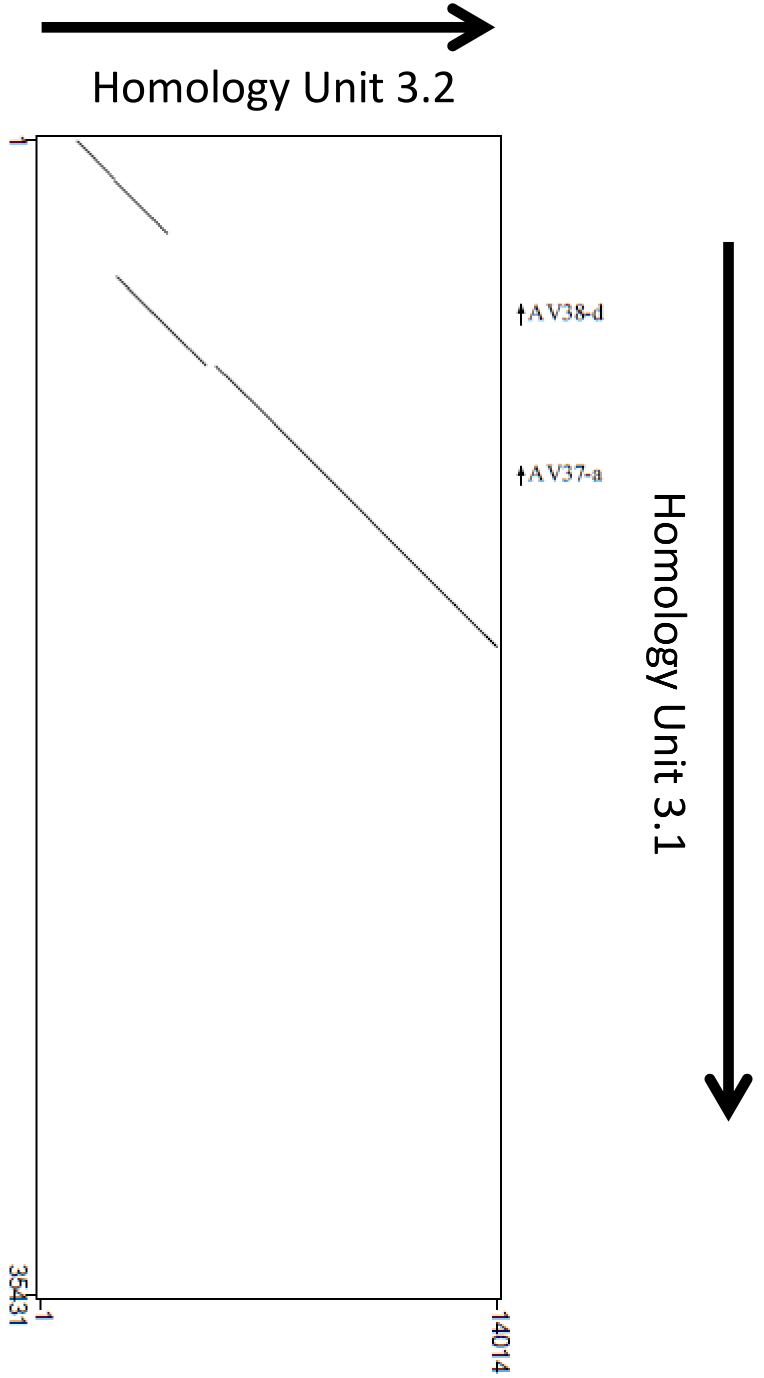

Homology Unit
4

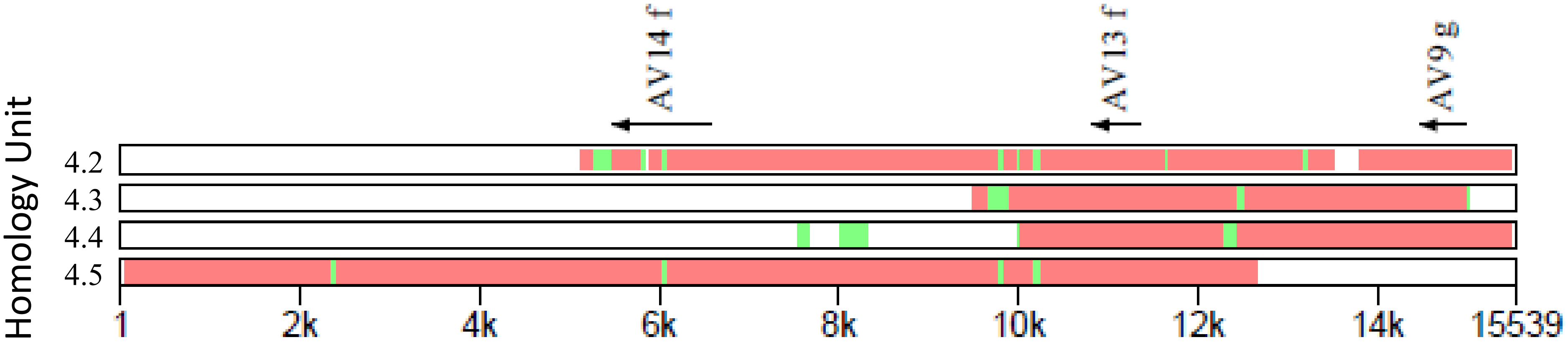

Homology Unit 5

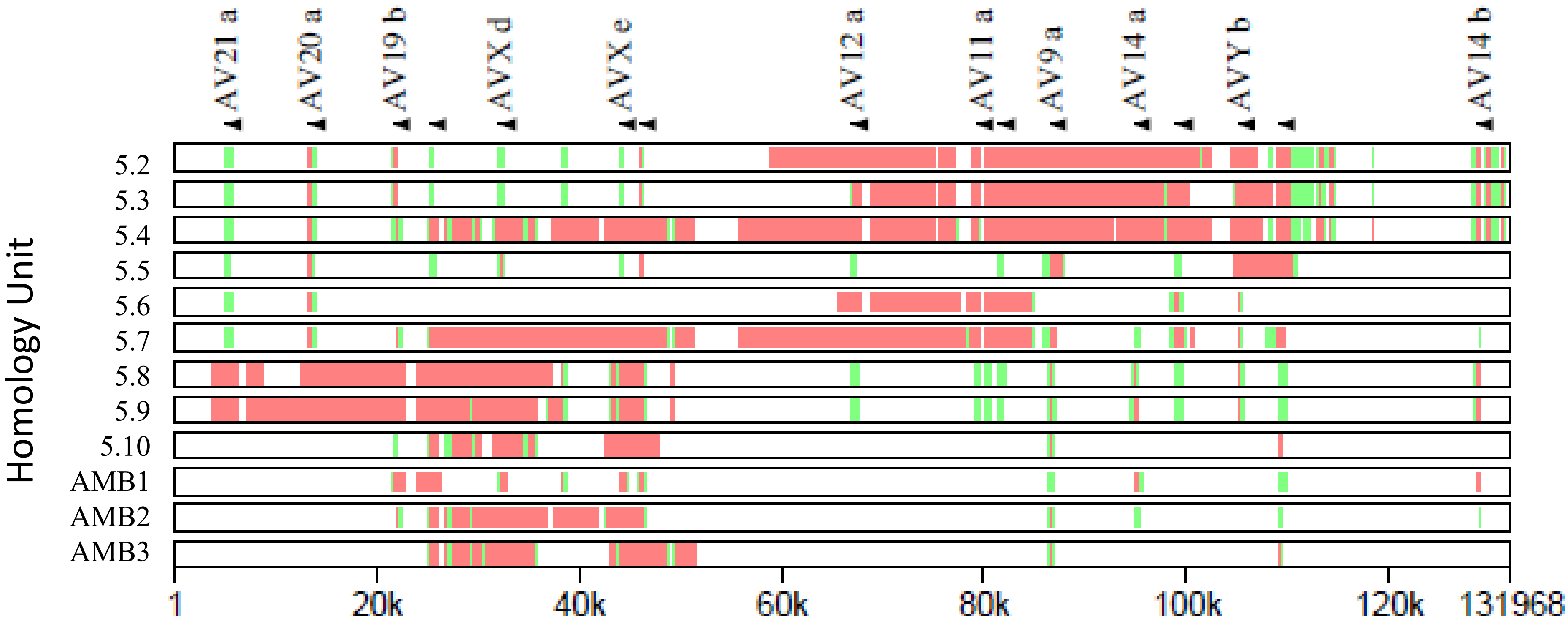

Homology Unit
6

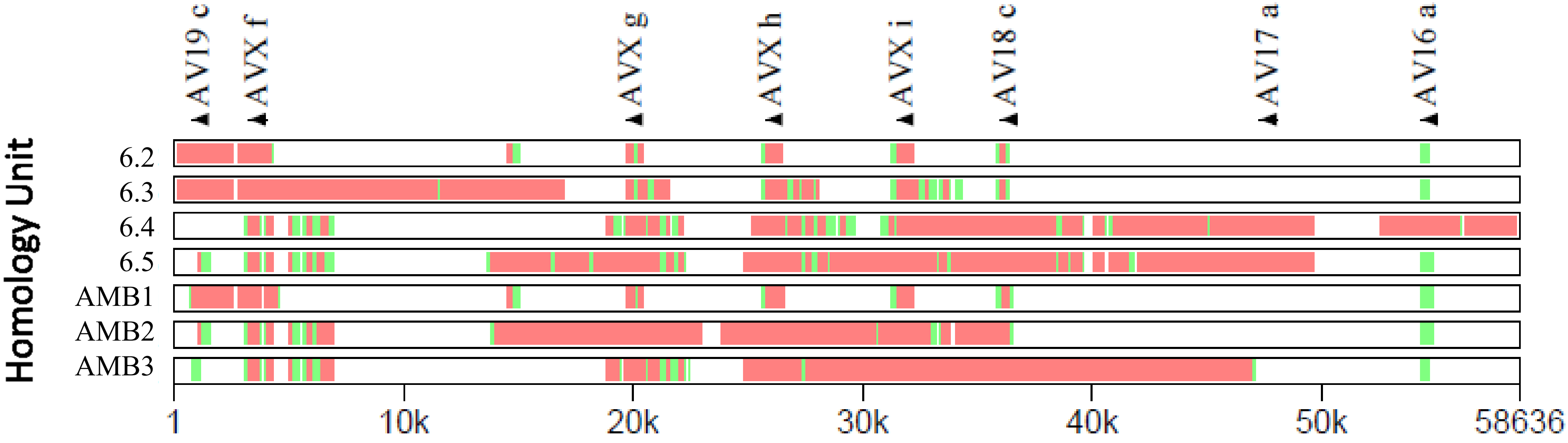

Homology Unit
7

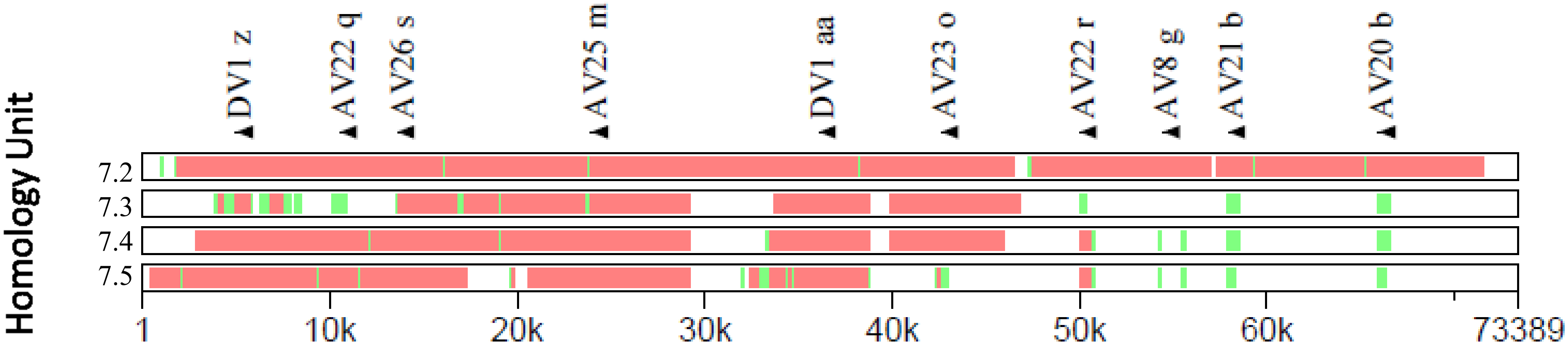

Homology Unit
8

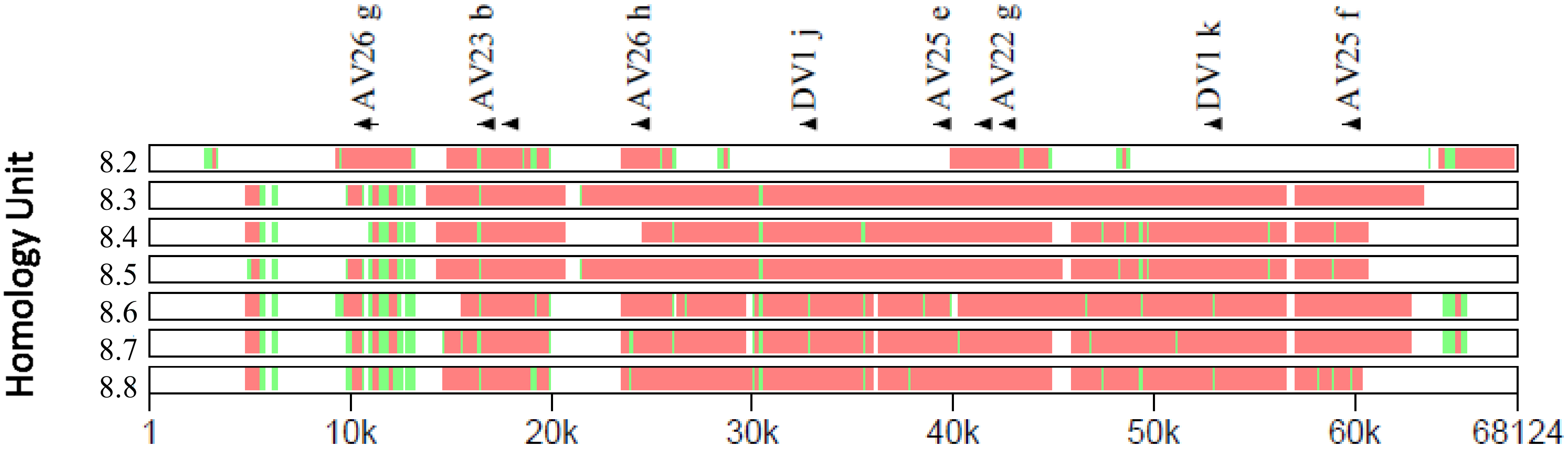

Homology Unit
9

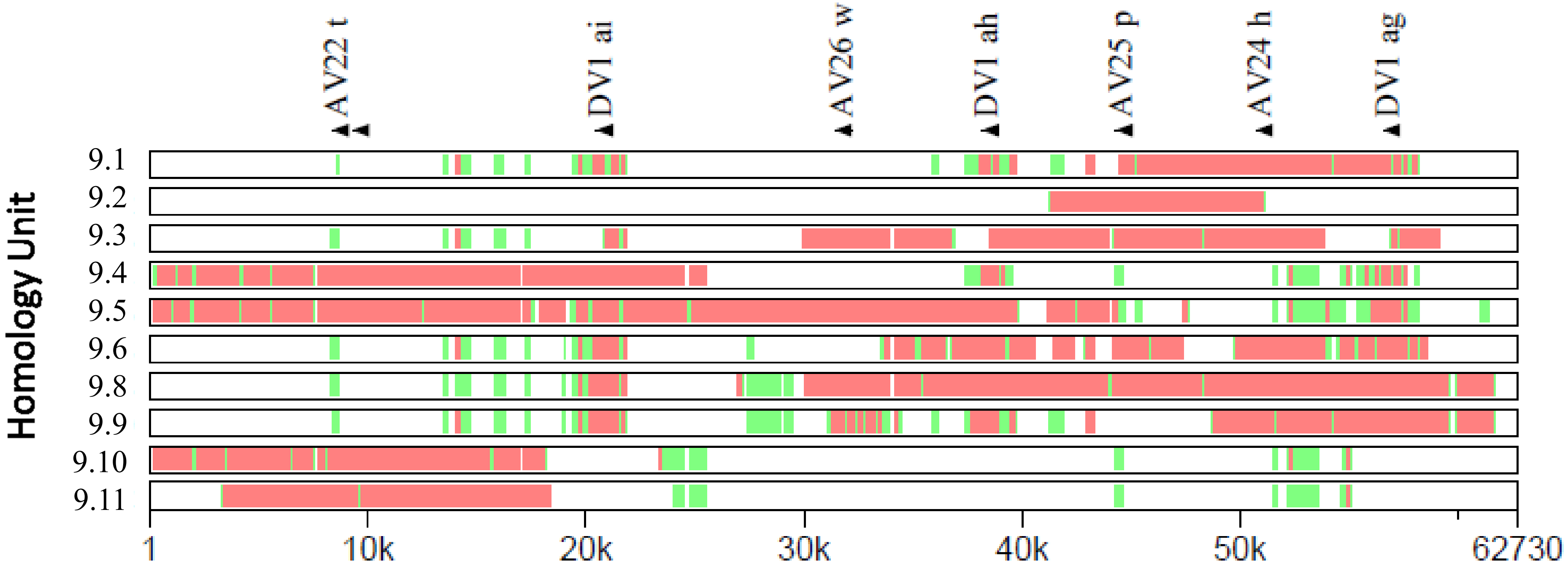

Homology Unit 10

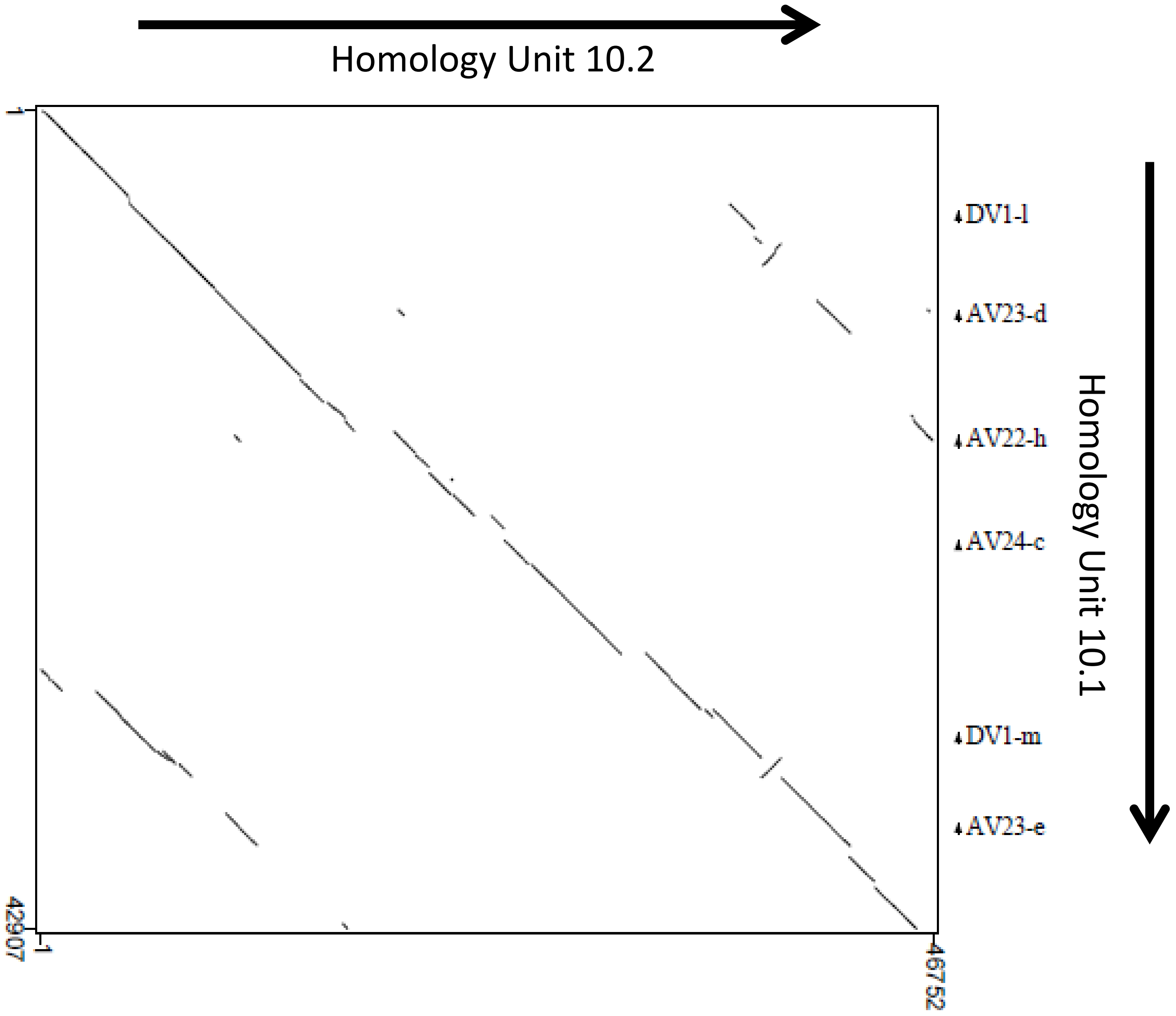

Supplement: Supplementary file 9 — Additional file 9: B – Nucleotide identity analysis of putative homology units. Nucleotide identity analysis was conducted using the Pipmaker and Multipipmaker programmes as described in Materials and Methods. For homology units with only 2 replicates (homology units 1, 3 and 10) dotplots are shown and for homology units with >2 replicates (homology units 4, 5, 6, 7, 8 and 9) a summary of the Multipip output is shown. For homology unit 2 a dotplot covering the entire region of alternating TRAV2 and TRAV3 genes against itself is shown as this best exemplifies the multiple tandem repeats that have occurred. In dotplots diagonal lines represent areas of nucleotide identity; in the Multipip output areas of high nucleotide identity are represented by red colouring. (PDF 356 KB) [file 12864_2014_6826_MOESM9_ESM.pdf]
