# Supplementary material for: Genomic analysis offers insights into the evolution of the bovine TRA/TRD locus
Source: BMC Genomics. 2014 Nov 19;15(1):994. doi: 10.1186/1471-2164-15-994 (PMC4289303; doi:10.1186/1471-2164-15-994)
Supplement: Supplementary file 10 — Additional file 10: A Genomic sequence of the bovine TRAJ genes identified in UMD3.1. The table shows the (i) RS sequence, (ii) reading frame of the coding sequence, (iii) nucleotide and predicted amino acid sequences of the coding sequence and (iv) the sequence of the 3′ splice site. The canonical FGxG motif of the TRAJ sequence is shown with yellow highlighting. TRAJ genes that are predicted to be pseudogenes are shown with their name and the identified defect highlighted in red. B - Summary of non-functional TRAJ gene segments in UMD3.1. Summary of the lesions identified in TRAJ genes that are considered to render them non-functional. (PDF 288 KB) [file 12864_2014_6826_MOESM10_ESM.pdf]

A

Gene name	RSS (i)			Reading Frame (ii)	Coding sequence (iii)	Splice site (iv)
	Nonamer	Spacer	Heptamer			
bTRAJ1	GCTTTCTGT	AATGAAGACAGT	GAGAGTG	2	AGATGGAGGTGTTGGCTCCCAGATGCAGTTTGGCAAGGGAACCAGAGTCTCCATCACTCCAG<br>D G G V G S Q M Q F G K G T R V S I T P	GTATG
bTRAJ2	AGTTTCTGT	AATGGTGTCCCC	AGCAGTG	3	TGAATACAGGAGGAGCGATCGGTAAACTCACTTTTGGGAAAGGAACCCAAGTGTCCATAATATCTG<br>N T G G A I G K L T F G K G T Q V S I I S	GTGAG
bTRAJ3	GGTTATTGC	AAAGACCTTACC	CTCAGTG	2	GAGATTCGGCACTGATAAGTTAAACTTCGGAGCAGGGACCAGACTGAGTGTCCATCCAA<br>R F G T D K L N F G A G T R L S V H P	GTGAG
bTRAJ4	GCATCCTCC	TAGTGGGTGTCT	TCTGGTG	3	TCTACAATAAGCTGACCTTTGGAACCGGAACCAGGCTGACTGTACACTCAC<br>Y N K L T F G T G T R L T V H S	GTGAG
bTRAJ5	AGTATTTGT	ACTGCATTGTGG	CAGGGTG	3	TGAACACAGGCAGCAGAACACTCACTTTTGGGAGTGGAACAAGACTCCGAGTGCATCCAA<br>N T G S R T L T F G S G T R L R V H P	GTAAG
bTRAJ6	GGTTTTAGC	AAGGGCTTTCCT	TGCTGTG	2	TGTATCAAGACTAAAGTATGGATTTATATTTGGAACAGGGACCAAGCTTGTTGTTCACCCAC<br>V S R L K Y G F I F G T G T K L V V H P	GTGAG
bTRAJ7	TGTTTTTGT	AATGCACTTACC	CAGAGTG	2	TGATTATGGGAACAGACTCACTTTTGGAAATGGGACTCGAGTGCTGGTCACACCAA<br>D Y G N R L T F G N G T R V L V T P	GTAAG
bTRAJ8-1	CCATTTTGT	ATAGAGTTATGT	CAGAGTG	3	TGAGCACAGGTTATCAGAAATTCGTATTTGGAACTGGCACCCAACTTTTGATCAACCCAA<br>S T G Y Q K F V F G T G T Q L L I N P	GTAAG
bTRAJ9	CCATTTTGT	CACAGCACAAAT	CACTGTG	1	GGATATACTGGAGGCTTCAAATTTGTTTTTGGGACAGGAACAAAGCTATTTATTGAAACAA<br>G Y T G G F K F V F G T G T K L F I E T	GTAAG
bTRAJ8-2	CCATTTTGT	ACAGAGTTATGT	CAGAGTG	3	TGAACACAGGTTATCAGAAACTCACATTTGGAACTGGCACCCAACTTTTAATCAACCCAA<br>N T G Y Q K L T F G T G T Q L L I N P	GTAAG
bTRAJ10	AGTTTATTG	TGAGGCATCAAA	CACTGTG	3	GGAATTGGGGAGGAGGAAACAAACTCATCTTTGGGAAAGGCACCCACCTACAAGTGAAGCTGA<br>N W G G G N K L I F G K G T H L Q V K L	GTAAG
bTRAJ11	TATTTTTGT	ATGGGGGTTTGC	TACAGTG	3	TGAATTCAGGATACAACACACTTACTTTTGGAAAGGGCACTGTGCTTCTTGTCTTTCCAG<br>N S G Y N T L T F G K G T V L L V F P	GTAAA
bTRAJ12	GGTTTTTGA	CTGACTAAGAAA	CACTGTG	3	GGAAGGATGGAGGCTATACATGGACCTTCGGAAGTGGGACTAGACTGCTGGTCAGGCCTG<br>K D G G Y T W T F G S G T R L L V R P	GTGAG
bTRAJ13	CCATCCTGT	ATAGGCCTGCAT	TGTAGCG	3	TGAATCCTAGGGGTGACCGAAAGGTTGCCTTTGGAACTGGAACGACACTTCAAGTCACCCCAA<br>N P R G D R K V A F G T G T T L Q V T P	GTAAG
bTRAJ14	AATTATTGT	CAGGCAGCACGG	TGCTGTG	1	ATTTATAACACATTCATCTTTGCGAGTGGGACAAGATTATCAGTAAAACCCA<br>I Y N T F I F A S G T R L S V K P	GTAAG
bTRAJ15	GGTATTTGC	AGGACCTTGTTT	CACTGTG	3	CCTACCAGGGAGGAAATGCATTGGTCTTTGGGAAAGGAACCACAGTATCAGTGCATCCCA<br>Y Q G G N A L V F G K G T T V S V H P	GTAAG
bTRAJ16	GGTTTTTGT	GGTGAAGTAGAT	CACTGTG	1	GGGTTTTCAAGTGGCCAGAAGTTGGTTTTTGGAAGTGGGACCATGTTAAAGGTGAATCTTA<br>G F S S G Q K L V F G S G T M L K V N L	GTAAG
bTRAJ17	GGTTTTTGC	TGGGCCTTGAAT	CACTGTG	3	TGACCACCACTGCAGGGACCAAGCTAACCTTTGGAGAAGGAACCAGGCTGATAGTCAAGCTGA<br>T T T A G T K L T F G E G T R L I V K L	GTGAG
bTRAJ18	GGTTCTTGT	AAAGGGGGCCGG	CACTGTG	3	TCAATAGAGGCTCAAGTCTGGGGAGGTTCTACTTTGGAAGAGGAACCCGGCTAACTGTACAGCCTG<br>N R G S S L G R F Y F G R G T R L T V Q P	GTGAG

Gene name	RSS (i)			Reading Frame (ii)	Coding sequence (iii)	Splice site (iv)
	Nonamer	Spacer	Heptamer			
bTRAJ19	TGATTCTGC	AAAGGACTGCAA	AGGACAG	3	TCTATCAGAGTTTTCGCAAGTTCACCTTTGGAAAAGGATCCAAACACAATGACAATCCAA<br>Y Q S F R K F T F G K G S K H N D N P	CTAAG
bTRAJ20	GGTTTGCGT	AGGAAGACGTAG	CACTGTG	1	ACTCTAAATAACTACAAGCTCACCTTTGGATCAGGAACCACAGTAACTGTGAGAGCAA<br>T L N N Y K L T F G S G T T V T V R A	GTAAG
bTRAJ21	ACTTTTTGT	AATGGTAGTAAA	CATGGTG	1	TATAACACCAACAGATTTTACTTCGGATCTGGGACAAAACTCAGAGTAAAGCCAA<br>Y N T N R F Y F G S G T K L R V K P	GTAAG
bTRAJ22	GGTTTTTGT	TGTTGGGCTTAT	CACAGTG	3	CTTCCTCTAGTTCAGGCTGGCAACTGACCTTTGGATCTGGGACCCAACTGACTGTTGTACCTG<br>S S S S G W Q L T F G S G T Q L T V V P	GTAGG
bTRAJ23	TGTTTTTGA	CAGGCTGTATAA	CACTGTG	3	TGAATTATAACCAGGGAGGAAAGCTTATCTTCGGACAGGGAACCGAGTTATCTGTGAAGCCCA<br>N Y N Q G G K L I F G Q G T E L S V K P	GTAAG
bTRAJ24	CCATTTTGT	AGAGGTGTTTGT	CACAGTG	3	TGACAACTGACGGCTGGGGGAAATTGAATTTTGGAGCGGGGACCCGGGTTGTGGTCACCCCAG<br>T T D G W G K L N F G A G T R V V V T P	GTAAG
bTRAJ25	GGTTTTTGA	TGCTGAGATAAT	CACTGTG	3	GGGAAGGACAAGGCTTCTCCCTCGTCTTTGGGAAGGGGACAAGGCTGCTTGTCAAGCCGA<br>E G Q G F S L V F G K G T R L L V K P	GTAAG
bTRAJ26	GCTTTTTGT	AAAGCCCAGGGG	CACTGTG	3	GGAATAACTATGGCCAGGGTTTGATCTTCAGTGGAGGAACCAGACTGTCTGTGCAGCCCC<br>N N Y G Q G L I F S G G T R L S V Q P	GTAAG
bTRAJ27	GGTTATTGC	AATAGCACTGAA	CACTGTG	2	TAACACCGGTACAGGCAAATTAACCTTTGGGGACGGGACTGCGCTCACTGTGAAGCCGA<br>N T G T G K L T F G D G T A L T V K P	GTAAG
bTRAJ28	GGTTTTTGC	AAAGAAAGGAAA	CTCTGTG	3	CCTACTCTGGGACTGGGAGTTACCAACTCACCTTCGGGAAGGGCACCAAACTCTTGGTCACACCAA<br>Y S G T G S Y Q L T F G K G T K L L V T P	GTAAG
bTRAJ29	AGTTTTTGT	GATGAAGGCAAT	CACTGTG	3	GGGATTCAGGAAACAGGCAACTTGTCTTTGGAAAAGGCACAAGACTTGCTGTGACTCCAA<br>D S G N R Q L V F G K G T R L A V T P	GTAAG
bTRAJ30	AGTTTTTGT	TGTGGCCCCAAT	CACAGTG	2	TGAACAAACCTCTGGCAAAATTGTCTTTGGAAGAGGGACTCAGCTTCATGTTCTCCCCA<br>E Q T S G K I V F G R G T Q L H V L P	GTAAG
bTRAJ31	GGTTTCAGT	AAAAGCAGGAAA	TGCTGTG	1	GGGACTGACAATTCCAGAATCTTCTTTGGAACTGGAACCCAGGTGGTGGTAAAGCCCA<br>G T D N S R I F F G T G T Q V V V K P	GTAAG
bTRAJ32	GGTTGTTGT	AAGGCACTGAAA	GGCTGTG	3	TGAATTATGGGGGTGCTGCCAACCAGCTCATCTTTGGGACTGGCACGCTGCTTTCTGTCAAGCCAA<br>N Y G G A A N Q L I F G T G T L L S V K P	GTACG
bTRAJ33	GGTTTTTGT	TAAGGTTTTTGT	CTCTGTG	3	TGGATGGCAACTATCAGTGGATCTGGGGCTCTGGGACCAAACTAATTATAAAGCCAG<br>D G N Y Q W I W G S G T K L I I K P	GTAAG
bTRAJ34	GGTTTTTGT	AGGCCTTGGTAT	CACTGTG	3	TCTCCAACAGGGACAAACTCATCTTTGGGGCTGGAACCAGATTACAAGTTTTTCCAA<br>S N R D K L I F G A G T R L Q V F P	GTAAG
bTRAJ35	GGTTTTTGT	AGTGGAGTCAGC	CATGGTG	2	GAACTTTGGGAATGTGCTGCATTTGGGGTCTGGCACTCAAGTGATCGTTATGCCTC<br>N F G N V L H L G S G T Q V I V M P	GTAAG
bTRAJ36	TGTTTTTGT	ACTGGGCAGAAA	CACTGTG	1	TTAACTTCTGGAGCAAACCAGCTTGTCTTTGGGACAGGAACAAGCCTCACCATCCTCCCCC<br>L T S G A N Q L V F G T G T S L T I L P	GTAAG
bTRAJ37	AGTTTTTGT	AAAGTAGAGCAT	TACAGTG	2	TGACTCAGGAAACACAGGAAGACTCACCTTTGGGCAGGGGACAATGTTACAAGTAAAACCAG<br>D S G N T G R L T F G Q G T M L Q V K P	GTAGG
bTRAJ38	GGTTTCTGT	AAAGCTTTCCAT	GACTGTG	2	TAATACTGGCAACAACCGTAAGCTGATTTGGGGACTGGGGACAAGTCTGGCAGTAAATCCAA<br>N T G N N R K L I W G L G T S L A V N P	GTGAG

Gene name	RSS (i)			Reading Frame (ii)	Coding sequence (iii)	Splice site (iv)
	Nonamer	Spacer	Heptamer			
bTRAJ39	GGTTTTTGC	TGAGCTGAAGAT	CACTGTG	3	TGAATAATAATGCAGGCAACATATTCACATTTGGAGGGGGAACAAGGTTAATGGTCAAACCTC<br>N N N A G N I F T F G G G T R L M V K P	GTGAG
bTRAJ40	GGTTTATGT	AGAGCCACATAG	CACTGTG	3	ACTCAGGAAACTACAAATACGTCTTTGGAGCGGGCACTAGGCTGCAGGTTTTAACAA<br>S G N Y K Y V F G A G T R L Q V L T	GTGAG
bTRAJ41	GGTTTTTGT	TTAGGGAAGGTG	CACTGTG	1	GAACTCAATGCCGGCTACGCGCTCCACTTTGGCCAAGGCACCTCAGTGCTGGTCACACCCG<br>E L N A G Y A L H F G Q G T S V L V T P	GTGAG
bTRAJ42	GGTAATTGT	AAGGTCCCATAT	GACTGTG	3	TGAATGTTGGCAGCGGCCAAGGACAGCTCATCTTTGGAAAAGGCACTATGGTCTCTGTTAAACCAA<br>N V G S G Q G Q L I F G K G T M V S V K P	GTAAG
bTRAJ43	GGTTTTTGT	TAGAGGGTGTAC	TGCTGTG	3	GCAATAATAACAACAACCTACGCTTTGGAGCAGGGACCAGACTGACGGTAAAACCAA<br>N N N N N L R F G A G T R L T V K P	GTAAG
bTRAJ44	GGTTTCTGT	CATGGAGCATCT	CACAGTG	2	TAACACGGGCAGTGGCGGGAAAGTCATATTTGGGACTGGAACGAGACTTCAGGTCACCCTTG<br>N T G S G G K V I F G T G T R L Q V T L	GTAGG
bTRAJ45	GATTTATGT	AAAGGGTTAACA	TGGGGTG	3	TGAATACAGGAGGAAGTAACAGACTCATCTTTGGAAAAGGGACTCAGCTCATCATCCAGCCCC<br>N T G G S N R L I F G K G T Q L I I Q P	GTAAG
bTRAJ46	CGTTTCTGT	AAAGCTGCTGAC	AACAGTG	3	AGAAGAGTGGCAGCGGAGACAGGCTGACTTTTGGGACCGGGACGCGTTTAGCAGTGAGGCCCA<br>K S G S G D R L T F G T G T R L A V R P	GTAAG
bTRAJ47	TGTTTTTGT	AGAGGAGTTTGA	TGCTGTG	3	TGAATTCTGGAAACACACTGATCTTTGGCTCAGGAACCACTTTGACAGTCAAGCCTC<br>N S G N T L I F G S G T T L T V K P	GTGAG
bTRAJ48	GGTTTTTGT	AATGACTTAGAA	CACTGTG	3	TGTCTAACTACCAAGGCAGTCAATTAAACTTTGGAACAGGAACTAGACTCACCATCACAGCCA<br>S N Y Q G S Q L N F G T G T R L T I T A	GTAAG
bTRAJ49	GGTTTTTGT	TGAGCTTCCTAT	CACAGTG	2	GAACAGCTACGGCCAGAACTATTTTGGGAGAGGGACAAGTTTGACAGTCATTCCAA<br>N S Y G Q N Y F G R G T S L T V I P	GTAAG
bTRAJ50	AGTTATTGC	AAAGTGTTCGAT	GGCTGTG	2	TGACAACTCCTACAAGTTGATGTTTGGGCAAGGGACGAGCTTATCAGTCATTCCAA<br>D N S Y K L M F G Q G T S L S V I P	GTAAG
bTRAJ51	AGTCGTTGT	AAAACTCTCCAC	TGCAGGG		AGTAGCACGGCAGCTCTGAGTTAGAAAAAGAAAGAACTTGACTAACCGTGAAGCCAA	GAAGC
bTRAJ52	GGTTCTTGT	AAAGGCTTCCAT	TGCAGTG	3	TAAATACTGGTGGTAGTGCCTATGGAAAGCTGATGTTCGGACAAGGAACCACTTTGACTGTCCATCCAA<br>N T G G S A Y G K L M F G Q G T T L T V H P	GTAAG
bTRAJ53	TGTTTTTGT	AAAGCCTCCCCT	GACTGTG	3	AGACTAGTGGAACTAGCAGCTATAAACAGACATTTGGAAAAGGCACTCTCTTAATTGTGAATCCAA<br>T S G T S S Y K Q T F G K G T L L I V N P	GTAAG
bTRAJ54	AGTTTGTGT	AAAGCTCTTTGC	TGGGGTG	1	TGACTCAGAGGTGCAGGCCAGAAGCTGGTATTCGGGCGAGGAACCAGGCTGACCATCAACCCCA<br>* L R G A G Q K L V F G R G T R L T I N P	GTAGG
bTRAJ55	TGATGCTGA	GCAGGGCTCCCT	GTTGGTG		ACAAAAGATAGCAATGTCCCGGTTGGAGAGGAGAAGAGAATAAGTCCAAAAATAAGTGCAA	ATAAG
bTRAJ56	AGTTTTTGT	AGAGTCTCGTGT	CATTGTG	1	TGATACTCCAATAATAAGCTGACATTTGGAAAAGGAACAATCCTGAATGTTAGACCAG<br>* Y S N N K L T F G K G T I L N V R P	GTATG
bTRAJ57	GGTATTTGT	AAAGCAGTCTAT	GGGGGTG	3	TAACTCAGGGCGGATCTGAAAGACTCCTCTTTGGAAAGGGAACGAAACTGACAGTAAACCCAC<br>T Q G G S E R L L F G K G T K L T V N P	GTAAG
bTRAJ58	GGTTTTTGC	AAAGCCTTGGAG	CATTGTG	3	TTCAACAAACCAGTGGCTCTAAGATGATATTTGGGAAAGGGACTCAGCTCACAGTGCAACTTG<br>Q Q T S G S K M I F G K G T Q L T V Q L	GTAAG

Gene name	RSS (i)			Reading Frame (ii)	Coding sequence (iii)	Splice site (iv)
	Nonamer	Spacer	Heptamer			
bTRAJ59	AGTTTATGT	AAAGGTGTCTGC	TCCTGTG	2	GGAAGCAAGGCAATTTACATTCGGAACCGGGACTCAAGTGAGAGTGAAACTTA<br>E A R Q F T F G T G T Q V R V K L	GTAAG
bTRAJ60	ACTTTTGGT	AAAGGGCCCAGG	CACTGTG	3	TAAAGAGCACTGAGAAATTCACCTTTGGGAAAGGGACTCAGTTAATTGTAAGCCTGG<br>K S T E K F T F G K G T Q L I V S L	GTGAG
bTRAJ61	AGTTTTTGT	AAAGGTGTTCAC	TCCTGTG		GGAATGGGGTTAATAGGAAACTGACATTTTGGAACCAAGACTAGAGGAATCTTGAAACTCA	GTGAG

B

bTRAJ	Stop codon in coding region	Loss of canonical FGxG motif	Defective splice site	RS defect
bTRAJ4				•
bTRAJ13				•
bTRAJ14*		•		
bTRAJ19			•	•
bTRAJ26*		•		
bTRAJ33*		•		
bTRAJ35*		•		
bTRAJ51	•		•	•
bTRAJ55	•		•	
bTRAJ61	•			

* These genes have been classified as non-functional due to loss of the canonical FGxG motif. However, the functionality of these genes may have to be determined empirically; hTRAJ16 is considered functional despite absence of the FGxG motif, whilst mTRAJ44 and 47 are considered non-functional due to absence of the FGxG motif.
