# Supplementary material for: Genomic analysis offers insights into the evolution of the bovine TRA/TRD locus
Source: BMC Genomics. 2014 Nov 19;15(1):994. doi: 10.1186/1471-2164-15-994 (PMC4289303; doi:10.1186/1471-2164-15-994)
Supplement: Supplementary file 11 — Additional file 11: Summary of non-functional TRAV/TRDV gene segments in UMD3.1. The lesions identified in TRAV/TRDV genes which are considered to render them non-functional are summarised. (PDF 584 KB) [file 12864_2014_6826_MOESM11_ESM.pdf]

TRA/DV gene	No initiation codon	Frameshift in L exon	Stop codon in L exon	Frameshift in V exon	Stop codon in V exon	Defective splice sites	Loss of conserved amino acids	RS defect	Partial	Orphon
bTRAV2-d				•						
bTRAV2-e				•						
bTRAV4-c					•					
bTRAV6-b									•	
bTRAV6-c				•				•		
bTRAV6-d				•				•		
bTRAV8-a				•						
bTRAV8-b		•		•				•		
bTRAV8-c				•		•				
bTRAV8-d				•						
bTRAV8-e	•			•						
bTRAV8-h				•		•				
bTRAV8-k	•			•		•		•		
bTRAV8-m				•						
bTRAV9-a				•	•	•		•		
bTRAV9-b								•		
bTRAV9-c						•		•		
bTRAV9-e	•	•								
bTRAV9-f				•		•				
bTRAV9-g				•						
bTRAV9-h		•								
bTRAV9-i		•		•		•				
bTRAV9-j		•		•		•		•		
bTRAV10-a	•	•							•	
bTRAV10-c									•	
bTRAV10-f										
bTRAV11-a				•						
bTRAV11-b	•	•					•			
bTRAV11-c		•						•		
bTRAV11-d					•		•	•		
bTRAV11-e					•			•		
bTRAV11-f					•			•		
bTRAV13-a				•						
bTRAV13-c				•						
bTRAV14-b				•						
bTRAV14-c				•	•					
bTRAV14-d					•					
bTRAV14-f					•	•				
bTRAV17-b		•								
bTRAV17-c					•					
bTRAV18-a				•						
bTRAV18-c							•			
bTRAV18-e								•		
bTRAV18-f						•				
bTRAV18-g						•				
bTRAV19-b				•						
bTRAV19-e				•						
bTRAV21-d						•				
bTRAV22-a					•					
bTRAV22-c					•			•		
bTRAV22-e									•	
bTRAV22-f				•						

bTRAV22-g				•						
bTRAV22-j					•					
bTRAV22-k									•	
bTRAV22-l							•			
bTRAV22-m					•					
bTRAV22-o				•						
bTRAV22-p						•				
bTRAV22-r						•				
bTRAV22-v		•		•						
bTRAV-22y									•	
bTRAV23-a			•	•		•		•		
bTRAV23-b				•				•		
bTRAV23-c		•		•				•		
bTRAV23-d				•						
bTRAV23-e				•						
bTRAV23-h		•						•		
bTRAV23-i					•			•		
bTRAV23-j					•			•		
bTRAV23-k				•				•		
bTRAV23-l				•				•		
bTRAV23-m				•						
bTRAV23-n				•		•				
bTRAV23-p									•	
bTRAV23-q					•					
bTRAV23-r									•	
bTRAV23-s	•	•			•			•		
bTRAV23-t					•					
bTRAV23-u				•						
bTRAV23-v			•		•					
bTRAV23-w	•		•	•				•		
bTRAV24-a						•				
bTRAV24-c									•	
bTRAV24-e				•		•				
bTRAV24-f									•	
bTRAV24-g						•				
bTRAV24-h					•	•				
bTRAV24-i	•									
bTRAV24-j					•					
bTRAV25-c				•						
bTRAV25-d			•							
bTRAV25-e				•						
bTRAV25-f				•						
bTRAV25-g			•							
bTRAV25-n				•						
bTRAV25-o					•					
bTRAV25-q							•			
bTRAV25-s					•					
bTRAV25-u					•					
bTRAV26-d						•				
bTRAV26-f				•						
bTRAV26-g					•					
bTRAV26-h				•						
bTRAV26-j									•	
bTRAV26-o				•						
bTRAV26-q						•				

bTRAV26-v	•				•		•			
bTRAV26-y				•						
bTRAV26-z				•			•			
bTRAV26-ab	•									
bTRAV26-af				•						
bTRAV26-am	•				•	•				
bTRAV28-d									•	
bTRAV29-a				•						
bTRAV33-b									•	
bTRAV33-c	•			•		•		•		
bTRAV33-e									•	
bTRAV33-f	•			•		•		•		
bTRAV34-a				•	•	•	•			
bTRAV34-b				•	•	•				
bTRAV34-c				•	•	•	•			
bTRAV37-a		•		•						
bTRAV37-b			•	•	•					
bTRAV39-a							•			
bTRAVX-a				•						
bTRAVX-t		•								
bTRAVX-v	•							•		
bTRAVX-y				•						
bTRAVX-ac			•		•					
bTRDV1-d	•	•				•				
bTRDV1-r	•									
bTRDV1-v				•						
bTRDV1-ad				•				•		
bTRDV1-ap					•					
bTRDV1-at					•					
bTRDV1-av				•	•					
bTRDV1-bh										•
